# Supplementary material for: Persistence of RNA transcription during DNA replication delays duplication of transcription start sites until G2/M
Source: Cell Rep. 2021 Feb 16;34(7):108759. doi: 10.1016/j.celrep.2021.108759 (PMC7900609; doi:10.1016/j.celrep.2021.108759)
Supplement: Document S1. Figures S1–S7 and Tables S1–S4 [file mmc1.pdf]

Cell Reports, Volume *34*

Supplemental Information

Persistence of RNA transcription during DNA replication delays duplication of transcription start sites until G2/M

Jianming Wang, Patricia Rojas, Jingwen Mao, Martina Mustè Sadurnì, Olivia Garnier, Songshu Xiao, Martin R. Higgs, Paloma Garcia, and Marco Saponaro

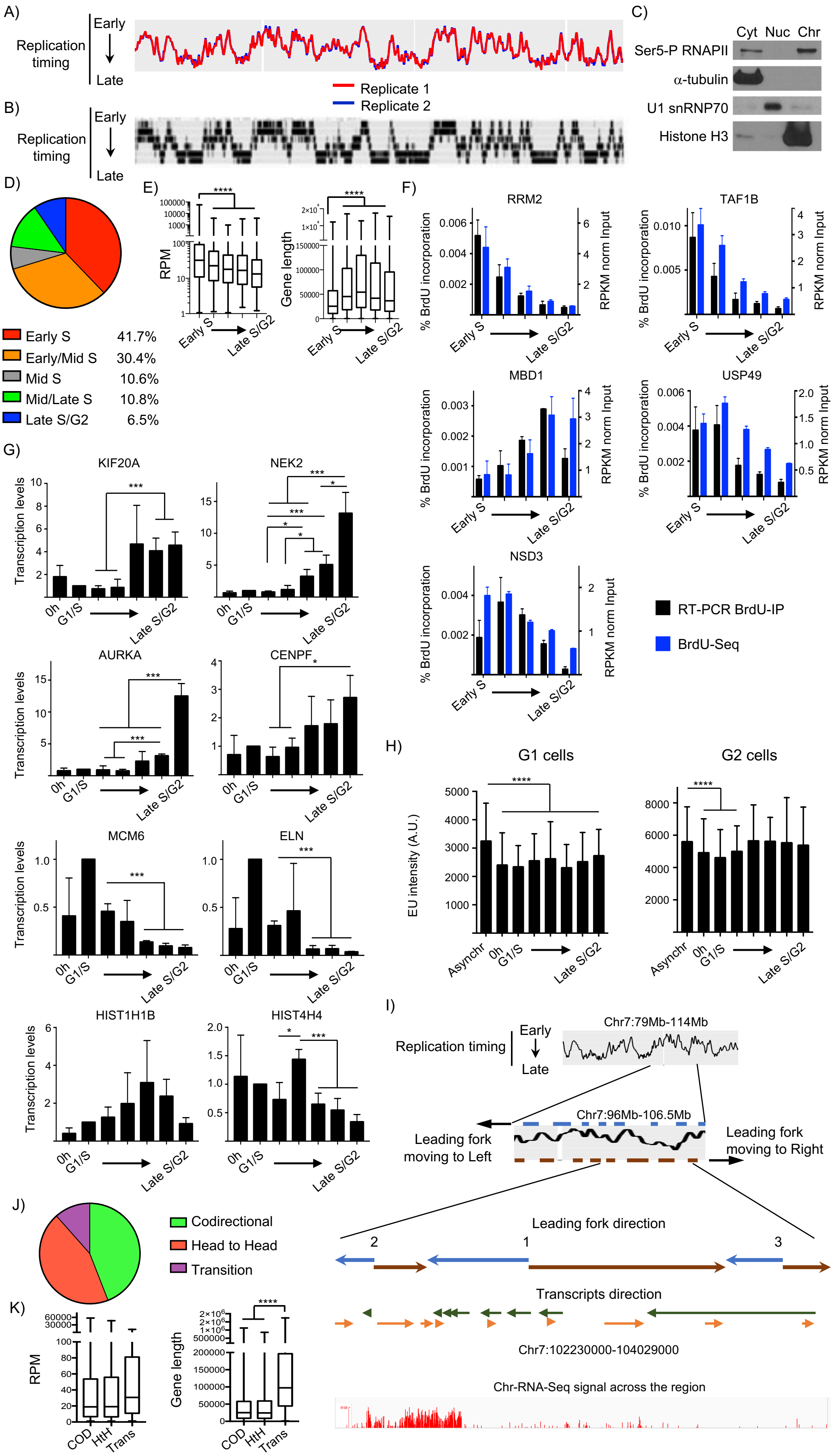
A)
Replication timing
Early
Late
Replicate 1
Replicate 2
B)
Replication timing
Early
Late
C)
Cyt
Nuc
Chr
Ser5-P RNAPII
α-tubulin
U1 snRNP70
Histone H3
D)
Early S 41.7%
Early/Mid S 30.4%
Mid S 10.6%
Mid/Late S 10.8%
Late S/G2 6.5%
E)
RPM
Gene length
Early S
Late S/G2
****
F)
RRM2
TAF1B
MBD1
USP49
NSD3
% BrdU incorporation
RPKM norm Input
Early S
Late S/G2
RT-PCR BrdU-IP
BrdU-Seq
G)
KIF20A
NEK2
AURKA
CENPF
MCM6
ELN
HIST1H1B
HIST4H4
Transcription levels
0h
G1/S
Late S/G2
H)
G1 cells
G2 cells
EU intensity (A.U.)
Asynchr
0h
G1/S
Late S/G2
I)
Replication timing
Early
Late
Chr7:79Mb-114Mb
Chr7:96Mb-106.5Mb
Leading fork moving to Left
Leading fork moving to Right
Leading fork direction
Transcripts direction
Chr7:102230000-104029000
Chr-RNA-Seq signal across the region
J)
Codirectional
Head to Head
Transition
K)
RPM
Gene length
COD
HtH
Trans

Figure S1. Genomic approaches to characterize transcription and replication together, Related to Figure 1

(A) Replication timing profiles from early to late S phase for the long arm of chromosome 7 shown in Fig 1B, and their reproducibility in two biological replicates. (B) Image adapted from *Hansen et al., 2010*, showing that our approach produces data similar to previously published Repli-Seq ones, but with higher resolution. (C) Representative western blotting analysis for the validation of the cellular fraction strategy in BJ-hTERT cells as in *Mayer et al., 2015*. Subcellular localization markers were α-tubulin for cytoplasm (Cyt), U1 snRNP70 for nucleoplasm (Nuc) and histone H3 for chromatin (Chr), plus transcriptionally engaged Ser5-P RNAPII. (D) Timing of replication for actively transcribed genes (RPM>1). (E) Transcription levels in RPM and gene lengths of transcripts based on replication timing. (F) Single gene analysis of DNA replication levels from Fig 1C compared with the replication levels for the gene from the BrdU-Seq, calculated as reads per kilobase per million (RPKM) normalized to the Input, showing reproducible and consistent replication timing peak between the single gene and the BrdU-Seq analysis; n=3 for the BrdU IP, n=2 for the BrdU-Seq; average mean +/- SEM. (G) Nascent transcription levels presented as fold change (FC) relative to G1/S levels from Fig 1D; n=3; average mean +/- SEM; Student t-test. (H) Transcription activity measured by EU incorporation level followed by Clik-IT; asynchronous and serum starved released cells at the different timepoints are pulsed for 1h with EU before being fixed and analyzed by FACS. EU intensity is quantified for cells gated in G1 and G2 for DNA content and compared to EU incorporation levels in asynchronous growing cells. Average mean +/- Standard Deviation; Student t-test. (I) Procedure to derive reciprocal directionality between the leading replication fork and RNAPII transcription, with a specific example on chromosome (Chr) 7. DNA replication moves from replication origins to replication termination sites. Therefore, in a replication timing profile, the tip in the "inverted V" region (*Hansen et al., 2010*) will represent sites of replication initiation. Sites of replication termination will be instead where two consecutive "inverted V" converge in the replication timing profile. BrdU-Seq is not able to identify specifically DNA replication origins but the combination of all the timepoints together can identify regions where replication initiates. Highlighted in the figure as '1' '2' and '3' are three sites where replication initiates, numbered according to when these origins are activated along the S phase, from early to late. By knowing where the replication initiation sites are and as the leading replication fork progresses from 5' to 3' along the DNA, we can determine whether the leading replication fork is progressing along the Watson or the Crick strand across a specific region. By overlapping the directionality of the replication fork with that of the genes (from the TSS to the TTS), we can define the reciprocal directionality between the leading replication fork and the RNAPII. If both are moving in the same direction we define them as codirectional; if they move in opposite directions they are head to head; if they are in a combination of the two they are defined as transition, because of any of these i) the gene is replicated with replication forks entering from both ends; ii) a replication origin is activated inside the gene iii) the gene contains a replication termination site (see example of long gene in the bottom right, that contains both a replication origin and a replication termination site). Snapshot of the Chr-RNA-Seq data across the region is represented, to show that not all the transcripts present in the region are transcribed. (J) Frequency of codirectional, head to head and transition genes derived as from Fig S1I. (K) Transcription levels and gene lengths of genes in Fig S1J based on their directionality; COD = codirectional; HtH = head to head; Trans = transition; box-whiskers plots with line at the median, Mann-

Whitney t-test; test * => p-value < 0.05; ** => p-value < 0.01; *** => p-value < 0.001; **** => p-value < 0.0001.

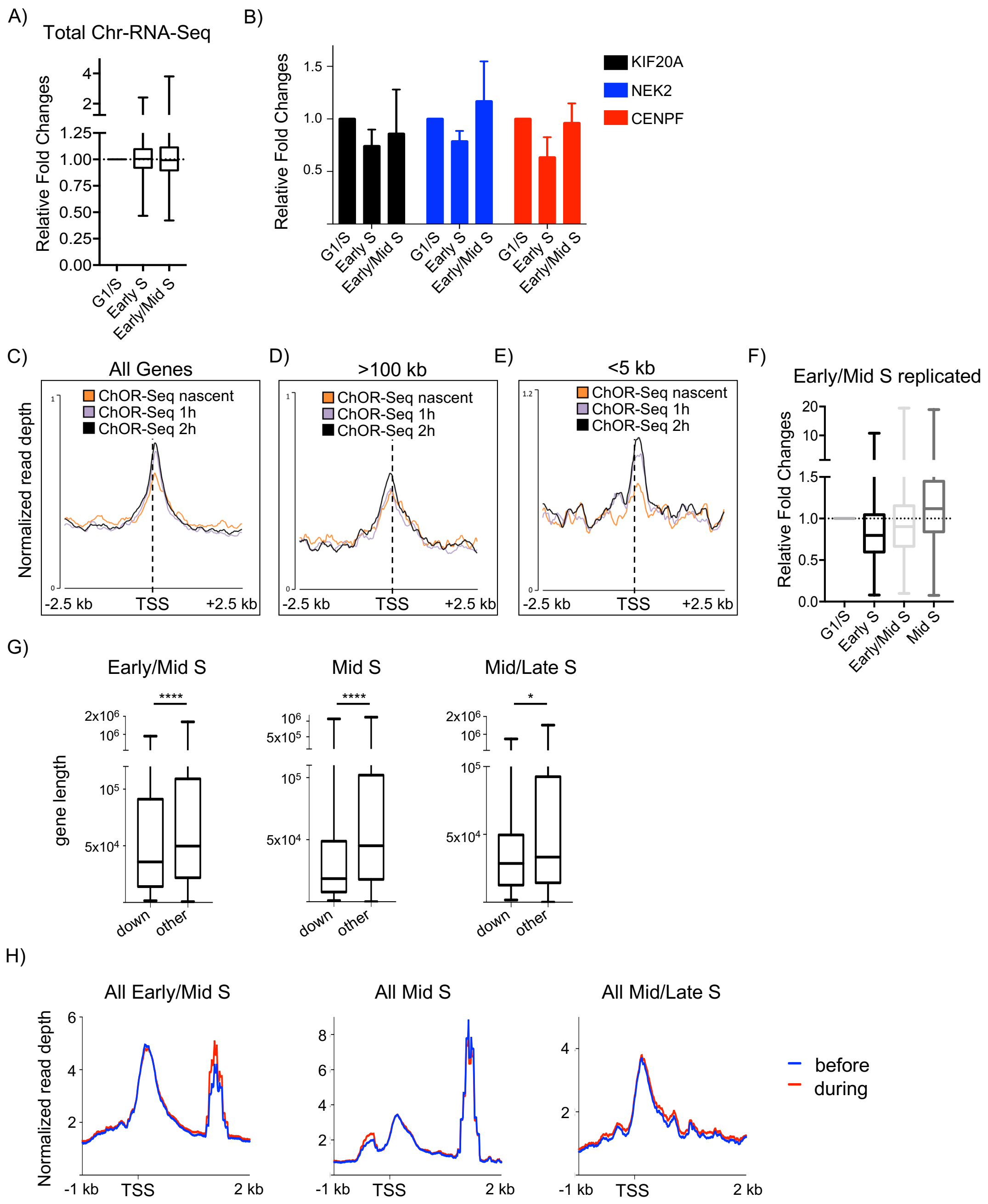

Figure S2. Transcription shut down of genes when they get replicated, Related to Figure 2

(A) As for Fig 2A, relative fold changes of Chr-RNA-Seq levels of Early S replicated genes compared to G1/S and Early/Mid S, over the whole gene including exons. (B) As for Fig 2B for genes replicated in Early S shown in Fig 1D and being more transcribed throughout S/G2. (C-E) ChOR-Seq profiles for Ser5-RNAPII from *Stewart-Morgan et al., 2019* in nascent replicated chromatin, 1 h and 2 h after replication, centered around the TSS +/- 2.5 kb for (C) All Genes in the mouse genome, (D) genes > 100 kb, and (E) genes < 5 kb. (F) As for Fig 2A but for the Early/Mid S replicated genes, showing broader reduced transcription shut down compared to G1/S. (G) Gene length for genes that show a reduction of nascent transcription levels when they get replicated compared to all the other transcripts replicated in the same timepoint; Mann-Whitney t-test; **** => p-value < 0.0001; * => p-value < 0.05. (H) As for Fig 2F for all the genes replicated in each timepoint, comparing average profile in the timepoint they are replicated (during) with the one earlier (before).

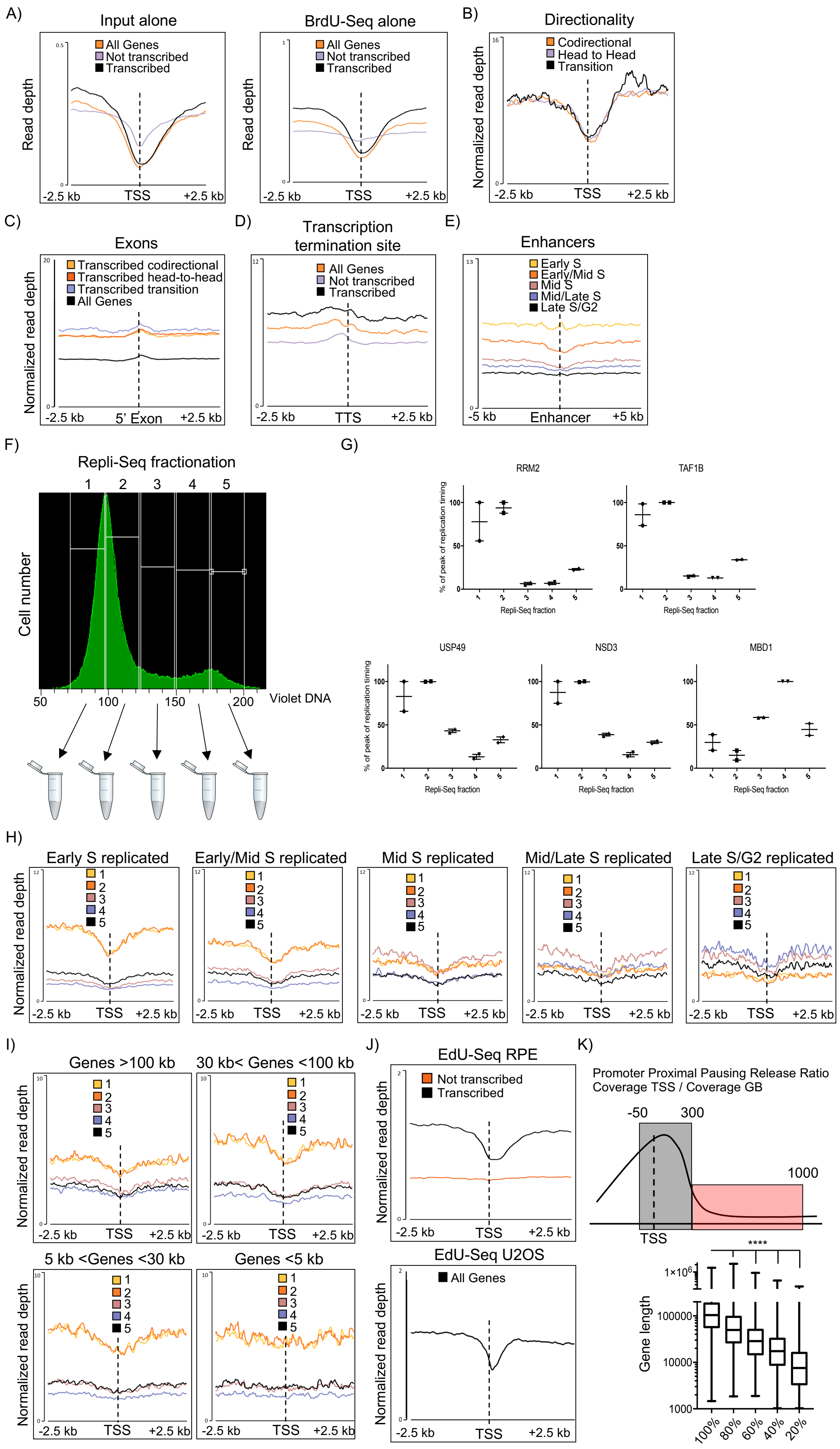
A)
Input alone
All Genes
Not transcribed
Transcribed
Read depth
-2.5 kb
TSS
+2.5 kb
BrdU-Seq alone
All Genes
Not transcribed
Transcribed
Read depth
-2.5 kb
TSS
+2.5 kb
B)
Directionality
Codirectional
Head to Head
Transition
Normalized read depth
-2.5 kb
TSS
+2.5 kb
C)
Exons
Transcribed codirectional
Transcribed head-to-head
Transcribed transition
All Genes
Normalized read depth
-2.5 kb
5' Exon
+2.5 kb
D)
Transcription termination site
All Genes
Not transcribed
Transcribed
-2.5 kb
TTS
+2.5 kb
E)
Enhancers
Early S
Early/Mid S
Mid S
Mid/Late S
Late S/G2
-5 kb
Enhancer
+5 kb
F)
Repli-Seq fractionation
1 2 3 4 5
Cell number
50 100 150 200
Violet DNA
G)
RRM2
TAF1B
USP49
NSD3
MBD1
% of peak of replication timing
Repli-Seq fraction
H)
Early S replicated
Early/Mid S replicated
Mid S replicated
Mid/Late S replicated
Late S/G2 replicated
1 2 3 4 5
Normalized read depth
-2.5 kb
TSS
+2.5 kb
I)
Genes >100 kb
30 kb< Genes <100 kb
5 kb <Genes <30 kb
Genes <5 kb
1 2 3 4 5
Normalized read depth
-2.5 kb
TSS
+2.5 kb
J)
EdU-Seq RPE
Not transcribed
Transcribed
EdU-Seq U2OS
All Genes
Normalized read depth
-2.5 kb
TSS
+2.5 kb
K)
Promoter Proximal Pausing Release Ratio
Coverage TSS / Coverage GB
-50
300
1000
TSS
****
Gene length
1×10⁶
100000
10000
1000
100%
80%
60%
40%
20%

Figure S3. TSS of transcribed genes are the only transcriptional pausing hotspots that affect globally DNA replication, Related to Figure 3

(A) As for Fig 3A but of Input and BrdU-Seq signals alone without normalization. (B) As for Fig 3A but with TSS of transcribed genes based on directionality according to Fig S1J. (C) Average metagene profile of BrdU-Seq levels of Early S normalized to Input DNA around the 5'-end of Exons +/- 2.5 kb from all genes and from transcribed genes separated based on their directionality according to Fig S1J. (D) Average metagene profile of BrdU-Seq levels of Early S normalized to Input DNA around the TTS +/- 2.5 kb of all genes, specifically transcribed genes or not transcribed genes. (E) Average metagene profile of BrdU-Seq levels normalized to Input DNA around enhancers mapped in RPE fibroblasts (*Cherry et al. 2018*) for all the timepoints. (F) An asynchronous population of BJ cells is pulsed for 30 min with BrdU before cells are sorted in 5 fractions based on their DNA content by Violet DNA. BrdU-Seq is then performed from each fraction and analyzed. (G) BrdU-Seq levels over the indicated genes in the 5 Repli-Seq fractions quantified as percentage of the fraction with the highest level; n=2; average mean +/- SEM. (H) As Fig 3A with BrdU levels across TSS +/- 2.5kb over genes replicated in the different timepoints from the 5 Repli-Seq fractions. (I) As Fig 3B of the BrdU-Seq level of the 5 Repli-Seq fractions over transcribed genes sorted by gene length. (J) EdU-Seq dataset reanalysis profile across transcribed and not transcribed genes in RPE fibroblasts and for All Genes in U2OS cells (*Macheret et al., 2018*). (K) Schematic on how P^3R^2 is computed, calculating the ratios of the GRO-Seq coverage from *Li et al., 2018* in the region -50/+300 around the TSS and +300/+1000 after the TSS, for all the transcribed genes longer than 1 kb. Gene length for genes according to their P^3R^2, with genes ranked from the highest to the lowest P^3R^2 and divided in 5 groups of equal size; box whiskers plots with line at the median, Mann-Whitney t-test; **** => p-value < 0.0001. Profiles generated using the computational environment EaSeq (*Lerdrup et al., 2016*).

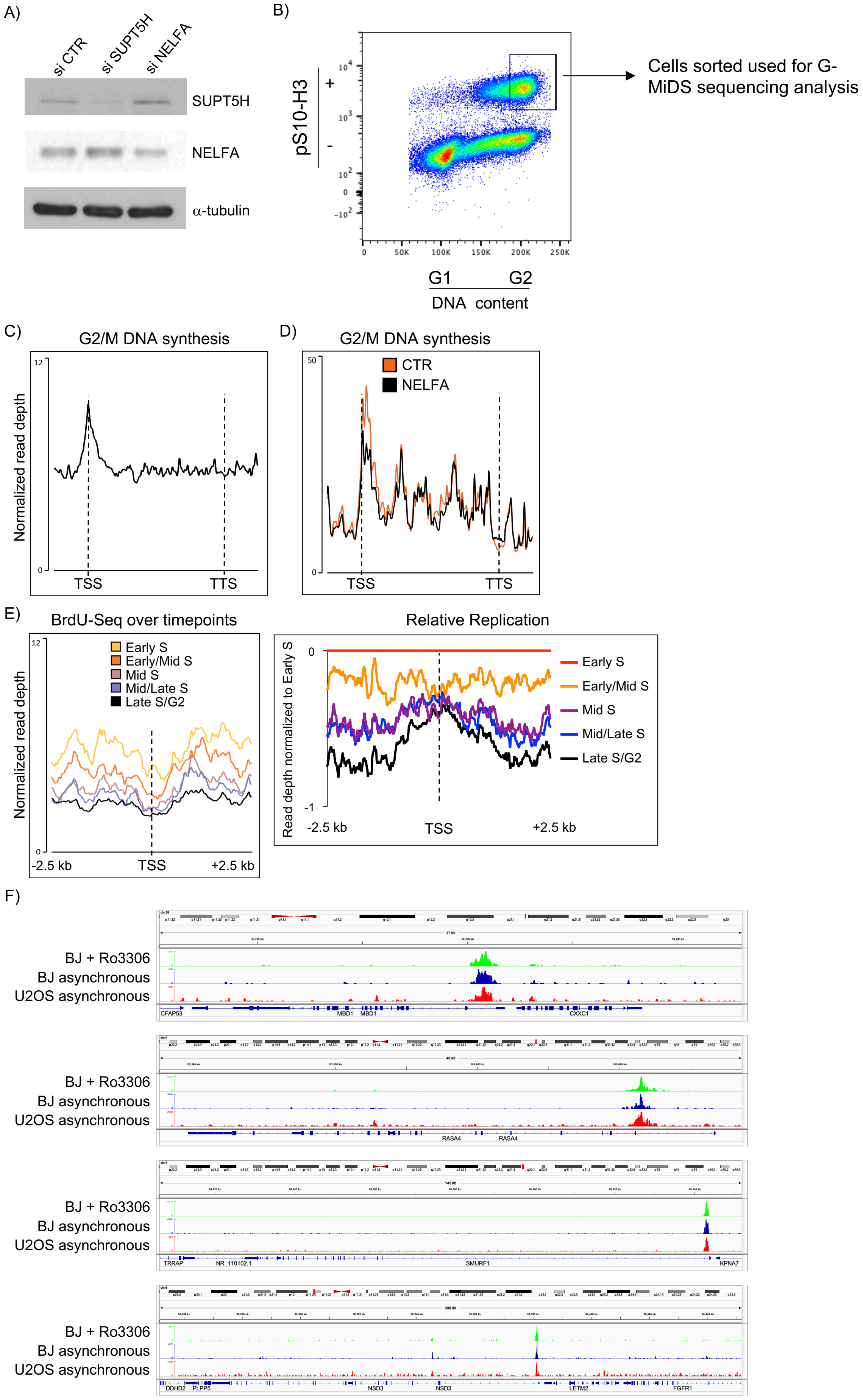
A)
si CTR
si SUPT5H
si NELFA
SUPT5H
NELFA
α-tubulin
B)
pS10-H3
+
-
G1
G2
DNA content
Cells sorted used for G-MiDS sequencing analysis
C)
G2/M DNA synthesis
Normalized read depth
TSS
TTS
D)
G2/M DNA synthesis
CTR
NELFA
TSS
TTS
E)
BrdU-Seq over timepoints
Early S
Early/Mid S
Mid S
Mid/Late S
Late S/G2
Normalized read depth
-2.5 kb
TSS
+2.5 kb
Relative Replication
Read depth normalized to Early S
Early S
Early/Mid S
Mid S
Mid/Late S
Late S/G2
-2.5 kb
TSS
+2.5 kb
F)
BJ + Ro3306
BJ asynchronous
U2OS asynchronous
BJ + Ro3306
BJ asynchronous
U2OS asynchronous
BJ + Ro3306
BJ asynchronous
U2OS asynchronous
BJ + Ro3306
BJ asynchronous
U2OS asynchronous

Figure S4. TSS of transcribed genes are sites of DNA synthesis in G2/M, Related to Figure 4

(A) Representative western blotting analysis of the siRNA of SUPT5H and NELFA in BJ cells with antibodies against SUPT5H and NELFA, α-tubulin is used as a loading control. (B) Representative image of the mitotic cells sorting strategy by FACS. Cells that are both in G2 by DNA content and pS10-H3 positive (highlighted in the box) are sorted followed by BrdU sequencing. (C) Average binned profile from TSS to TTS for G-MiDS specific BrdU-Seq across all transcribed genes. (D) Average binned profile from TSS to TTS for G-MiDS specific BrdU-Seq across G-MIDS hotspot genes upon for CTR siRNA and after siRNA of NELFA. (E) (left) Average metagene profile of BrdU-Seq levels normalized to Input DNA in all timepoints around the TSS +/- 2.5 kb for the 449 G-MiDS hotspot genes. (right) Relative enrichment for G-MIDS genes of BrdU-Seq levels of all the timepoints compared to the levels in Early S. (F) Snapshots with IGV of the G-MiDS sequencing files over the indicated G-MiDS hotspot TSS for BJ cells synchronized with Ro3306 (green), BJ cells in G2/M sorted from asynchronous cells (blue) and U2OS cells in G2/M sorted from asynchronous cells (red). Profiles generated using the computational environment EaSeq (*Lerdrup et al., 2016*).

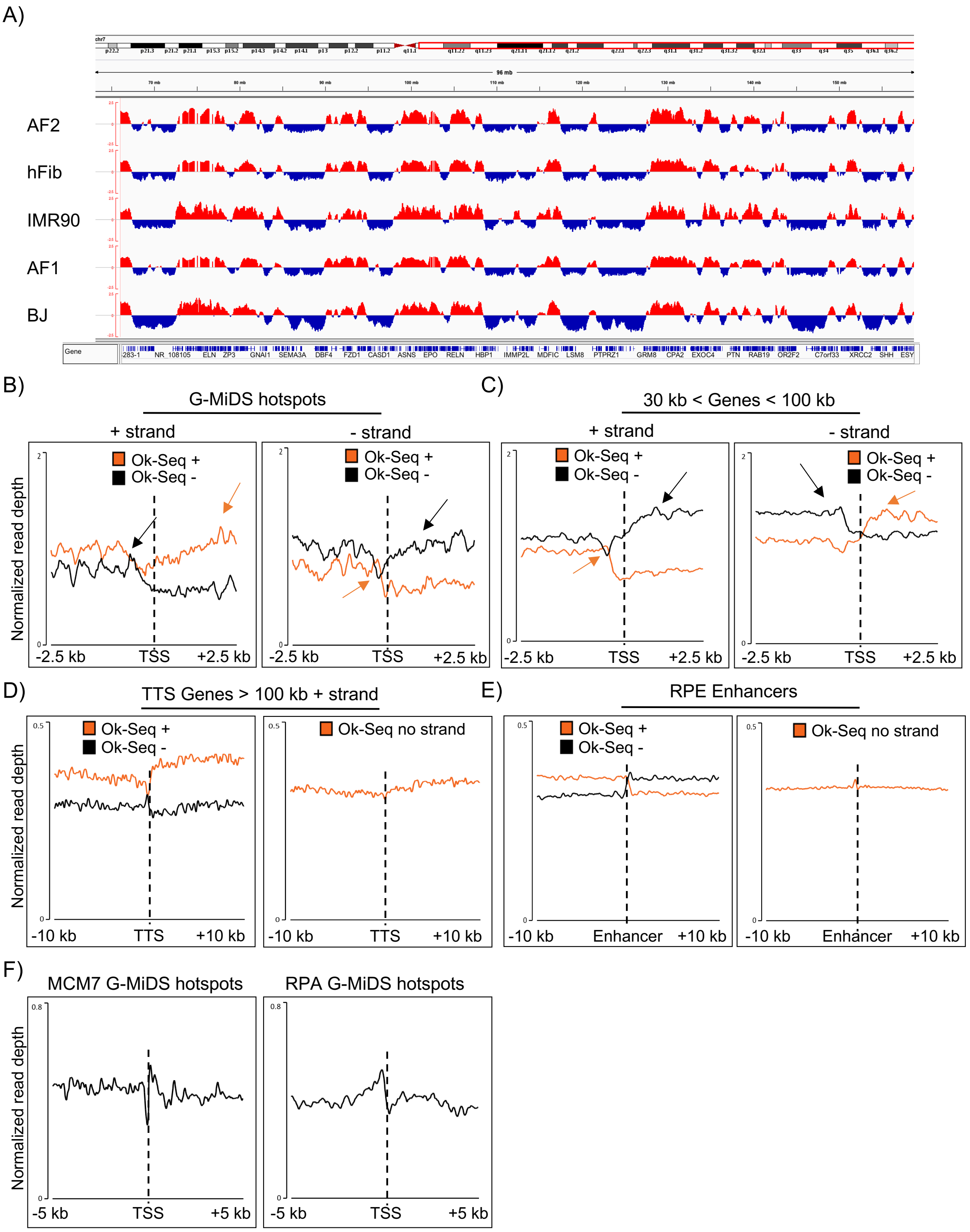

Figure S5 Asymmetric replication fork progression from TSS associated replication origins, Related to Figure 5

(A) IGV snapshot of Repli-Seq profiles from the indicated fibroblasts cell lines, showing how the replication program from early (red) to late (blue) S-phase is conserved among fibroblasts. Data derived from http://www.replicationdomain.com (*Weddington et al., 2008*). (B) Average metagene profile for strand specific Ok-Seq from *Chen et al., 2019* +/- 2.5 kb around the TSS of G-MiDS hotspot genes, either on the '+' or the '-' strand. (C) As for (B) but specifically +/- 2.5 kb around the TSS of transcribed genes > 30 kb and < 100 kb in

BJ-hTERT. (D) Average metagene profile for strand specific Ok-Seq from *Chen et al., 2019* +/- 10 kb around the TTS of transcribed genes > 100 kb in BJ-hTERT, either on the '+' or the '-' strand (left panel), and with no strand specificity (right panel). (E) As for (D) +/- 10kb around enhancers identified in RPE cells as from *Cherry et al., 2018*. (F) As for Fig 5E specifically across the G-MiDS hotspots. Profiles generated using the computational environment EaSeq (*Lerdrup et al., 2016*).

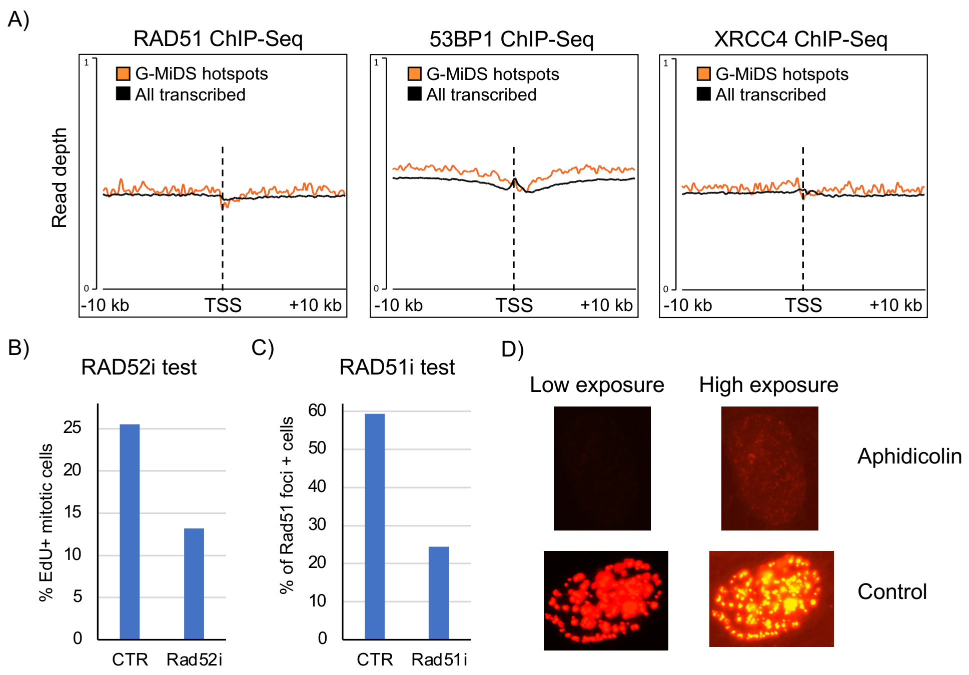

Figure S6 DNA damage levels associated with G-MiDS, Related to Figure 6

(A) Read depth for the ChIP-Seq of RAD51, 53BP1 and XRCC4 in U2OS cells at the TSS +/- 10kb of all transcribed genes and only G-MiDS hotspots from from *Clouaire et al. 2018.* (B) Quantification of EdU foci positive mitotic cells following treatment for 16 h with 0.4 µM aphidicolin and Ro3306, released from the G2 arrest in medium containing EdU for 30 minutes in the presence of DMSO (CTR) or of 20 µM of Rad52 inhibitor (Rad52i). (C) Quantification of Rad51 foci positive cells in cells treated for 24 h with 1 µM of the PARP inhibitor Olaparib, treated in parallel either with DMSO (CTR) or with 25 µM Rad51 inhibitor (Rad51i). (D) Comparison between EdU intensity of G2/M cells in cells untreated, or cells treated with 10 µM aphidicolin once released from the Ro3306 arrest; normal 'Low exposure' or equally amplified 'High exposure'.

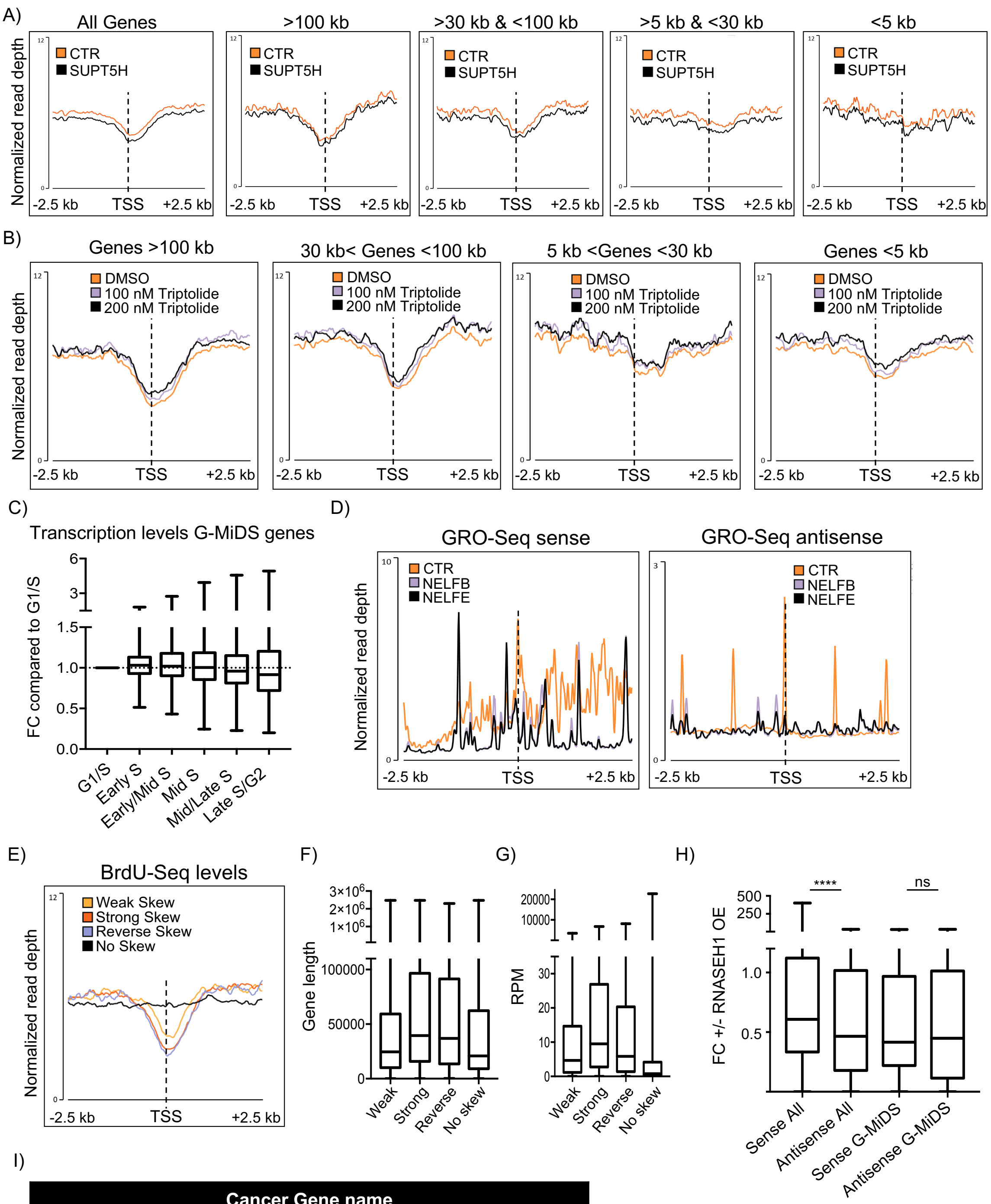

I)

Cancer Gene name			
ARHGAP5	CTNND1	FGFR1OP	RECQL4
ARHGEF10	CUX1	HOXD11	RUNX1T1
CANT1	DICER1	KAT6B	SEPTIN9
CHEK2	DNM2	MAP2K2	TAF15
CIC	EBF1	MECOM	TRIM33
COL3A1	ELN	NCOR2	
CREB3L2	FBLN2	PDE4DIP	

Figure S7. BrdU gaps are dependent more generally on transcription and not only RNAPII; general features of G-MiDS hotspot genes and overlap with Cosmic Cancer Genes, Related to Figure 7

(A) Average metagene profile of BrdU-Seq levels of Early S normalized to Input DNA around the TSS +/- 2.5 kb for all transcribed genes and transcripts separated by gene length in CTR siRNA and after the siRNA of SUPT5H. (B) Average metagene profile of BrdU-Seq levels of Early S normalized to Input DNA around the TSS +/- 2.5 kb in DMSO and cells treated for 1 h with the indicated concentrations of triptolide while being pulsed with BrdU; plots are for transcribed genes separated by gene length. (C) Fold changes in the transcription levels of G-MiDS hotspot genes compared to the G1/S Chr-RNA-Seq timepoint throughout all other timepoints. (D) Average metagene profile of GRO-Seq levels around the TSS +/- 2.5kb of all genes in the human genomes for sense and antisense transcription, in control (CTR) cells and cells after the RNAi of NELFB and NELFE; data derived from *Liu et al., 2017*. (E) Average metagene profile of BrdU-Seq levels of Early S normalized to Input DNA of genes according to promoter class based on GC richness and GC skew as defined in *Ginno et al., 2013*. (F-G) (F) Gene length and (G) and transcription levels for genes based on their promoter class as defined in *Ginno et al., 2013*. (H) Quantification of Cap-Seq levels from *Tan-Wong et al., 2019* at the TSS +/- 1 kb in a strand specific manner across ALL the transcribed genes in BJ-hTERT cells and only the G-MiDS hotspots, as fold change (FC) after the overexpression (OE) of RNASEH1. Only genes with a quantification for both sense and antisense transcription have been analyzed; Mann-Whitney t-test; **** => p-value < 0.0001, ns = not significative. (I) List of the G-MiDS hotspot genes identified in the COSMIC database as Cancer Genes, in particular genes with copy number alterations and/or sites of translocations.

Table S1: Gene ontology analysis of transcripts transcribed in the first time point at the G1/S transition, using the online DAVID Bioinformatic Resources (https://david.ncifcrf.gov), with DNA replication highlighted in bold. Related to Figure 1

GO Term	***Counts***	***p-Value***	***Benjamini***
transcription, DNA-templated	1413	1.50E-42	1.40E-38
cell division	304	2.00E-31	9.60E-28
cell-cell adhesion	244	9.80E-31	3.10E-27
rRNA processing	198	6.90E-29	1.60E-25
mitotic nuclear division	221	2.20E-26	4.10E-23
DNA repair	211	3.00E-26	4.70E-23
mRNA splicing, via spliceosome	200	2.30E-25	3.20E-22
proteasome-mediated ubiquitin-dependent protein catabolic process	183	2.50E-23	3.00E-20
protein transport	322	4.20E-23	4.40E-20
regulation of transcription, DNA-templated	1051	1.10E-22	1.00E-19
cellular response to DNA damage stimulus	185	5.70E-22	4.90E-19
translation	218	9.50E-22	7.50E-19
**DNA replication**	**144**	**1.50E-21**	**1.10E-18**

Table S2: Gene ontology analysis of transcripts changing +/-2 Fold Change expression compared to the first time point at the G1/S transition, using the online DAVID Bioinformatic Resources (https://david.ncifcrf.gov). Related to Figure 1.

Term	Count	%	P-Value	Benjamini	
nucleosome assembly	7	15.2	1.10E-07	2.60E-05	**16h UP**
telomere organization	4	8.7	2.00E-05	2.40E-03	52 transcripts
DNA replication-dependent nucleosome assembly	4	8.7	3.30E-05	2.70E-03	
chromatin silencing at rDNA	4	8.7	5.10E-05	3.10E-03	
protein heterotetramerization	4	8.7	7.50E-05	3.70E-03	
negative regulation of gene expression, epigenetic	4	8.7	1.30E-04	5.20E-03	
positive regulation of gene expression, epigenetic	4	8.7	2.40E-04	8.40E-03	
gene silencing by RNA	4	8.7	1.30E-03	4.00E-02	
cellular protein metabolic process	4	8.7	1.60E-03	4.20E-02	
mitochondrial electron transport, NADH to ubiquinone	3	6.5	4.20E-03	9.70E-02	
nucleosome assembly	25	18.9	8.70E-30	5.70E-27	**19h UP**
telomere organization	14	10.6	2.80E-22	9.30E-20	147 transcripts
DNA replication-dependent nucleosome assembly	14	10.6	4.80E-21	1.00E-18	
chromatin silencing at rDNA	14	10.6	4.70E-20	7.80E-18	
protein heterotetramerization	14	10.6	3.30E-19	4.40E-17	
negative regulation of gene expression, epigenetic	14	10.6	4.40E-18	4.80E-16	
positive regulation of gene expression, epigenetic	14	10.6	9.60E-17	1.00E-14	
gene silencing by RNA	14	10.6	2.70E-13	2.30E-11	
cellular protein metabolic process	14	10.6	6.10E-13	4.50E-11	
regulation of gene silencing	7	5.3	2.80E-11	1.80E-09	
chromatin silencing	9	6.8	4.00E-10	2.40E-08	
negative regulation of megakaryocyte differentiation	7	5.3	1.10E-09	5.90E-08	
telomere capping	7	5.3	5.70E-09	2.90E-07	
DNA replication-independent nucleosome assembly	7	5.3	1.30E-08	6.00E-07	
DNA-templated transcription, initiation	7	5.3	1.00E-07	4.50E-06	
double-strand break repair via nonhomologous end joining	8	6.1	1.50E-07	6.40E-06	
CENP-A containing nucleosome assembly	7	5.3	3.10E-07	1.20E-05	
beta-catenin-TCF complex assembly	7	5.3	3.10E-07	1.20E-05	
innate immune response in mucosa	5	3.8	1.80E-05	6.80E-04	
blood coagulation	9	6.8	2.50E-05	8.60E-04	
mitotic nuclear division	10	7.6	3.20E-05	1.00E-03	
antibacterial humoral response	5	3.8	1.80E-04	5.70E-03	
defense response to Gram-positive bacterium	6	4.5	2.20E-04	6.50E-03	
cell division	10	7.6	4.30E-04	1.20E-02	
cell division	42	17	3.60E-29	3.10E-26	**22h UP**
nucleosome assembly	28	11.3	2.50E-27	1.10E-24	299 transcripts
mitotic nuclear division	33	13.4	3.80E-24	1.10E-21	
telomere organization	14	5.7	1.10E-18	2.20E-16	
DNA replication-dependent nucleosome assembly	14	5.7	1.70E-17	2.90E-15	
chromatin silencing at rDNA	14	5.7	1.70E-16	3.10E-14	
negative regulation of gene expression, epigenetic	15	6.1	4.30E-16	5.40E-14	
protein heterotetramerization	14	5.7	1.10E-15	1.20E-13	
chromosome segregation	16	6.5	2.00E-15	1.90E-13	
sister chromatid cohesion	18	7.3	4.20E-15	3.60E-13	
positive regulation of gene expression, epigenetic	14	5.7	3.10E-13	2.40E-11	
gene silencing by RNA	14	5.7	6.70E-10	4.70E-08	
regulation of gene silencing	7	2.8	1.10E-09	7.30E-08	
cellular protein metabolic process	14	5.7	1.40E-09	8.70E-08	
CENP-A containing nucleosome assembly	10	4	1.50E-09	8.60E-08	
chromatin silencing	10	4	2.40E-09	1.20E-07	
mitotic sister chromatid segregation	8	3.2	1.20E-08	5.90E-07	
negative regulation of megakaryocyte differentiation	7	2.8	4.20E-08	2.00E-06	
metaphase plate congression	6	2.4	1.60E-07	7.30E-06	
telomere capping	7	2.8	2.20E-07	9.30E-06	
anaphase-promoting complex-dependent catabolic process	10	4	3.90E-07	1.60E-05	
DNA replication-independent nucleosome assembly	7	2.8	4.90E-07	1.90E-05	
regulation of attachment of spindle microtubules to kinetochore	5	2	6.50E-07	2.40E-05	
spindle organization	6	2.4	8.70E-07	3.10E-05	
attachment of spindle microtubules to kinetochore	5	2	2.30E-06	7.80E-05	
positive regulation of ubiquitin protein ligase activity	5	2	2.30E-06	7.80E-05	
DNA-templated transcription, initiation	7	2.8	3.70E-06	1.20E-04	
mitotic metaphase plate congression	7	2.8	4.40E-06	1.40E-04	
microtubule-based movement	9	3.6	5.20E-06	1.60E-04	
G2/M transition of mitotic cell cycle	11	4.5	5.40E-06	1.60E-04	
double-strand break repair via nonhomologous end joining	8	3.2	9.30E-06	2.60E-04	
innate immune response in mucosa	6	2.4	9.70E-06	2.70E-04	
beta-catenin-TCF complex assembly	7	2.8	1.10E-05	2.90E-04	
mitotic cytokinesis	6	2.4	2.10E-05	5.40E-04	
mitotic spindle organization	6	2.4	2.50E-05	6.20E-04	
regulation of chromosome segregation	4	1.6	8.80E-05	2.10E-03	
regulation of mitotic spindle organization	4	1.6	8.80E-05	2.10E-03	
mitotic spindle midzone assembly	4	1.6	8.80E-05	2.10E-03	
regulation of cell cycle	9	3.6	1.20E-04	2.70E-03	
cell proliferation	15	6.1	1.20E-04	2.80E-03	

regulation of ubiquitin-protein ligase activity involved in mitotic cell cycle	5	2	1.40E-04	3.10E-03	
antibacterial humoral response	6	2.4	1.70E-04	3.60E-03	
regulation of mitotic nuclear division	5	2	1.70E-04	3.60E-03	
cytokinesis	6	2.4	2.50E-04	5.20E-03	
protein ubiquitination involved in ubiquitin-dependent protein catabolic process	9	3.6	4.80E-04	9.70E-03	
defense response to Gram-positive bacterium	7	2.8	5.20E-04	1.00E-02	
positive regulation of cytokinesis	5	2	9.30E-04	1.80E-02	
regulation of meiotic nuclear division	3	1.2	1.40E-03	2.50E-02	
free ubiquitin chain polymerization	3	1.2	1.40E-03	2.50E-02	
negative regulation of ubiquitin-protein ligase activity involved in mitotic cell cycle	6	2.4	1.50E-03	2.80E-02	
mitotic spindle assembly checkpoint	4	1.6	1.60E-03	2.90E-02	
homologous chromosome segregation	3	1.2	2.00E-03	3.50E-02	
positive regulation of ubiquitin-protein ligase activity involved in regulation of mitotic cell cycle transition	6	2.4	2.10E-03	3.50E-02	
retrograde vesicle-mediated transport, Golgi to ER	6	2.4	2.90E-03	4.80E-02	
attachment of mitotic spindle microtubules to kinetochore	3	1.2	3.70E-03	6.00E-02	
cell cycle	9	3.6	4.40E-03	6.90E-02	
antigen processing and presentation of exogenous peptide antigen via MHC class II	6	2.4	4.80E-03	7.30E-02	
regulation of mitotic metaphase/anaphase transition	3	1.2	4.80E-03	7.20E-02	
protein localization to kinetochore	3	1.2	5.90E-03	8.70E-02	
microtubule depolymerization	3	1.2	5.90E-03	8.70E-02	
DNA damage response, signal transduction by p53 class mediator resulting in cell cycle arrest	5	2	6.20E-03	9.00E-02	
blood coagulation	8	3.2	6.40E-03	9.10E-02	
cell division	49	15.3	3.90E-32	4.50E-29	**24h UP**
nucleosome assembly	32	10	7.20E-30	4.10E-27	382 transcripts
mitotic nuclear division	40	12.5	1.90E-28	7.30E-26	
telomere organization	15	4.7	4.00E-19	1.20E-16	
DNA replication-dependent nucleosome assembly	15	4.7	8.80E-18	2.00E-15	
sister chromatid cohesion	21	6.5	6.40E-17	2.10E-14	
chromatin silencing at rDNA	15	4.7	1.10E-16	1.80E-14	
negative regulation of gene expression, epigenetic	16	5	4.70E-16	6.30E-14	
protein heterotetramerization	15	4.7	8.50E-16	1.10E-13	
chromosome segregation	17	5.3	3.60E-15	4.10E-13	
positive regulation of gene expression, epigenetic	15	4.7	3.70E-13	3.80E-11	
gene silencing by RNA	16	5	1.30E-10	1.20E-08	
mitotic cytokinesis	10	3.1	2.90E-10	2.50E-08	
CENP-A containing nucleosome assembly	11	3.4	6.80E-10	5.50E-08	
G2/M transition of mitotic cell cycle	16	5	2.60E-09	2.00E-07	
cellular protein metabolic process	15	4.7	3.10E-09	2.20E-07	
negative regulation of megakaryocyte differentiation	8	2.5	4.70E-09	3.20E-07	
regulation of gene silencing	7	2.2	5.00E-09	3.20E-07	
chromatin silencing	10	3.1	2.10E-08	1.20E-06	
anaphase-promoting complex-dependent catabolic process	12	3.7	2.90E-08	1.70E-06	
telomere capping	8	2.5	3.40E-08	1.90E-06	
microtubule-based movement	12	3.7	3.80E-08	2.00E-06	
innate immune response in mucosa	8	2.5	6.50E-08	3.20E-06	
mitotic sister chromatid segregation	8	2.5	6.50E-08	3.20E-06	
mitotic metaphase plate congression	9	2.8	6.70E-08	3.20E-06	
spindle organization	7	2.2	8.20E-08	3.70E-06	
DNA replication-independent nucleosome assembly	8	2.5	8.80E-08	3.90E-06	
beta-catenin-TCF complex assembly	9	2.8	2.30E-07	9.80E-06	
mitotic spindle organization	8	2.5	2.60E-07	1.10E-05	
metaphase plate congression	6	1.9	5.60E-07	2.20E-05	
DNA-templated transcription, initiation	8	2.5	9.80E-07	3.70E-05	
regulation of attachment of spindle microtubules to kinetochore	5	1.6	1.80E-06	6.50E-05	
antibacterial humoral response	8	2.5	4.10E-06	1.50E-04	
double-strand break repair via nonhomologous end joining	9	2.8	4.80E-06	1.70E-04	
defense response to Gram-positive bacterium	10	3.1	5.60E-06	1.90E-04	
positive regulation of ubiquitin protein ligase activity	5	1.6	6.20E-06	2.00E-04	
cytokinesis	8	2.5	7.40E-06	2.40E-04	
protein localization to kinetochore	5	1.6	1.00E-05	3.10E-04	
regulation of ubiquitin-protein ligase activity involved in mitotic cell cycle	6	1.9	2.10E-05	6.30E-04	
regulation of mitotic nuclear division	6	1.9	2.60E-05	7.60E-04	
cell division	61	8.1	2.70E-24	6.30E-21	**28h UP**
mitotic nuclear division	50	6.6	8.50E-23	1.00E-19	874 transcripts
nucleosome assembly	33	4.4	1.60E-19	1.30E-16	
telomere organization	15	2	7.50E-14	4.40E-11	
sister chromatid cohesion	25	3.3	2.00E-13	9.50E-11	
DNA replication-dependent nucleosome assembly	15	2	1.50E-12	5.90E-10	
protein heterotetramerization	16	2.1	8.10E-12	2.70E-09	
negative regulation of gene expression, epigenetic	17	2.3	1.10E-11	3.30E-09	
chromatin silencing at rDNA	15	2	1.60E-11	4.30E-09	
cellular protein metabolic process	24	3.2	3.30E-11	7.80E-09	
chromosome segregation	18	2.4	2.00E-10	4.40E-08	
G2/M transition of mitotic cell cycle	24	3.2	7.50E-10	1.50E-07	
positive regulation of gene expression, epigenetic	15	2	3.30E-08	6.10E-06	

CENP-A containing nucleosome assembly	12	1.6	2.60E-07	4.30E-05	
mitotic cytokinesis	10	1.3	5.30E-07	8.30E-05	
spindle organization	8	1.1	6.80E-07	1.00E-04	
anaphase-promoting complex-dependent catabolic process	15	2	8.10E-07	1.10E-04	
regulation of gene silencing	7	0.9	8.70E-07	1.10E-04	
retrograde vesicle-mediated transport, Golgi to ER	15	2	1.30E-06	1.60E-04	
metaphase plate congression	7	0.9	1.70E-06	2.00E-04	
mitotic sister chromatid segregation	9	1.2	1.80E-06	2.00E-04	
negative regulation of megakaryocyte differentiation	8	1.1	1.80E-06	1.90E-04	
antibacterial humoral response	11	1.5	2.90E-06	3.00E-04	
chromatin silencing	11	1.5	3.60E-06	3.60E-04	
mitotic metaphase plate congression	10	1.3	5.10E-06	4.80E-04	
gene silencing by RNA	16	2.1	1.10E-05	1.00E-03	
regulation of ubiquitin-protein ligase activity involved in mitotic cell cycle	8	1.1	1.20E-05	1.00E-03	
telomere capping	8	1.1	1.20E-05	1.00E-03	
innate immune response in mucosa	8	1.1	2.10E-05	1.80E-03	
DNA replication-independent nucleosome assembly	8	1.1	2.80E-05	2.30E-03	
negative regulation of ubiquitin-protein ligase activity in mitotic cell cycle	12	1.6	4.60E-05	3.60E-03	
defense response to Gram-positive bacterium	13	1.7	5.40E-05	4.10E-03	
regulation of attachment of spindle microtubules to kinetochore	5	0.7	5.50E-05	4.00E-03	
mitotic spindle organization	8	1.1	7.80E-05	5.60E-03	
positive regulation of ubiquitin-protein ligase activity involved in regulation of mitotic cell cycle transition	12	1.6	8.60E-05	6.00E-03	
beta-catenin-TCF complex assembly	9	1.2	1.30E-04	9.00E-03	
microtubule-based movement	12	1.6	1.60E-04	1.00E-02	
regulation of mitotic nuclear division	7	0.9	1.70E-04	1.10E-02	
positive regulation of ubiquitin protein ligase activity	5	0.7	1.90E-04	1.10E-02	
DNA-templated transcription, initiation	8	1.1	2.60E-04	1.60E-02	
protein localization to kinetochore	5	0.7	3.00E-04	1.80E-02	
protein K11-linked ubiquitination	7	0.9	3.40E-04	1.90E-02	
mitochondrial ATP synthesis coupled proton transport	6	0.8	7.70E-04	4.20E-02	
positive regulation of T cell mediated cytotoxicity	5	0.7	9.40E-04	5.00E-02	
negative regulation of cell proliferation	28	3.7	1.30E-03	6.70E-02	
cytokinesis	8	1.1	1.60E-03	8.10E-02	
mitotic chromosome condensation	5	0.7	1.70E-03	8.30E-02	
DNA damage response, signal transduction by p53 class mediator resulting in cell cycle arrest	9	1.2	1.70E-03	8.20E-02	
cell redox homeostasis	10	1.3	1.80E-03	8.60E-02	
cell proliferation	26	3.4	1.90E-03	8.60E-02	
double-strand break repair via nonhomologous end joining	9	1.2	1.90E-03	8.60E-02	
type I interferon signaling pathway	9	1.2	2.10E-03	9.30E-02	
positive regulation of I-kappaB kinase/NF-kappaB signaling	15	2	2.20E-03	9.50E-02	
regulation of chromosome segregation	4	0.5	2.30E-03	9.80E-02	
mitotic spindle midzone assembly	4	0.5	2.30E-03	9.80E-02	
					**16h DOWN**
No GO Term enrichment					13 transcripts
negative regulation of signal transduction	3	6.8	2.50E-03	5.50E-01	**19h DOWN**
Peyer's patch development	2	4.5	1.10E-02	8.20E-01	49 transcripts
negative regulation of oligodendrocyte differentiation	2	4.5	2.40E-02	9.20E-01	
olfactory bulb development	2	4.5	4.10E-02	9.60E-01	
DNA replication	3	6.8	4.60E-02	9.50E-01	
regulation of cell proliferation	3	6.8	6.30E-02	9.70E-01	
DNA replication initiation	2	4.5	6.80E-02	9.60E-01	
DNA replication initiation	6	5	9.30E-07	6.40E-04	**22h DOWN**
G1/S transition of mitotic cell cycle	8	6.6	1.80E-06	6.10E-04	138 transcripts
DNA replication	8	6.6	2.80E-05	6.40E-03	
negative regulation of Notch signaling pathway	4	3.3	5.80E-04	9.40E-02	
negative regulation of oligodendrocyte differentiation	3	2.5	1.70E-03	2.10E-01	
DNA replication initiation	13	8.8	2.70E-18	2.40E-15	**24h DOWN**
DNA replication	20	13.6	2.90E-18	1.30E-15	166 transcripts
G1/S transition of mitotic cell cycle	17	11.6	2.70E-17	8.00E-15	
regulation of transcription involved in G1/S transition of mitotic cell cycle	7	4.8	1.20E-08	2.70E-06	
DNA replication checkpoint	4	2.7	2.10E-05	3.70E-03	
DNA unwinding involved in DNA replication	4	2.7	4.40E-05	6.50E-03	
DNA replication	33	6.8	3.50E-21	6.40E-18	**28h DOWN**
DNA replication initiation	16	3.3	1.40E-16	1.00E-13	537 transcripts
G1/S transition of mitotic cell cycle	23	4.8	2.50E-15	1.50E-12	
telomere maintenance via recombination	12	2.5	1.00E-10	4.80E-08	
DNA strand elongation involved in DNA replication	7	1.5	7.50E-07	2.80E-04	
DNA synthesis involved in DNA repair	9	1.9	1.30E-06	4.10E-04	
regulation of transcription involved in G1/S transition of mitotic cell cycle	7	1.5	1.30E-05	3.40E-03	
DNA repair	19	3.9	1.50E-05	3.40E-03	
DNA replication checkpoint	5	1	2.10E-05	4.30E-03	
DNA unwinding involved in DNA replication	5	1	6.00E-05	1.10E-02	
DNA duplex unwinding	8	1.7	7.50E-05	1.30E-02	
intracellular signal transduction	24	5	1.10E-04	1.70E-02	
nucleotide-excision repair, DNA gap filling	6	1.2	2.20E-04	3.10E-02	
negative regulation of Notch signaling pathway	6	1.2	5.60E-04	7.20E-02	
folic acid metabolic process	5	1	7.50E-04	8.90E-02	

Table S3: Correlation analysis between the BrdU-Seq and Chr-RNA-Seq time points and repeats. Related to Figure 1.

Correlation between timepoints based on RPM for each gene > 1RPM

	14h	16h	19h	22h	24h
**14h**	1	0.991	0.99	0.978	0.974
**16h**		1	0.988	0.96	0.959
**19h**			1	0.988	0.988
**22h**				1	0.998
**24h**					1

14h = G1/S
16h = Early S
19h = Early/Mid S
22h = Mid S
24h = Mid/Late S
28h = Late S/G2

Correlations between biological repeats based on RPM for each gene >1 RPM

	14h	16h	19h	22h	24h
**14h**	0.987				
**16h**		0.992			
**19h**			0.992		
**22h**				0.996	
**24h**					0.994

Table S4: Gene ontology analysis of G-MiDS hotspot transcripts, using the online DAVID Bioinformatic Resources (https://david.ncifcrf.gov). Related to Figure 7.
/

GO Term	*Counts*	*p-Value*	*Benjamini*
transcription, DNA-templated/	75/	1.30E-06/	1.90E-03/
regulation of transcription, DNA-templated/	62/	1.50E-06/	1.10E-03/
synaptic transmission, glutamatergic/	5/	9.90E-04/	4.00E-01/
positive regulation of protein phosphorylation/	9/	6.70E-03/	9.30E-01/
endocytic recycling/	4/	1.50E-02/	9.90E-01/
fatty acid beta-oxidation/	5/	1.50E-02/	9.80E-01/
positive regulation of transcription, DNA-templated/	20/	1.80E-02/	9.80E-01/
protein targeting to plasma membrane/	4/	1.80E-02/	9.70E-01/
peptidyl-tyrosine phosphorylation/	9/	1.90E-02/	9.60E-01/
regulation of defence response to virus by virus/	4/	2.20E-02/	9.70E-01/
positive regulation of GTPase activity/	21/	2.30E-02/	9.60E-01/
ER to Golgi vesicle-mediated transport/	9/	2.40E-02/	9.60E-01/
regulation of small GTPase mediated signal transduction/	8/	2.80E-02/	9.60E-01/
cell migration/	9/	3.50E-02/	9.80E-01/
positive regulation of protein kinase B signalling/	6/	3.60E-02/	9.80E-01/

/
