# Supplementary material for: Persistence of RNA transcription during DNA replication delays duplication of transcription start sites until G2/M
Source: Cell Rep. 2021 Feb 16;34(7):108759. doi: 10.1016/j.celrep.2021.108759 (PMC7900609; doi:10.1016/j.celrep.2021.108759)
Supplement: Document S2. Article plus supplemental information [file mmc2.pdf]

Cell Reports

Article

Persistence of RNA transcription during DNA replication delays duplication of transcription start sites until G2/M

Graphical Abstract

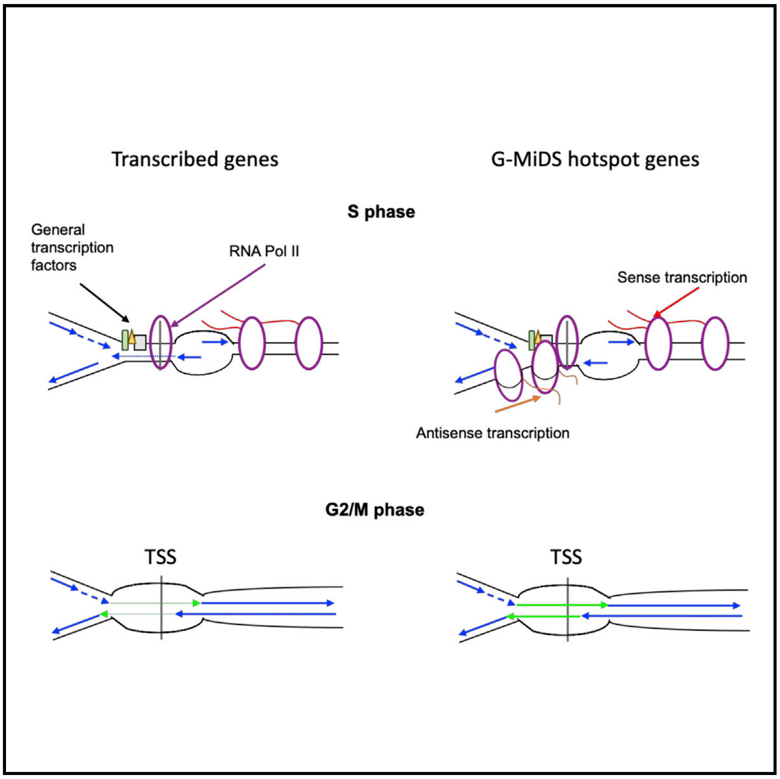

Highlights

- During DNA replication, RNA polymerase II remains active at transcription start sites
- Transcription start sites of hundreds of genes are duplicated only in G2/M
- G2/M DNA synthesis (G-MiDS) is not associated with DNA damage repair processes
- G-MiDS is separated from mitotic DNA synthesis (MiDAS)

Authors

Jianming Wang, Patricia Rojas, Jingwen Mao, ..., Martin R. Higgs, Paloma Garcia, Marco Saponaro

Correspondence

m.saponaro@bham.ac.uk

In Brief

Wang et al. show how transcription and replication affect each other in human cells. They find that transcription start sites are not fully replicated together with their neighboring regions and observe hundreds of cases in which duplication is completed only when cells are entering mitosis.

Wang et al., 2021, Cell Reports *34*, 108759
February 16, 2021 © 2021 The Author(s).
https://doi.org/10.1016/j.celrep.2021.108759

CellPress

Cell Reports

CellPress
OPEN ACCESS

Article

Persistence of RNA transcription during DNA replication delays duplication of transcription start sites until G2/M

Jianming Wang,[1,4] Patricia Rojas,[1,4] Jingwen Mao,[1] Martina Mustè Sadurnì,[1] Olivia Garnier,[1] Songshu Xiao,[1]
Martin R. Higgs,[2] Paloma Garcia,[3] and Marco Saponaro[1,5,*]
[1]Transcription Associated Genome Instability Laboratory, Institute of Cancer and Genomic Sciences, University of Birmingham, Birmingham B15 2TT, UK
[2]Lysine Methylation and DNA Damage Laboratory, Institute of Cancer and Genomic Sciences, University of Birmingham, Birmingham B15 2TT, UK
[3]Stem Cells and Genome Stability Laboratory, Institute of Cancer and Genomic Sciences, University of Birmingham, Birmingham B15 2TT, UK
[4]These authors contributed equally
[5]Lead contact
*Correspondence: m.saponaro@bham.ac.uk
https://doi.org/10.1016/j.celrep.2021.108759

SUMMARY

As transcription and replication use DNA as substrate, conflicts between transcription and replication can occur, leading to genome instability with direct consequences for human health. To determine how the two processes are coordinated throughout S phase, we characterize both processes together at high resolution. We find that transcription occurs during DNA replication, with transcription start sites (TSSs) not fully replicated along with surrounding regions and remaining under-replicated until late in the cell cycle. TSSs undergo completion of DNA replication specifically when cells enter mitosis, when RNA polymerase II is removed. Intriguingly, G2/M DNA synthesis occurs at high frequency in unperturbed cell culture, but it is not associated with increased DNA damage and is fundamentally separated from mitotic DNA synthesis. TSSs duplicated in G2/M are characterized by a series of specific features, including high levels of antisense transcription, making them difficult to duplicate during S phase.

INTRODUCTION

A large body of evidence has established that RNA transcription can impair DNA replication progression, inducing replication stress and DNA damage (Brewer and Fangman, 1988; Deshpande and Newlon, 1996; Huertas and Aguilera, 2003; Helmrich et al., 2011; Alzu et al., 2012; Akamatsu and Kobayashi, 2015). Defective transcription caused by deregulation of transcription factors correlates with even greater DNA damage levels (Gaillard and Aguilera, 2016; Hamperl and Cimprich, 2016). Indeed, transcription factors are identified as major drivers of genome instability in human cells (Paulsen et al., 2009). Transcription-associated genome instability is often linked to a specific transcriptional defect, including (1) formation or persistence of RNA-DNA hybrids (so-called R-loops; Huertas and Aguilera, 2003), (2) increased topological constraints (Bermejo et al., 2009; Tuduri et al., 2009), and (3) accumulation of stalled/paused RNA polymerase (Dutta et al., 2011; Saponaro et al., 2014). To avoid head-to-head collisions between the transcription and replication machineries, bacterial genomes have transcription of major genes in a codirectional orientation with the replication fork emanating from the replication origin (Brewer, 1988). However, such organization is not possible in higher eukaryotes because of the inherent variability of cell-type-specific transcription and replication programs (Hansen et al., 2010). One possible solution to avoid conflicts would be to keep the two processes separated spatially (different parts of the genome used by the two processes) or temporally (different moments when a cell transcribes from when it replicates). Regarding the spatial separation, previous studies examining the nuclear distribution of transcription and replication sites have shown contrasting results, with some showing overlap between transcription and DNA synthesis (Hassan et al., 1994) and others showing spatial separation (Wansink et al., 1994; Wei et al., 1998). Regarding temporal separation, while DNA replication occurs during S phase, transcription can occur in any cell-cycle stage (Liang et al., 2015). In particular, RNA polymerase II (RNAPII), which is responsible for the transcription of protein-coding genes and the majority of the genome (Djebali et al., 2012), transcribes replication machinery factors and histones specifically during S phase (van der Meijden et al., 2002). Moreover, replication timing and distribution of replication origins are affected by RNAPII transcription, with early-replicated regions and replication origins enriched around transcribed genes (Cayrou et al., 2015; Petryk et al., 2016). Nevertheless, it is unclear how this reciprocal relationship is arranged in mammalian cells, despite its importance for genome stability and direct links to several human diseases (Gaillard and Aguilera, 2016).

This is an open access article under the CC BY license (http://creativecommons.org/licenses/by/4.0/).

A

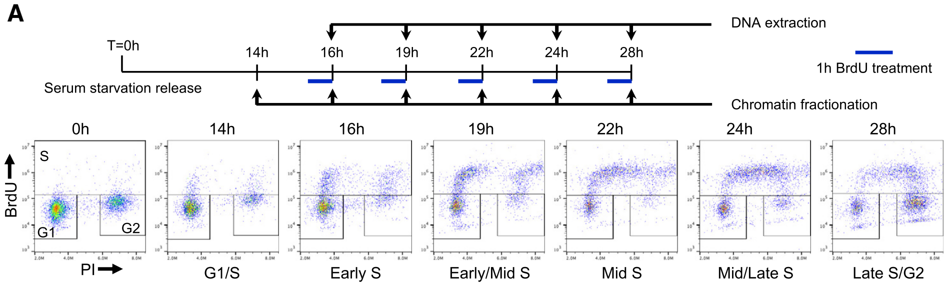

	G0/G1	G1/S	Early S	Early/Mid S	Mid S	Mid/Late S	Late S/G2
**G1**	68.3 ± 0.4	65.1 ± 1.1	52.0 ± 2.0	39.3 ± 1.2	27.4 ± 0.9	27.2 ± 1.0	21.5 ± 1.3
**S**	5.3 ± 1.0	17.2 ± 2.5	37.3 ± 4.0	52.1 ± 2.0	67.7 ± 1.5	61.0 ± 1.9	37.3 ± 1.6
**G2**	25.4 ± 0.7	16.7 ± 1.2	8.6 ± 1.2	7.7 ± 1.1	4.4 ± 0.7	10.8 ± 1.0	40.0 ± 1.4

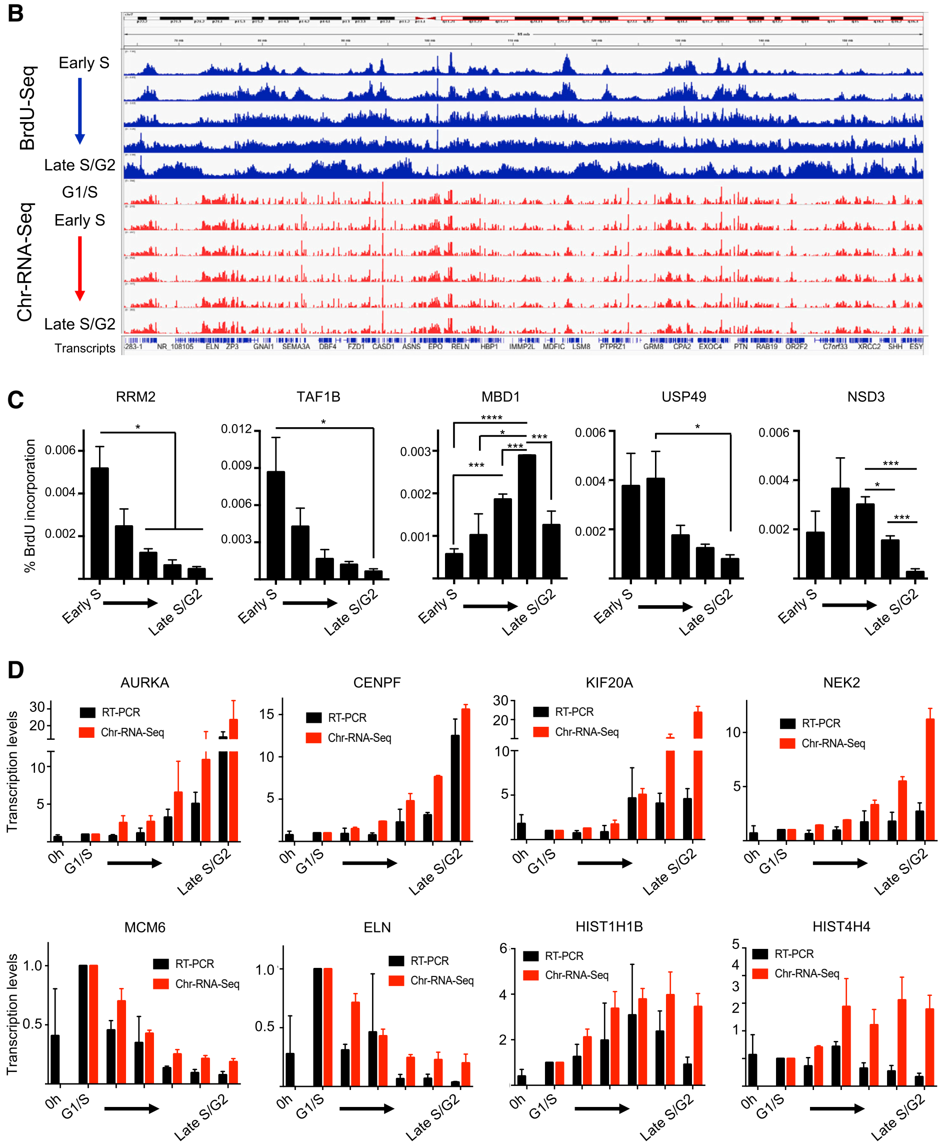

(legend on next page)

For this reason, we set out to characterize both transcription and replication throughout S phase with a combination of genomic approaches. We uncovered that (1) replication of a transcribed gene leads to a transient shut down in transcription activity; (2) replication of transcription start sites (TSSs) is delayed compared to nearby regions; (3) DNA synthesis across TSSs is completed when cells prepare for mitosis in 20% of cells; (4) G2/M DNA synthesis is not coinciding with sites of DNA damage, does not require canonical DNA damage repair pathways, and is different from mitotic DNA synthesis; and (5) TSSs that undergo G2/M DNA synthesis are conserved among different cell lines and characterized among other features by high levels of TSS-associated antisense transcription. Our findings provide an insight into how DNA replication and RNAPII transcription affect one another, with important consequences for completion of the DNA replication program and genome stability maintenance.

RESULTS

Genomic approaches to characterize transcription and replication during S phase

Previously, several groups analyzed whether sites of transcription and replication overlapped by immunofluorescence, presenting conflicting results (Wansink et al., 1994; Hassan et al., 1994; Wei et al., 1998). As these assays were not able to assess whether transcription and replication could affect each other, we decided to investigate the coordination between transcription and replication using a combination of genome-wide approaches. Human immortalized fibroblasts (BJ-hTERT) were synchronized by serum starvation for 26 h in G0/G1, reentering cell cycle once released in complete medium (Figure 1A). To monitor entry of cells in S phase, we analyzed the incorporation of the nucleotide analog bromodeoxyuridine (BrdU) as a proxy for DNA synthesis by fluorescence-activated cell sorting (FACS). Cells started to enter S phase 14 h post-release, as evidenced by increased incorporation of BrdU by FACS, corresponding to the G1/S transition point. Entry and progression of cells through the S phase was highly reproducible, with nearly 70% of cells in S phase by the mid-S time point (Figure 1A).

To characterize DNA replication specifically in each time point, we pulsed cells with BrdU exclusively for 1 h before the time point (Figure 1A). Pull-down of BrdU-labeled DNA followed by next-generation sequencing identified replicated DNA within each time point. Combining the BrdU-seq time points together as previously described (Rausch et al., 2018) we obtained a replication timing profile from early to late S phase (Figure S1A). Our synchronization and release strategy was highly reproducible, as shown by FACS analysis (Figure 1A), overlap of the replication timing profiles for two biological replicates (Figure S1A), and similarity with previously published Repli-seq profiles (Figure S1B).

To analyze transcription activity, we fractionate the BrdU-labeled cells to purify the chromatin fraction that contains transcriptionally engaged phosphorylated serine 5 RNAPII (hereafter Ser5-RNAPII; Figure S1C). We then extracted RNA from this fraction and performed RNA sequencing (RNA-seq) of chromatin-bound RNA (hereafter Chr-RNA-seq). Gene Ontology analysis of the Chr-RNA-seq at the G1/S time point identified 13,473 transcripts with a read per million (RPM) ≥ 1, with cells primed for replication indicated by the transcription of DNA replication factors (van der Meijden et al., 2002) (p value = 1.1E-18; Table S1). We used this time point as reference in our analysis, investigating changes in transcription activity during S phase for 12,425 transcripts with RPM ≥ 1 in all time points. Interestingly, we found that overall transcription activity was not radically affected throughout S phase, with mainly cell-cycle-regulated genes changing transcription levels (Table S2; Chr-RNA-seq replicates correlations; Table S3). A snapshot of the long arm of chromosome 7 is presented in Figure 1B, showing together BrdU-seq in blue and Chr-RNA-seq in red.

We determined when replication passes through a transcribed gene as the time point with the highest BrdU level over such gene. Combining all datasets together, we confirmed that transcribed genes were preferentially replicated early (Hansen et al., 2010), as >70% of all active transcripts were replicated by the early/mid-S time point, and only 6.5% replicated in the last time point (Figure S1D). Overall, early-S-phase replicated genes were also more transcribed and shorter (Figure S1E).

We validated our experimental approach by single-gene analysis for both DNA replication (BrdU-IP [BrdU-immunoprecipitation]) and pre-mRNA levels in independent repeats. For the BrdU-IP, we selected two genes previously shown to be early replicated in BJ-hTERT cells (Marchal et al., 2018) and another three whose replication peaked in other time points from our genomic analysis. We could prove significant differences in DNA replication levels across these genes in different time points, paralleling the DNA replication levels obtained quantifying BrdU-seq levels over such genes (Figures 1C and S1F). For transcription activity, we assessed nascent transcription levels by RT-PCR with primers designed across intron-exon junctions for the genes with the greatest fold changes throughout our kinetic plus two histone genes specifically transcribed in S phase. Also in this case, the single-gene analysis showed significant changes in transcription activity between time points, similar to the fold changes measured by Chr-RNA-seq (Figures 1D and S1G). A small proportion of cells is delayed for their cell-cycle entry (top left corner of late S/G2 in Figure 1A); hence, we assessed the transcription contribution of these cells. We

Figure 1. A system for analyzing transcription-replication coordination

(A) Experimental design schematic and FACS analysis of propidium iodide (PI) and BrdU to monitor S-phase progression, with quantification of cells number in each box (bottom left, G0/G1 phase; top, S phase; bottom right, G2 phase; n = 3).

(B) Representative genomic view of BrdU-seq (in blue) and Chr-RNA-seq (in red) on the long arm of chromosome 7, with a 95-Mb view at all time points.

(C) Single-gene analysis of BrdU incorporation levels as percentage of the input at each time point over the indicated genes; n = 3.

(D) Nascent transcription levels as relative ratio to G1/S compared to the fold changes measured by Chr-RNA-seq; n = 3 for RT-PCR, n = 2 for Chr-RNA-seq. Data represent mean ± SEM. Student's t test; *p < 0.05; **p < 0.01; ***p < 0.001; ****p < 0.0001.

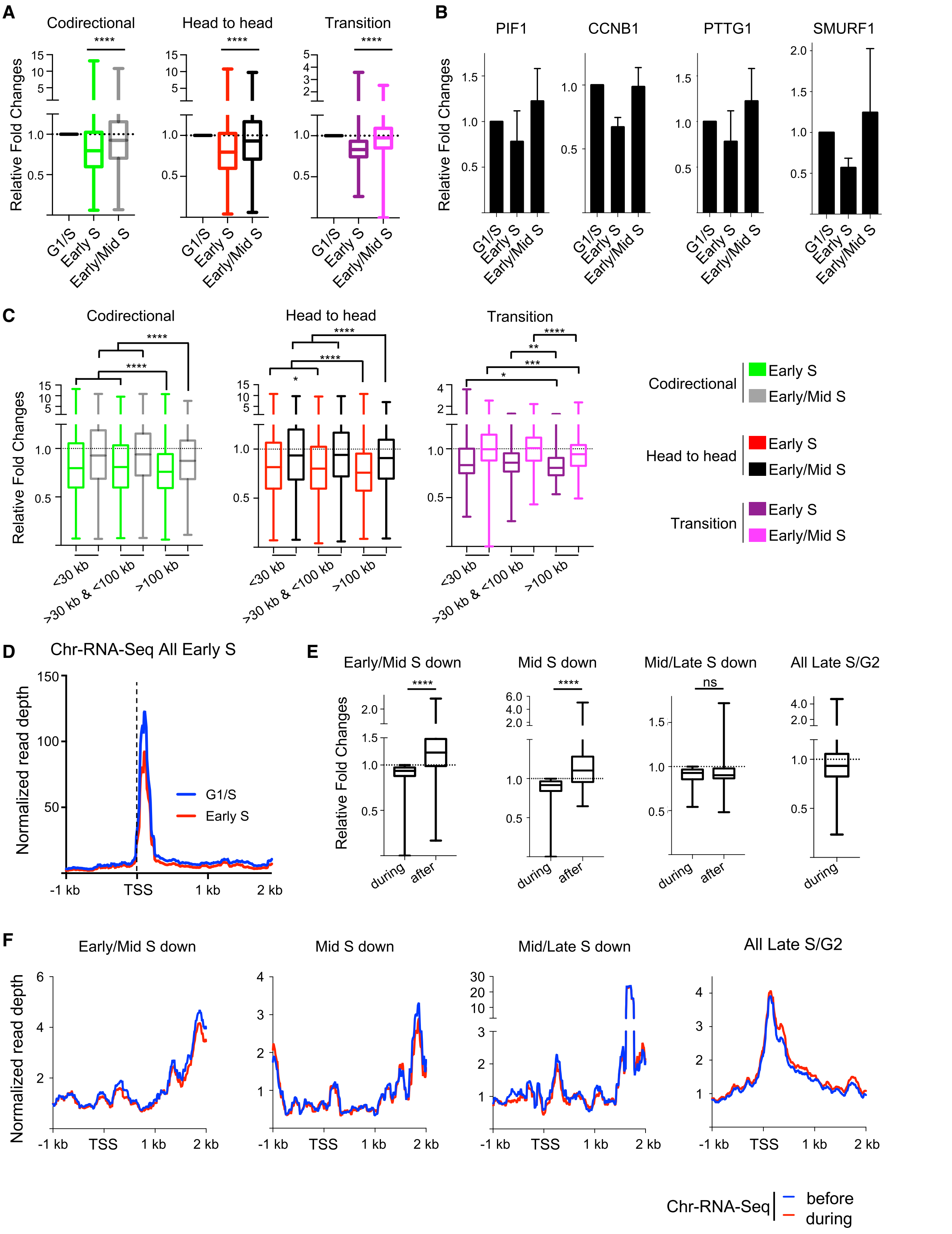

(legend on next page)

monitored transcription activity by incorporation of the ribonucleotide analog ethynyluridine (EU) followed by Click-iT reaction by FACS in all time points compared to asynchronous cells. When analyzing EU incorporation specifically in G0/G1 cells by DNA content, we found that in all time points EU incorporation is significantly lower compared to an asynchronous population of cells (Figure S1H). On the contrary, when analyzing cells in G2 by DNA content, we found that once cells reenter the cell cycle and reach G2, transcription activity is the same of an untreated population of cells (Figure S1H). This is further supported by the fact that transcription levels for MCM6 and ELN are 13- and 27-fold reduced compared to the G1/S time point (Figures 1D and S1G).

Finally, based on the replication timing, we determined whether the leading fork was progressing along the Watson or the Crick strand of the DNA; leading replication forks move from peaks in the replication timing profile (indicating replication initiation regions) to troughs (indicating replication termination regions; Figure S1I). As gene transcription occurs on the Watson or the Crick strand, from TSSs to transcription termination sites (TTSs) (Figure S1I), we were able to define the reciprocal directionality of transcription and leading replication fork progression, determining whether these were in a codirectional (transcription and replication moving in the same direction) or a head-to-head (transcription and replication moving toward each other) conformation. Analyzing the frequency of the directionality of transcribed genes with the leading-strand replication fork, we found the same frequency in genes being codirectional (42.18%) or head to head (42.85%). The remaining transcripts were defined as “transition” (14.96%), as not uniquely replicated across in one direction, hence with a transition point in DNA replication direction (Figure S1J). This transition could be due to either the activation of replication origins or the presence of replication termination sites. Transition genes were significantly longer than those in the other groups (Figure S1K), in agreement with previous data whereby long genes often contain an origin of replication downstream the TSS and a replication termination site inside the gene body (Pourkarimi et al., 2016; Chen et al., 2019). When comparing transcription levels of genes based on their directionality, we found no significant difference between codirectional and head-to-head genes (Figure S1K) overall or at specific time points (data not shown). Similarly, the directionality between transcribed genes and DNA replication did not change throughout S phase (data not shown).

Having established that our experimental setup is sensitive and reproducible, we investigated how transcription and replication impact each other during S phase.

Transient shutdown of nascent transcription when genes are replicated

Although our Chr-RNA-seq showed no radical changes in transcription during S phase, we analyzed specifically how DNA replication of a gene affects its transcription activity. To achieve this, we monitored nascent transcription quantifying Chr-RNA-seq levels only over introns, as exonic signal could be derived from mature mRNA still bound to the chromatin (Tilgner et al., 2012). We first analyzed the first time point, observing on average a 20% reduction in transcription activity specifically when genes are replicated, independently of directionality (Figure 2A). This transcription shutdown was almost completely recovered by the following time point (Figure 2A). Importantly, transcription shutdown was not detectable if we quantified Chr-RNA-seq over the whole gene, indicating that the shutdown was detectable only when assessing nascent transcription (Figure S2A). By analyzing nascent transcription over single genes, we confirmed that when genes are replicated, there is a transient reduction in transcription activity (Figure 2B), even for genes that increase their transcription throughout S/G2 (Figure S2B). When reanalyzing these data clustered by gene length, we found that independently of directionality, longer genes presented a slightly more pronounced shutdown and delayed recovery in transcription activity than shorter genes (Figure 2C). Furthermore, by analyzing the average Chr-RNA-seq profile around the TSS, we found that transcription was still active when early-S genes were replicated, explaining the quick recovery (Figure 2D). In agreement, rapid recovery of transcription after replication has been previously reported in mouse embryonic stem cells (Stewart-Morgan et al., 2019). Moreover, when we reanalyzed ChOR-seq (Ser5-RNAPII chromatin occupancy after replication) data from Stewart-Morgan et al. (2019), we found that RNAPII persisted at TSSs in nascent synthesized DNA, more on genes >100 kb than those <5 kb (Figures S2C–S2E).

For the later time points, we identified the genes that showed reduced transcription activity when replicated, generally recovering transcription activity by the following time point (Figure 2E). These genes were enriched for shorter transcripts within those replicated in each time point (Figure S2G), suggesting that replication of longer genes may take longer than one time point. We could also detect a reduction of transcription activity for all genes replicated in the late-S/G2 time point compared to the previous one (Figure 2E). Importantly, despite the dip in transcription activity, at all time points, these genes showed persistent levels of transcription around the TSS region (Figures 2F and S2H).

Taken together, these data demonstrate that DNA replication has a transient impact on transcription only when genes get

Figure 2. Transient transcription shutdown when genes are replicated

(A) Quantification of Chr-RNA-seq levels over introns at the indicated time points, as fold change to G1/S. Genes are separated based on directionality as in Figure S1J.

(B) Nascent transcription levels in early S and early/mid-S compared to G1/S as in Figure 1D for genes replicated in early S; n = 3, data represent mean ± SEM.

(C) As in (A), with genes clustered by length.

(D) Average metagene profile of Chr-RNA-seq in G1/S and early S around the TSS −1 kb/+2 kb of genes replicated in early S.

(E) Fold change in Chr-RNA-seq levels for genes with reduced nascent transcription when replicated compared to the time point before, with levels during and after replication. The analysis includes 514 genes in early/mid-S, 144 genes in mid-S, 178 genes in mid-/late S, and all 716 genes in late S/G2; paired t test analysis.

(F) As in (D) but specifically over the genes highlighted in (E).

Box whiskers plots with line at the median, Mann-Whitney t test; ns, not significant; *p < 0.05; **p < 0.01; ***p < 0.001; ****p < 0.0001.

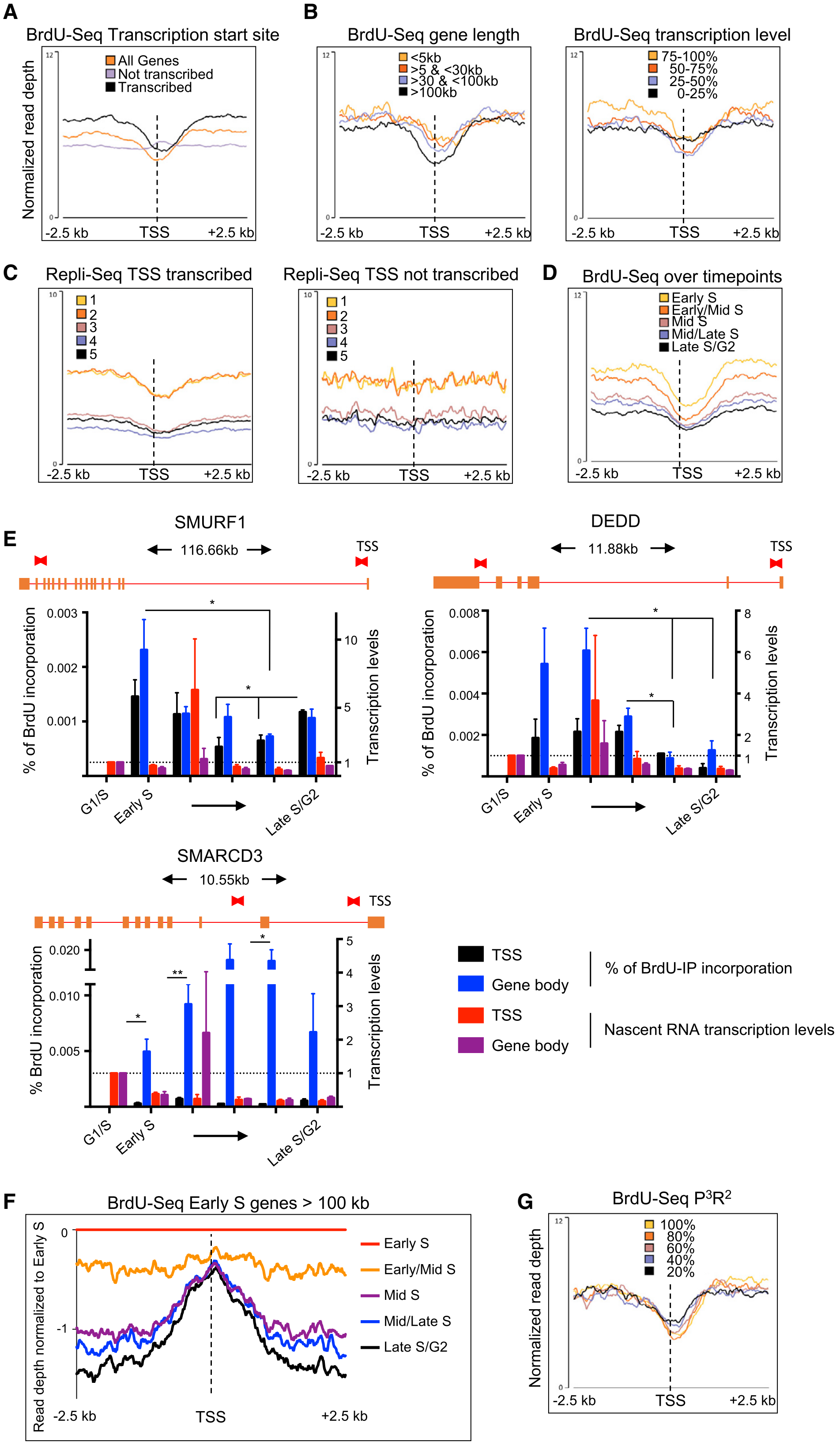

(legend on next page)

replicated throughout S phase. We hypothesize that this is due to maintenance of RNAPII near the TSS also during replication to restart transcription once replication has traversed (Figures 2D and 2F).

Paucity of DNA replication around TSSs of transcribed genes

Since our data highlighted that transcription persists while genes were replicated, we analyzed how this impacted DNA replication. Strikingly, we found that TSSs of transcribed genes exhibited decreased levels of BrdU signal, suggesting that they were under-replicated (Figure 3A). This was specifically due to a decrease in BrdU levels and independent of directionality (Figures S3A and S3B). Other transcription pausing sites, such as TTSs and exons, showed no visible effect on the BrdU-seq profile, irrespective of directionality, while enhancers showed only a very small decreased BrdU signal in the early/mid-S and mid-S time points (Figures S3C–S3E and data not shown). The size of this gap correlated with gene lengths, with longer transcripts correlating with larger and deeper gaps than shorter genes, but transcription levels did not affect gap size, except for the lowest transcribed genes (Figure 3B). To test whether gaps were a consequence of our synchronization strategy, we performed a Repli-seq experiment. Exponentially growing cells were pulsed with BrdU for 30 min and sorted in five fractions according to DNA content (Figure S3F). We validated that Repli-seq fractions recapitulate replication timings identified from our single-gene analysis of Figure 1C (Figure S3G). Importantly, also analyzing the Repli-seq samples, we found a gap across the TSSs of transcribed genes in all fractions (Figure 3C), correlating with timing of replication of the transcribed genes from our Chr-RNA-seq and gene length (Figures S3H and S3I). Equally, we could identify the presence of under-replicated DNA at TSSs in EdU-seq datasets from RPE and U2OS cells released from mitotic shake-off (Macheret and Halazonetis, 2018; Figure S3J).

The size of this under-replicated gap at TSSs was approximately 2 kb on average and overlapped with sites of accumulation of transcription evidenced by Chr-RNA-seq (Figure 2D). Importantly, gaps that formed at the TSS of genes >100 kb replicated in early S persisted over all other time points (Figure 3D). We could verify that the rate of BrdU incorporation over single genes was lower around the TSS when compared with the gene body (compare black bars with blue ones in Figure 3E), even though transcription was relatively stable at both positions throughout the kinetic (compare red and purple bars in Figure 3E). In the SMURF1 gene replicated in early S, we detected a significant increase in DNA synthesis specifically across the TSS at the late-S/G2 time point (Figure 3E). Therefore, we analyzed at TSSs of genes >100 kb replicated in early S the relative enrichment of DNA synthesis of the later time points compared to early S, detecting an increase of DNA synthesis specifically at TSSs at the later time points (Figure 3F). Together, these findings suggest that DNA replication across the TSS is less efficient and delayed compared to the rest of the gene.

Next, we investigated whether RNAPII presence was the cause of the BrdU gaps at TSSs. Persistence of the RNAPII near TSS regions is controlled by promoter proximal pausing (PPP; Rahl et al., 2010). To assess how much the RNAPII is promoter proximal paused at TSSs, we calculated the level of PPP release ratio (P^3R^2; Figure S3K) using previously published GRO-seq data for BJ-hTERT (Li et al., 2018). We found that transcripts with greater P^3R^2 levels (i.e., more RNAPII on the TSS than in the gene body, indicative of more PPP) tended to have also larger and deeper gaps than those with lower P^3R^2 (Figure 3G), correlating with longer genes being also more promoter proximal paused (Figure S3K).

When combined with the findings above, these data indicate that persistence of RNAPII during gene replication near the TSS correlates with a concomitant under-replication of these sites.

Duplication of TSSs requires G2/M DNA synthesis

Our data highlight that DNA replication across TSSs is postponed compared to nearby regions, showing some evidence of DNA synthesis in late-S/G2 phase. DNA synthesis can still occur up until mitosis and is enhanced following DNA replication stress (Minocherhomji et al., 2015; Maya-Mendoza et al., 2018). In parallel, RNAPII is largely removed from the chromatin in G2/M when cells enter mitosis (Liang et al., 2015). Hence, we hypothesized that some of the sites where G2/M DNA synthesis (hereafter G-MiDS) occurs may well be TSSs. We postulated that altering RNAPII levels at the TSS might affect G-MiDS levels. To test this, we analyzed the proportion of G2/M cells (positive for phospho-Ser10 H3, hereafter pS10-H3) that still underwent DNA synthesis, monitored by the incorporation of the nucleotide analog ethynyldeoxyuridine (EdU) followed by Click-iT reaction (Figure 4A). Cells were synchronized in G2 with the CDK1 inhibitor Ro3306 for 16 h following our standard synchronization strategy, released in EdU for 30 min to label sites of DNA synthesis, and immunofluorescenced with pS10-H3 to identify G2/M cells. In parallel, we depleted two factors crucial for PPP, NELFA and SUPT5H, part of the negative elongation factor (NELF) and DRB-sensitivity-inducing factor (DSIF) complexes (Figure S4A). RNAi

Figure 3. TSSs of transcribed genes remain under-replicated throughout S phase

(A) Average metagene profile of BrdU-seq levels normalized to input DNA in early S at TSSs ±2.5 kb for all genes, transcribed genes, or not transcribed genes.

(B) As for (A) for genes replicated in early S according to Figure S1D, separated in four groups by gene length or transcription levels.

(C) As for (A) for transcribed and not transcribed genes in all five fractions of the Repli-seq.

(D) As for (A) for genes >100 kb replicated in early S in all time points.

(E) BrdU incorporation at TSSs and in the gene body for the indicated genes in all time points. Schematic of gene structure with gene length, exons as orange boxes, and introns as red lines; primer positions are specified by the red boxes above; nascent transcription levels at TSSs and gene body are shown as fold change compared to G1/S for SMURF1 first time points shown in Figure 2B; n = 3, data represent mean ± SEM, Student's t test.

(F) Relative enrichment of BrdU-seq levels of all time points compared to the levels in early S for genes >100 kb replicated in early S.

(G) As for (B), with genes separated based on their P^3R^2 levels as from Figure S3F. *p < 0.05; **p < 0.01.

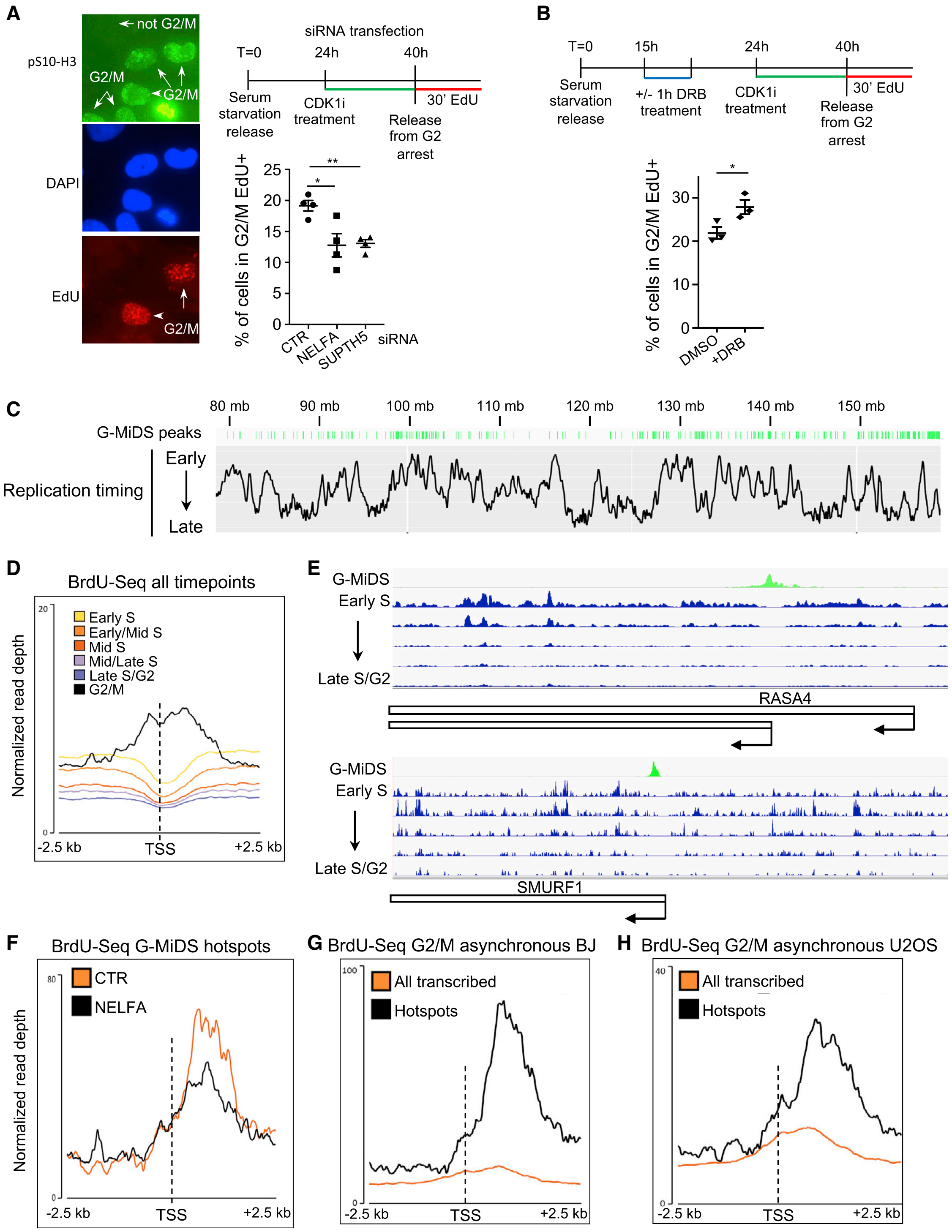

Figure 4. Persistence of RNAPII at TSSs prevents timely replication in S phase

(A) Immunofluorescence for pS10-H3 (green) for G2/M-mitotic cells, EdU Click-iT for DNA synthesis (red), and DAPI (blue) for nuclei staining. G2/M-EdU, but not mitotic-EdU, double-positive cells quantified in CTR, NELFA, and SUPT5H siRNA cells; n = 4.

(B) G2/M-EdU double-positive cells quantified in CTR DMSO and cells treated in early S for 1 h with DRB (100 μM); n = 3.

(C) Distribution of G-MiDS-specific peaks in green in relation to replication timing on the long arm of chromosome 7, as in Figure S1A.

(D) Average metagene profile for BrdU-seq levels normalized to input DNA in S-phase time points and G2/M at TSSs ±2.5 kb of transcribed genes.

(E) Snapshots from IGV TDF (Integrative Genomics Viewer tiled data file) of G-MiDS-specific BrdU-seq (green) and BrdU-seq in S-phase time points (blue) around TSSs of the indicated genes.

(legend continued on next page)

of these factors reduces RNAPII persistence at the TSS, moving it toward the gene body (Rahl et al., 2010; Fitz et al., 2018). In control (CTR) small interfering RNA (siRNA) cells, we observed that ~20% of cells were double positive for pS10-H3/EdU, suggesting a high level of late non-S-phase DNA synthesis (Figure 4A). In agreement with our hypothesis, knockdown (KD) of either NELFA or SUPTH5 reduced the frequency of pS10-H3/EdU-positive cells to 12.8% and 13.1%, respectively (Figure 4A). Moreover, treating cells in early S phase for 1 h with 5,6-dichloro-1-beta-D-ribofuranosylbenzimidazole (DRB), a CDK9 inhibitor that accumulates RNAPII near the TSS (Saponaro et al., 2014), increased the frequency of G-MiDS-positive cells to 27% from 20.9% of the CTR DMSO-treated cells (Figure 4B). Altogether, these data suggest that persistence of RNAPII near the TSS affects G-MiDS levels. Given this correlation, we decided to sequence G-MiDS sites.

To isolate cells in G2/M phase, we released cells into BrdU after Ro3306 treatment, sorted them by DNA content and pS10-H3 signal (Figure S4B), and carried out BrdU pull-down and sequencing. We identified hotspot sites of DNA synthesis by calling BrdU peaks in the G2/M sample using a bioinformatic tool. We also identified sites of DNA synthesis in all S-phase time points using the same BrdU peak calling approach and defined G-MiDS-specific sites as peaks not overlapping with those identified in other time points. This approach identified >16,000 G-MiDS sites across the genome, present in both late- and early-replicated regions (Figure 4C). Crucially, these analyses identified DNA synthesis specifically across the TSS of transcribed genes where previously there was a gap (Figures 4D and S4C). Furthermore, we also identified G-MiDS-specific peaks within the first 2 kb from the TSS over 1,306 transcripts belonging to 449 unique genes (Figure 4E).

To investigate further whether PPP regulates G-MiDS levels, we performed G-MiDS sequencing following KD of NELFA, as PPP regulation is the only role for the NELF complex in RNAPII transcription (Rahl et al., 2010; Fitz et al., 2018). Loss of NELFA reduced DNA synthesis levels specifically across the TSS of the 449 G-MiDS hotspots, in agreement with our previous finding (Figures 4F and S4D). We observed the gap at the TSS of these hotspot genes during S-phase time points, with reduced DNA synthesis across TSS in the later time points compared to all transcribed genes (Figure S4E). This suggests that once the gap has been formed, cells may wait until G2/M to complete DNA synthesis across some of these TSS. Importantly, the difference in the shape of the profiles in Figure 4D (all transcribed genes) and Figure 4F (G-MiDS hotspots) indicated that G-MiDS across TSSs was more common than just over the hotspot genes identified using the BrdU peak calling strategy. To assess whether G-MiDS could be a response to serum starvation plus Ro3306 treatment, we sorted G2/M cells as above from untreated exponentially growing BJ cells pulsed for 30 min with BrdU. The profiles confirm a peak of DNA synthesis across TSSs, which was larger across TSSs of G-MiDS hotspots (Figure 4G). Similarly, we sorted G2/M cells also from exponentially growing U2OS cells to assess whether G-MiDS was occurring only in fibroblasts. The profiles show also in this case an enrichment of DNA synthesis across TSSs that is even greater across G-MiDS TSSs, proving that G-MiDS hotspots are conserved across these two different cell lines (Figure 4H and S4F).

In concert, these results confirm that completion of DNA synthesis across TSS is a conserved process in G2/M and is linked to the persistence of RNAPII near TSS regions.

Origins firing next to TSSs exhibit asymmetric replication fork progression

Previous data showed that origins of replication are enriched next to TSSs of transcribed genes (Dellino et al., 2013; Cayrou et al., 2015; Petryk et al., 2016; Chen et al., 2019). However, our BrdU-seq analysis identified a gap of DNA synthesis across the TSSs of active genes. To determine how under-replicated DNA could occur next to an origin of replication, we reanalyzed data from studies using strand specific Okazaki fragments sequencing (Ok-seq). By analyzing Okazaki fragment levels for both lagging strands departing from the same replication origin, we could compare the efficiency of replication forks departing in the opposite direction. We used datasets from Chen et al. (2019), which were generated from immortalized fibroblasts, as DNA replication programs are highly conserved among fibroblasts (Figure S5A). Our reanalyses gave the same conclusions as in the Chen et al. paper, with origins of replication enriched near the TSSs of transcribed genes >100 kb and preferential arrangement of the replication origins, so that leading forks progress through genes in the same direction of transcription (Figure 5A). However, when we looked more closely at the TSS region, we could see that the positions where the two lagging-strand levels are at their highest were not the same (Figure 5B, black and orange arrows), with one upstream and the other downstream of the TSS. These data suggest that it is specifically the lagging replication fork moving toward the TSS that can be affected by the presence of RNAPII. Indeed, if we analyze Ok-Seq data without strand specificity, we observe a gap across the TSS (Figure 5C). The depth of this gap was smaller than the one observed in our BrdU-Seq, probably because the *Chen et al.* analysis was performed in asynchronous cells, identifying both gap formation at the time of replication and gap filling later during S-G2 phases, as shown in our Figure 3F. Interestingly, the defect in Okazaki fragment synthesis appeared more marked at G-MiDS-hotspot TSSs (Figures 5C and S5B). This agrees with our previous data that showed that once the gap has been formed, cells have to wait for G2/M to complete DNA synthesis across those sites (Figure S4E). This uncoupling between the positions of the lagging-strand synthesis start could be observed also in genes of medium length, although to a lesser extent, like for our TSS gap (Figure S5C). Importantly, and in agreement with our data,

(F) Average metagene profile for the BrdU-seq levels normalized to input DNA at TSS ±2.5 kb of the 449 hotspot genes in cells transfected with the denoted siRNA.
(G) As for (D) across all transcribed genes and only G-MiDS hotspots for asynchronous BJ cells treated with BrdU for 30 min and sorted in G2/M.
(H) As for (G), with U2OS cells.
Data represent mean ± SEM; Student's t test; *p < 0.05; **p < 0.01.

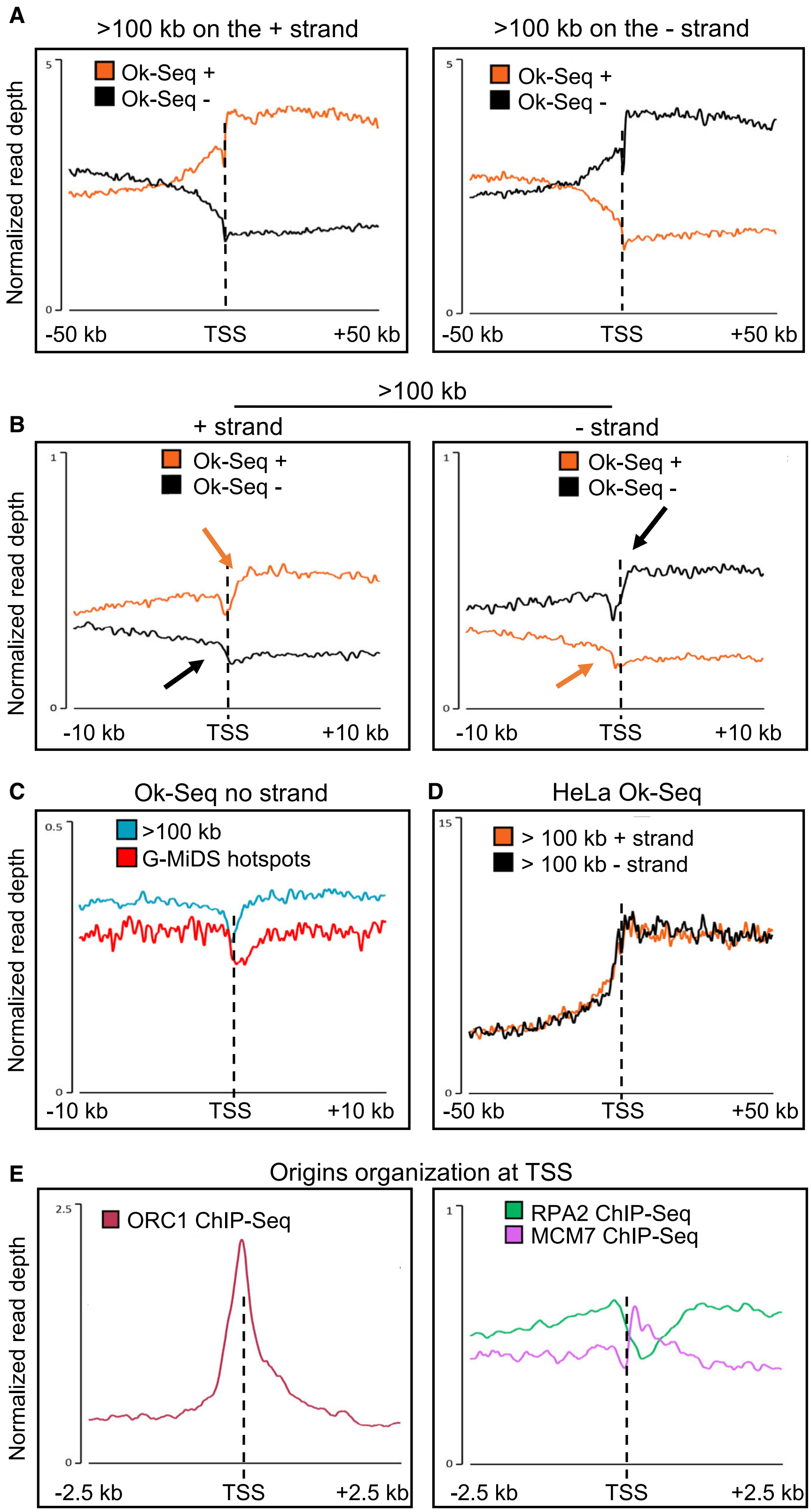

Figure 5. Uncoupling of replication forks efficiency at origins of replication near TSSs

(A) Average metagene profile for the denotated strand of strand-specific Ok-seq from Chen et al. (2019) TSSs ±50 kb of transcribed genes >100 kb in BJ-hTERT cells.

(B) As for (A) but for TSSs ±10 kb, with orange and black arrows indicating the start positions of the Okazaki fragments on "+" or "−" strands.

(C) Average metagene profile for Ok-seq from Chen et al. (2019) TSSs ±10 kb of transcribed genes >100 kb or G-MiDS hotspot genes in BJ-hTERT without strand specificity.

(D) Average metagene profile for Ok-seq transcribed/not-transcribed strand from Petryk et al. (2016) TSSs ± 50 kb of transcribed genes >100 kb in HeLa cells on + or − strands.

(E) Average metagene profile of MCM7 (Sugimoto et al., 2018), RPA2 (Zhang et al., 2017), and ORC1 (Dellino et al., 2013) ChIP-seq in HeLa cells at TSSs of transcribed genes >100 kb.

CellPress
OPEN ACCESS

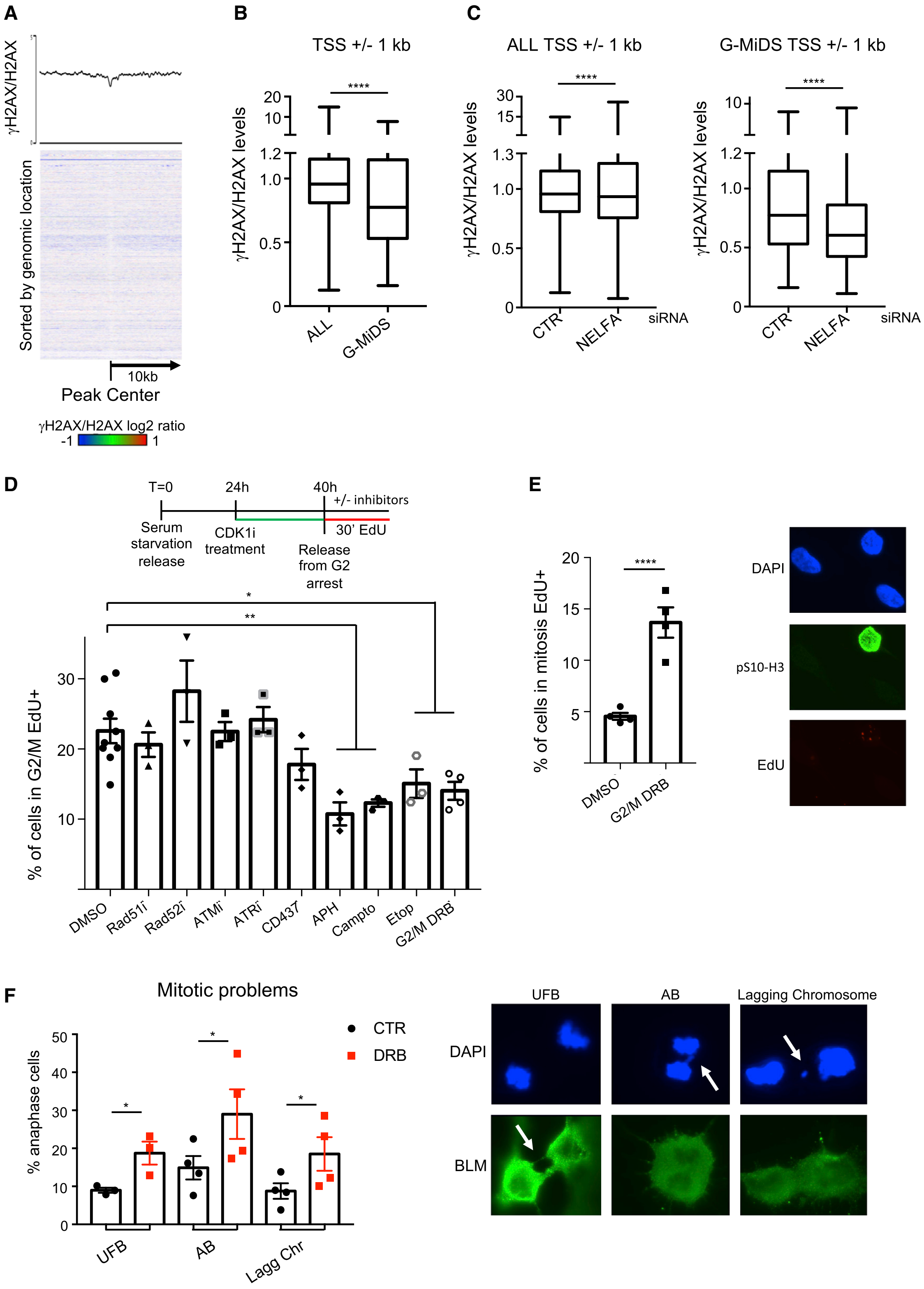

Figure 6. G-MiDS is not dependent on the DNA damage response and is distinct from MiDAS

(A) Average metagene profile and heatmap for γH2AX/H2AX at G-MiDS-specific peaks ±10 kb.

(B) Quantification of γH2AX/H2AX levels around TSSs ±1 kb of all transcribed genes and G-MiDS hotspots.

(legend continued on next page)

other transcription features like TTSs or enhancers did not show defects in Okazaki fragment distribution (Figures S5D and S5E), even though these have been identified as sites of replication termination or initiation (Chen et al., 2019).

We also investigated whether a similar phenotype could be observed in other cell types, reanalyzing Ok-seq data from HeLa cells (Petryk et al., 2016). As above, these data also demonstrated that replication forks are aligned with gene transcription in actively transcribed long genes (>100 kb) (Figure 5D). To analyze replication origin efficiency, we also analyzed MCM7 (Sugimoto et al., 2018), RPA2 (Zhang et al., 2017), and ORC1 (Dellino et al., 2013) HeLa chromatin immunoprecipitation sequencing (ChIP-seq) data at the TSSs of HeLa transcribed genes >100 kb. We found that ORC1 accumulates at TSSs as previously described (Figure 5E). However, the distribution of MCM7 was polarized toward gene transcription direction, as observed for Ok-seq data (Figure 5E). Furthermore, RPA2 was reduced in levels across TSSs, suggesting perhaps that under-replicated TSSs might not be single stranded (Figure 5E). We also analyzed RPA2 and MCM7 levels around the TSSs of G-MiDS hotspots, finding a slight accumulation only of RPA2 upstream of TSSs (Figure S5F). This would suggest that when MCM complexes get in the proximity of G-MiDS TSSs, they do not persist there waiting for the RNAPII to be removed in G2/M to complete duplication of the TSSs.

These results suggested that although origins of replication were activated next to TSSs, the efficiency of the replication forks moving from these origins could be different. Replication forks moving toward the TSS could be hindered by the presence of RNAPII at TSSs. This is much more severe at genes >100 kb, as these have the highest levels of PPP (Figure 3G) and preserve the highest levels of RNAPII at TSSs during replication (Figure S2D). Origins of replication will not be activated next to the TSS of every transcribed gene (Chen et al., 2019); therefore, for all other TSSs, we postulate that when a replication fork reaches these regions, it may encounter RNAPII, and this will lead to the formation of the BrdU gap.

G-MiDS is not associated with DNA damage or canonical DNA damage repair pathways

Transcribed regions present increased levels of endogenous DNA damage and are chromosomal translocations and double-strand breaks hotspots (Iacovoni et al., 2010; Chiarle et al., 2011; Seo et al., 2012; Kantidakis et al., 2016; Yan et al., 2017). At the same time, DNA damage increases specifically during S phase in unperturbed cells (Saldivar et al., 2018). Hence, we analyzed whether deferring duplication of TSS in G2/M was associated with increased DNA damage levels. To this end, we performed ChIP-seq for phospho-Ser139 H2AX (hereafter γH2AX) as a proxy for DNA damage (Iacovoni et al., 2010; Kantidakis et al., 2016) in asynchronous cells and for total histone H2AX for normalization.

First, we analyzed the impact of G-MiDS on DNA damage levels, as mitotic DNA synthesis (hereafter MiDAS) had been proposed as a last resource DNA damage repair process following DNA replication stress, through RAD52-dependent break-induced replication (BIR) (Minocherhomji et al., 2015). Intriguingly, average profile and heatmap of ChIP-seq data showed that G-MiDS sites are not enriched in γH2AX, indicating that they were not generally associated with sites of DNA damage (Figure 6A). Following, we quantified the levels of γH2AX around the TSSs ±1 kb of the 449 G-MiDS hotspot genes compared to all transcribed genes, finding a decrease in γH2AX levels over G-MiDS hotspots (Figure 6B). Then, we performed the same analysis also upon the KD of NELFA to assess how changing RNAPII levels at TSSs impacted on genome stability. We found that KD of NELFA limitedly reduced DNA damage levels around the TSSs but had a greater impact on the 449 G-MiDS-hotspot TSSs (Figure 6C). As G-MiDS was also present in U2OS cells, we reanalyzed data from Clouaire et al. (2018) for ChIP-seq levels of the DNA damage repair factors RAD51, 53BP1, and XRCC4 across TSSs. Also in this case, no difference was found in DNA damage repair factors levels across G-MiDS hotspots versus all transcribed genes (Figure S6A). These data appear to indicate that G-MiDS sites are not associated with DNA damage or the recruitment of DNA damage repair factors, although reducing G-MiDS levels, like NELFA KD, might also reduce the risk of incurring DNA damage.

Next, we assessed whether G-MiDS was dependent on canonical DNA damage repair factors/pathways. For this, we adopted our Ro3306 protocol to quantify G2/M-EdU double positive cells in the presence of inhibitors against specific DNA repair factors. Inhibition of RAD52 (BIR) or RAD51 (homologous recombination repair) or the apical DNA damage checkpoint kinases ATM and ATR had no effect on G-MiDS levels (Figures 6D, S6B, and S6C). However, we found that treatment with aphidicolin, a replicative DNA polymerase inhibitor, reduced G2/M-EdU double-positive cell frequency and DNA synthesis levels in positive cells (Figures 6D and S6D), indicating that G-MiDS is executed by replicative polymerases. This was also the case when cells were treated with the DNA-polymerase-alpha-specific inhibitor CD437, although this was not a significant reduction (Figure 6D). We also hypothesized that the extension of DNA synthesis across the TSS may lead to topological constraints and therefore tested the impact of topoisomerase I and II inhibitors. Indeed, we found that both a topoisomerase I inhibitor (camptothecin) and a topoisomerase II inhibitor (etoposide) reduced the frequency of DNA synthesis in G2/M cells

(C) Quantification of γH2AX/H2AX levels around TSSs ±1 kb of all transcribed genes and G-MiDS hotspots in CTR cells and after KD of NELFA.
(D) G2/M-EdU double-positive cells quantified in CTR DMSO cells or with the inhibitors (Rad51i = 25 μM, Rad52i = 20 μM, ATMi = 10 μM, ATRi = 4 μM, CD437 = 5 μM, aphidicolin [APH] = 10 μM, camptothecin [Campto] = 1 μM, and etoposide [Eto] = 10 μM) for 30 min once released from G2 arrest; DRB (100 μM) treated for 1 h before and then 30 min once released from G2 arrest; $n \geq 3$.
(E) Immunofluorescence for pS10-H3 (green) to label mitotic cells, EdU Click-iT for DNA synthesis (red), and DAPI (blue) for nuclei staining. Only mitotic-EdU double-positive cells from prometaphase on were quantified; cells were treated with DMSO or DRB as in (D); n = 4.
(F) Immunofluorescence for Bloom (BLM; green) and DAPI (blue) for nuclei staining in cells released after the Ro3306 G2 arrest for 80 min in DMSO or DRB (100 μM), quantifying ultrafine bridges (UFB), anaphase bridges (AB), and lagging chromosomes (Lagg Chr), with examples highlighted by white arrows; $n \geq 3$. Data represent mean ± SEM; Student's t test; ns, not significant; *p < 0.05; **p < 0.01; ****p < 0.0001.

CellPress
OPEN ACCESS

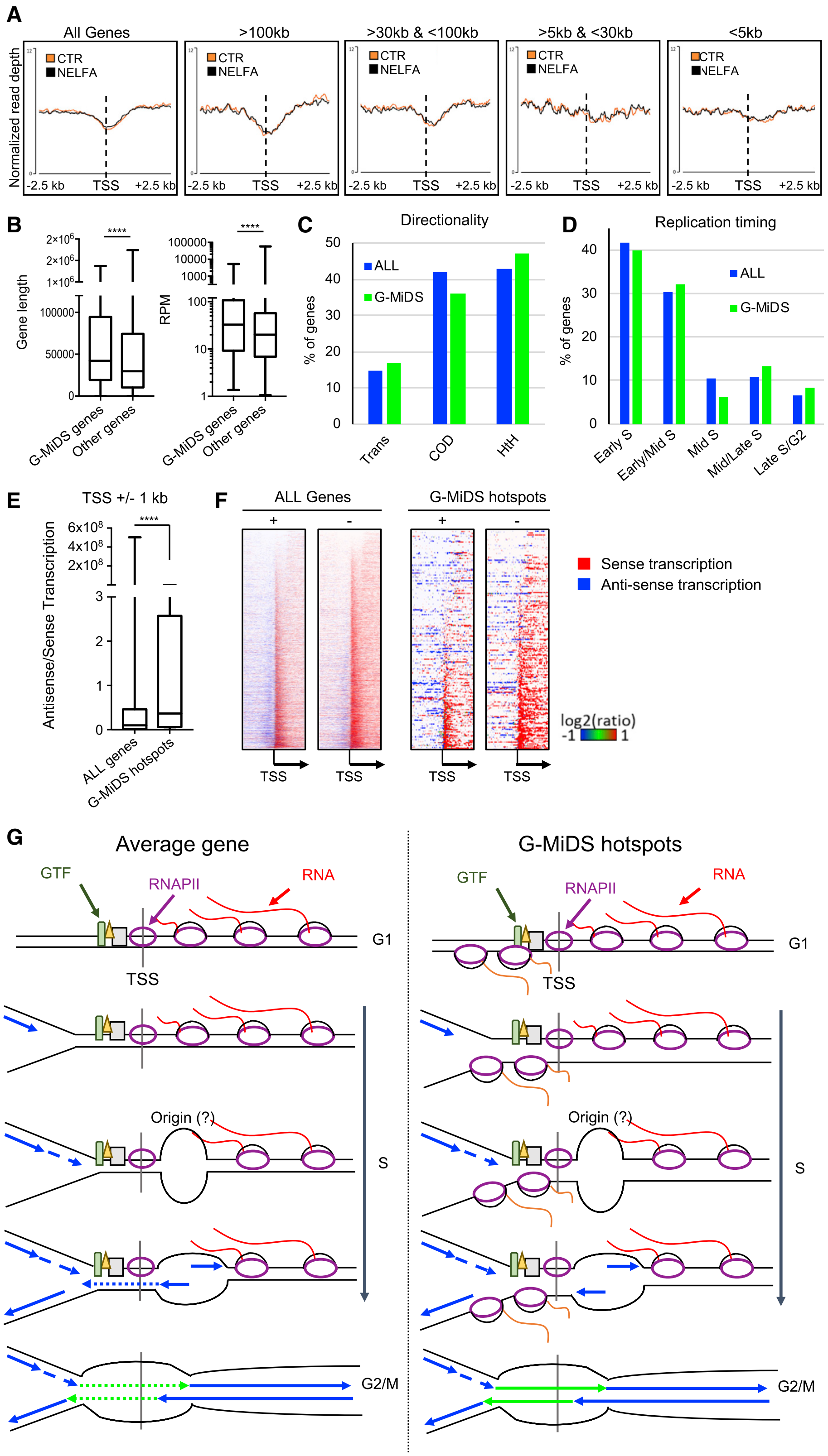

(legend on next page)

(Figure 6D). Finally, to link G-MiDS to RNAPII removal from the TSS, we released cells from the Ro3306 into DRB and found a significant reduction in G2/M DNA synthesis (Figure 6D). Importantly, while treatment with DRB reduced G-MiDS levels, it led to a 3-fold increase in MiDAS levels (Figure 6E), which was associated with a 2-fold increase in ultrafine bridges, anaphase bridges, and lagging chromosomes (Figure 6F). All these findings support our conclusion that G-MiDS is a process distinct from MiDAS and that in fact hindering G-MiDS can lead to an increase in genome instability levels.

G-MiDS-hotspot-specific features

Given that NELFA KD affected DNA synthesis levels across TSSs in G2/M, we analyzed whether PPP is also responsible for BrdU gap formation. However, KD of either NELFA or SUPT5H had no impact on BrdU gap formation independently of gene length or directionality (Figures 7A and S7A). This suggests that while reducing PPP levels creates a "window of opportunity" to complete duplication across TSSs before G2/M, PPP is not responsible for the formation of BrdU gaps in the first instance. We also tested the impact of triptolide, a TFIIH (transcription factor II H) inhibitor that reduces RNAPII levels at the TSS by blocking transcription initiation (Erickson et al., 2018). A short treatment with triptolide at the beginning of S phase had only a minor effect on BrdU gap formation, indicating that these gaps are also dependent on the whole set of transcription factors that regulate gene transcription at promoters (Figure S7B).

Finally, we aimed to identify specific features characterizing G-MiDS hotspot genes. Overall, these 449 genes were longer and more transcribed than other transcripts (Figure 7B), enriched for genes orientated in a head-to-head configuration (Figure 7C; chi-square p value = 0.033). Moreover, they were replicated at later stages of S phase compared to the general distribution, even though the majority were still replicated at the beginning of S phase (Figure 7D; chi-square p value = 0.012). Gene Ontology analysis showed enrichment in transcription factors, GTPases, cell migration, and vesicular transport factors (Table S4), with transcription levels stable throughout the cell cycle (Figure S7C). Importantly, these genes were not specifically transcribed in mitosis in HUH7 and HeLa cells (hypergeometric p value = 0.003 depleted for overlap in HUH7 cells and 0.458 for enrichment in HeLa cells), suggesting that on these genes, RNAPII will be removed when cells prepare for mitosis (Liang et al., 2015; Palozola et al., 2017).

We also analyzed how transcription is arranged at TSSs of G-MiDS hotspots, quantifying the levels of sense and antisense transcription at TSSs. Intriguingly, G-MiDS hotspot genes had on average 3.8-fold more antisense/sense transcription across TSSs when compared to all transcribed genes. This is highlighted by heatmaps for sense (in red) and antisense (in blue) transcription across the TSS region (Figures 7E and 7F). This antisense transcription was genuine TSS-associated antisense, as only 14.7% of the G-MiDS hotspots had a bidirectional promoter with a transcribed gene on the other side.

Taken together, these data suggest that because G-MiDS genes are stably expressed throughout the cell cycle with high levels of both sense and antisense transcription, this will render these TSSs particularly impassable for the replication machinery. Therefore, cells require passage into G2/M and the removal of RNAPII from TSSs to complete DNA synthesis.

DISCUSSION

RNA transcription and DNA replication are the two essential processes that allow cells to express the content of their genomes and generate two identical copies to pass on to daughter cells. As both processes use the DNA as a template, crosstalk is crucial to avoid reciprocal interference. Using genomic approaches, we have now uncovered the reciprocal impact of RNAPII transcription and DNA replication. Our data indicate that transcription is still active during DNA replication, with important consequences for timing of replication (Figures 3 and 4) and replication fork progression (Figure 5). In parallel, we observe a transient shutdown of gene transcription only when a gene is replicated (Figure 2).

However, the most surprising finding is that TSSs of transcribed genes can remain under-replicated throughout S phase, with TSSs completely duplicated in hundreds of cases only when cells are preparing for mitosis (Figure 4D). We showed a direct correlation between RNAPII levels at TSSs and G-MiDS levels. The PPP role in transcription regulation is important also to preserve nucleosome organization at TSSs to maintain active gene transcription (Gilchrist et al., 2010; Core et al., 2012). Indeed, in newly replicated chromatin, the positional information of histone marks is conserved (Reverón-Gómez et al., 2018). One possibility could be that maintaining the RNAPII at the TSS might be required to maintain the nucleosome organization around TSSs when the region is replicated. The RNAPII would act as a "signpost" for TSSs, particularly relevant perhaps for longer genes where we identify wider and deeper BrdU gaps (Figure 3B), and that preserve the greatest levels of RNAPII in newly replicated chromatin (Figure S2D). This would allow duplication of

Figure 7. G-MiDS-hotspot-gene-specific features

(A) As in Figures 3A and 3B for all transcribed genes and transcripts separated by gene length in cells transfected with the indicated siRNA.
(B) Gene length and transcription levels at the late-S/G2 time point for G-MiDS hotspot genes toward all other transcribed genes.
(C) Directionality analysis of G-MiDS hotspot genes compared with directionality of all transcribed genes.
(D) Replication timing of G-MiDS hotspot genes compared with all transcribed genes.
(E) Quantification of the ratio between antisense and sense transcription at TSSs ±1 kb of all transcribed genes and the 449 G-MiDS hotspots.
(F) Heatmap analysis of the levels of antisense and sense transcription at TSSs ±2.5 kb for all the transcribed genes and G-MiDS hotspots on the + and − strands.
(G) Model describing how the TSS is occupied by RNAPII and general transcription factors (GTF) throughout the cell cycle, with the RNAPII moving along genes. When DNA replication approaches the TSS during S phase, it may encounter GTF/RNAPII, skipping the TSS and restarting downstream of it. This may be mediated by the activation of origins of replication near TSSs. Later during S phase, DNA replication may fill the resulting gaps. However, in cases of genes with high steady expression levels of sense and TSS-associated antisense transcription, completion of the duplication of TSSs will occur in G2/M, when RNAPII and GTF are removed from TSSs. Box and whisker plots with the line at the median; Mann-Whitney t test; ****$p < 0.0001$.

the DNA preserving chromatin organization in order to create a fully functional genome to pass on to daughter cells.

We identified 449 hotspot G-MiDS TSSs characterized by a series of features (Figures 7B–7E). High levels of sense and antisense transcription would make the TSS particularly difficult to replicate across, as indicated also by our relative enrichment profiles (Figure S4E). In parallel, enhancers that are also characterized by high levels of antisense transcription show no enrichment for G2/M DNA synthesis, as their impact on DNA replication is very limited (Figure S3E; data not shown). Determining how altering specifically antisense levels at G-MiDS-hotspot TSSs would allow these TSSs to be replicated within S phase is an important aspect to investigate in the future. Importantly, the NELF complex regulates both sense and antisense transcription levels at TSSs (Figure S7D), suggesting that upon the KD of NELFA, both sense and antisense RNAPII will be moved away from the TSS, making TSSs overall more accessible throughout S phase.

A large body of literature has shown how replication origins are enriched around TSSs of transcribed genes (Dellino et al., 2013; Cayrou et al., 2015; Petryk et al., 2016; Chen et al., 2019). However, for origins of replication activated near a TSS, the replication forks that will move from such origins may have different efficiencies depending on whether they get toward the TSS or away from it (Figure 5).

Contexts with defective transcription often present higher DNA damage levels (Huertas and Aguilera, 2003; Dutta et al., 2011; Sollier et al., 2014). Although our study does not analyze R-loops, we do not predict a role for physiologic R-loops in the phenotypes we observe. R-loops occur at the 5′ and 3′ end of genes and have roles in maintaining chromatin marks and regulating transcription termination (Ginno et al., 2013; Skourti-Stathaki et al., 2014; Chen et al., 2015). Nevertheless, we do not observe phenotypes at the 3′ end of genes (Figure S3C). Moreover, although GC-skewed promoters are more prone to R-loops (Ginno et al., 2013), BrdU gaps do not occur preferentially at GC-skewed promoters (Figure S7E), but more generally at GC-rich promoters associated with longer genes and higher transcription levels (Figures S7F and S7G). R-loops were also shown to be important to regulate antisense transcription at TSSs (Tan-Wong et al., 2019). We reanalyzed data from Tan-Wong et al., confirming that over all transcribed genes, the overexpression of RNASEH1 leads to a more pronounced reduction of antisense transcription (Figure S7H). However, for G-MiDS hotspot genes, the reduction in sense and antisense transcription is the same (Figure S7H), suggesting that R-loops may play a role in supporting sense transcription over these genes as previously shown (Chen et al., 2015).

MiDAS was first described as a DNA damage repair mechanism that supports DNA duplication following replication stress (Minocherhomji et al., 2015; Bhowmick et al., 2016). In our experiments, we have shown that DNA synthesis in G2/M is not at DNA damage sites and does not depend on canonical DNA repair mechanisms (Figures 6B and 6D). However, altering RNAPII levels at TSSs either through the KD of NELFA or by maintaining the RNAPII at TSSs in G2/M with DRB impacts genome stability, affecting γH2AX, MiDAS, and defective mitosis (Figures 6C, 6E, and 6F). G-MiDS, therefore, is a process separated from MiDAS that is likely responsible for completion of DNA replication in G2/M, with MiDAS coming in mitosis as a DNA damage repair process. As origins of replication cannot be activated once cells pass the G1/S transition (Fragkos et al., 2015), and because of the overall defect in Okazaki fragments at TSS (Figure 5C), we envisage that G-MiDS is a gap filling process dependent on replicative polymerases. Replicative polymerases have the highest fidelity among DNA polymerases, preserving the genetic information present in those regions. Moreover, we have also identified other components of G-MiDS like topoisomerases I and II, both of which to our knowledge have never been shown to have a role in BIR.

Surprisingly, we identify G2/M-specific replication occurring in one in five cells (Figures 4A and 4B), and DNA synthesis in late G2/M had already been described prior to MiDAS (Hansen et al., 1993; Widrow et al., 1998). Following DNA replication stress, the DNA damage checkpoint will block cells from entering mitosis; however, there are instances where the DNA damage checkpoint is not activated in the presence of under-replicated DNA (Bergoglio et al., 2013; Yang et al., 2017). Very likely, therefore, the under-replicated DNA at TSSs is not exposed as single-stranded DNA or bound by RPA or RAD51 (Figures 5E and S6A), persisting in some form of X-shaped molecule tolerated by cells throughout the cell cycle.

Genome instability can arise as consequence of failed G-MiDS at TSSs. Intriguingly, 26 out of the 449 G-MiDS hotspot genes are listed as cancer genes in the COSMIC database, and many are associated with copy-number alterations and/or translocations (Figure S7I; hypergeometric p value = 0.052). Whether deferring the duplication of the TSS to G2/M comes with a risk that these TSSs do not get fully replicated, leading to the aberrations observed in cancer, is a very exciting speculative avenue that will need further investigations.

Limitations of study

Current limitations in single-cell sequencing allow assessment at the population level of the reciprocal impact of transcription and replication at hotspots where interference is happening at high frequency (i.e., TSSs). Future developments in single-cell analysis will allow expanding this study to rarer events, proving that the two processes are happening at the same time on the same molecule of DNA. Moreover, it will be important in the future to assess whether reducing specifically antisense transcription levels at G-MiDS-hotspot TSSs is sufficient to allow duplication of TSS regions during S phase.

STAR★METHODS

Detailed methods are provided in the online version of this paper and include the following:

- KEY RESOURCES TABLE
- RESOURCE AVAILABILITY
  - Lead contact
  - Materials availability
  - Data and code availability
- EXPERIMENTAL MODEL AND SUBJECT DETAILS

- METHOD DETAILS
  - Growing conditions and DNA Labeling
  - BrdU-seq
  - Chromatin-bound RNA sequencing (Chr-RNA-seq)
  - Nascent transcription levels analysis
  - Cell cycle analysis
  - Transcription activity by EU incorporation analysis by FACS
  - siRNA transfection
  - Western blotting
  - Immunostaining
  - ChIP-seq
  - Cell sorting by flow cytometry and G2/M BrdU-seq
  - Datasets alignment
  - Correlation
  - Peak calling
  - Replication and transcription reciprocal directionality
  - Normalized profiles
  - ChIP-seq and RNA-seq quantifications
- QUANTIFICATION AND STATISTICAL ANALYSES

SUPPLEMENTAL INFORMATION

Supplemental Information can be found online at https://doi.org/10.1016/j.celrep.2021.108759.

ACKNOWLEDGMENTS

We thank the Genomics Birmingham and Flow Cytometry Facility at the University of Birmingham for sequencing our samples, help with NGS library preparation, and sorting mitotic cells. We thank Prof. Frederic Chedin for sharing the coordinates of the promoter classes based on GC content and GC skew level and Dr. Ran Elkon for sharing GRO-seq files for BJ-hTERT cells. We thank Aga Gambus and all members of the BCGB for valuable discussions. We thank the Higgs lab members for sharing protocols and reagents. This work was supported by a University of Birmingham fellowship, the Wellcome Trust (202115/Z/16/Z), the Royal Society (RG170246), the BBSRC (BB/S016155/1), and Cancer Research UK (C17422/A25154) to M.S.; a China Scholarship Council scholarship to S.X.; a University of Birmingham fellowship to M.R.H.; and the Medical Research Council (MR/M009882/1 to M.R.H. and MR/K01076X/1 to P.G.).

AUTHOR CONTRIBUTIONS

Conceptualization, M.S.; methodology, J.W. and P.R.; investigation, J.W., P.R., J.M., M.M.S., O.G., S.X., and M.S.; writing, M.R.H., J.W., P.R., P.G., and M.S.; resources, M.S., M.R.H., and P.G.; funding acquisition, M.S., M.R.H., and P.G.; supervision, M.S.

DECLARATION OF INTERESTS

The authors declare no competing interests.

Received: November 21, 2019
Revised: November 9, 2020
Accepted: January 26, 2021
Published: February 16, 2021

REFERENCES

Afgan, E., Baker, D., Batut, B., van den Beek, M., Bouvier, D., Cech, M., Chilton, J., Clements, D., Coraor, N., Grüning, B.A., et al. (2018). The Galaxy platform for accessible, reproducible and collaborative biomedical analyses: 2018 update. Nucleic Acids Res. *46* (*W1*), W537–W544.

Akamatsu, Y., and Kobayashi, T. (2015). The human RNA polymerase I transcription terminator complex acts as a replication fork barrier that coordinates the progress of replication with rRNA transcription activity. Mol. Cell. Biol. *35*, 1871–1881.

Alzu, A., Bermejo, R., Begnis, M., Lucca, C., Piccini, D., Carotenuto, W., Saponaro, M., Brambati, A., Cocito, A., Foiani, M., and Liberi, G. (2012). Senataxin associates with replication forks to protect fork integrity across RNA-polymerase-II-transcribed genes. Cell *151*, 835–846.

Bergoglio, V., Boyer, A.S., Walsh, E., Naim, V., Legube, G., Lee, M.Y., Rey, L., Rosselli, F., Cazaux, C., Eckert, K.A., and Hoffmann, J.S. (2013). DNA synthesis by Pol η promotes fragile site stability by preventing under-replicated DNA in mitosis. J. Cell Biol. *201*, 395–408.

Bermejo, R., Capra, T., Gonzalez-Huici, V., Fachinetti, D., Cocito, A., Natoli, G., Katou, Y., Mori, H., Kurokawa, K., Shirahige, K., and Foiani, M. (2009). Genome-organizing factors Top2 and Hmo1 prevent chromosome fragility at sites of S phase transcription. Cell *138*, 870–884.

Bhowmick, R., Minocherhomji, S., and Hickson, I.D. (2016). RAD52 facilitates mitotic DNA synthesis following replication stress. Mol. Cell *64*, 1117–1126.

Brewer, B.J. (1988). When polymerases collide: replication and the transcriptional organization of the E. coli chromosome. Cell *53*, 679–686.

Brewer, B.J., and Fangman, W.L. (1988). A replication fork barrier at the 3′ end of yeast ribosomal RNA genes. Cell *55*, 637–643.

Bushnell, B., Rood, J., and Singer, E. (2017). BBMerge - Accurate paired shotgun read merging via overlap. PLoS ONE *12*, e0185056.

Cayrou, C., Ballester, B., Peiffer, I., Fenouil, R., Coulombe, P., Andrau, J.C., van Helden, J., and Méchali, M. (2015). The chromatin environment shapes DNA replication origin organization and defines origin classes. Genome Res. *25*, 1873–1885.

Chen, P.B., Chen, H.V., Acharya, D., Rando, O.J., and Fazzio, T.G. (2015). R loops regulate promoter-proximal chromatin architecture and cellular differentiation. Nat. Struct. Mol. Biol. *22*, 999–1007.

Chen, Y.H., Keegan, S., Kahli, M., Tonzi, P., Fenyö, D., Huang, T.T., and Smith, D.J. (2019). Transcription shapes DNA replication initiation and termination in human cells. Nat. Struct. Mol. Biol. *26*, 67–77.

Chiarle, R., Zhang, Y., Frock, R.L., Lewis, S.M., Molinie, B., Ho, Y.J., Myers, D.R., Choi, V.W., Compagno, M., Malkin, D.J., et al. (2011). Genome-wide translocation sequencing reveals mechanisms of chromosome breaks and rearrangements in B cells. Cell *147*, 107–119.

Clouaire, T., Rocher, V., Lashgari, A., Arnould, C., Aguirrebengoa, M., Biernacka, A., Skrzypczak, M., Aymard, F., Fongang, B., Dojer, N., et al. (2018). Comprehensive mapping of histone modifications at DNA double-strand breaks deciphers repair pathway chromatin signatures. Mol. Cell *72*, 250–262.e6.

Core, L.J., Waterfall, J.J., Gilchrist, D.A., Fargo, D.C., Kwak, H., Adelman, K., and Lis, J.T. (2012). Defining the status of RNA polymerase at promoters. Cell Rep. *2*, 1025–1035.

Dale, R.K., Pedersen, B.S., and Quinlan, A.R. (2011). Pybedtools: a flexible Python library for manipulating genomic datasets and annotations. Bioinformatics *27*, 3423–3424.

Dellino, G.I., Cittaro, D., Piccioni, R., Luzi, L., Banfi, S., Segalla, S., Cesaroni, M., Mendoza-Maldonado, R., Giacca, M., and Pelicci, P.G. (2013). Genome-wide mapping of human DNA-replication origins: levels of transcription at ORC1 sites regulate origin selection and replication timing. Genome Res. *23*, 1–11.

Deshpande, A.M., and Newlon, C.S. (1996). DNA replication fork pause sites dependent on transcription. Science *272*, 1030–1033.

Djebali, S., Davis, C.A., Merkel, A., Dobin, A., Lassmann, T., Mortazavi, A., Tanzer, A., Lagarde, J., Lin, W., Schlesinger, F., et al. (2012). Landscape of transcription in human cells. Nature *489*, 101–108.

Dobin, A., Davis, C.A., Schlesinger, F., Drenkow, J., Zaleski, C., Jha, S., Batut, P., Chaisson, M., and Gingeras, T.R. (2013). STAR: ultrafast universal RNA-seq aligner. Bioinformatics *29*, 15–21.

Dutta, D., Shatalin, K., Epshtein, V., Gottesman, M.E., and Nudler, E. (2011). Linking RNA polymerase backtracking to genome instability in E. coli. Cell *146*, 533–543.

Erickson, B., Sheridan, R.M., Cortazar, M., and Bentley, D.L. (2018). Dynamic turnover of paused Pol II complexes at human promoters. Genes Dev. *32*, 1215–1225.

Fitz, J., Neumann, T., and Pavri, R. (2018). Regulation of RNA polymerase II processivity by Spt5 is restricted to a narrow window during elongation. EMBO J. *37*, e97965.

Fragkos, M., Ganier, O., Coulombe, P., and Méchali, M. (2015). DNA replication origin activation in space and time. Nat. Rev. Mol. Cell Biol. *16*, 360–374.

Friendly, M. (2002). Corrgrams: exploratory displays for correlation matrices. Am. Stat. *56*, 316–324.

Gaillard, H., and Aguilera, A. (2016). Transcription as a threat to genome integrity. Annu. Rev. Biochem. *85*, 291–317.

Gentleman, R.C., Carey, V.J., Bates, D.M., Bolstad, B., Dettling, M., Dudoit, S., Ellis, B., Gautier, L., Ge, Y., Gentry, J., et al. (2004). Bioconductor: open software development for computational biology and bioinformatics. Genome Biol. *5*, R80, Published online September 15, 2004.

Gilchrist, D.A., Dos Santos, G., Fargo, D.C., Xie, B., Gao, Y., Li, L., and Adelman, K. (2010). Pausing of RNA polymerase II disrupts DNA-specified nucleosome organization to enable precise gene regulation. Cell *143*, 540–551.

Ginno, P.A., Lim, Y.W., Lott, P.L., Korf, I., and Chédin, F. (2013). GC skew at the 5′ and 3′ ends of human genes links R-loop formation to epigenetic regulation and transcription termination. Genome Res. *23*, 1590–1600.

Hamperl, S., and Cimprich, K.A. (2016). Conflict resolution in the genome: how transcription and replication make it work. Cell *167*, 1455–1467.

Hansen, R.S., Canfield, T.K., Lamb, M.M., Gartler, S.M., and Laird, C.D. (1993). Association of fragile X syndrome with delayed replication of the FMR1 gene. Cell *73*, 1403–1409.

Hansen, R.S., Thomas, S., Sandstrom, R., Canfield, T.K., Thurman, R.E., Weaver, M., Dorschner, M.O., Gartler, S.M., and Stamatoyannopoulos, J.A. (2010). Sequencing newly replicated DNA reveals widespread plasticity in human replication timing. Proc. Natl. Acad. Sci. USA *107*, 139–144.

Hassan, A.B., Errington, R.J., White, N.S., Jackson, D.A., and Cook, P.R. (1994). Replication and transcription sites are colocalized in human cells. J. Cell Sci. *107*, 425–434.

Helmrich, A., Ballarino, M., and Tora, L. (2011). Collisions between replication and transcription complexes cause common fragile site instability at the longest human genes. Mol. Cell *44*, 966–977.

Huertas, P., and Aguilera, A. (2003). Cotranscriptionally formed DNA:RNA hybrids mediate transcription elongation impairment and transcription-associated recombination. Mol. Cell *12*, 711–721.

Iacovoni, J.S., Caron, P., Lassadi, I., Nicolas, E., Massip, L., Trouche, D., and Legube, G. (2010). High-resolution profiling of gammaH2AX around DNA double strand breaks in the mammalian genome. EMBO J. *29*, 1446–1457.

Kantidakis, T., Saponaro, M., Mitter, R., Horswell, S., Kranz, A., Boeing, S., Aygün, O., Kelly, G.P., Matthews, N., Stewart, A., et al. (2016). Mutation of cancer driver MLL2 results in transcription stress and genome instability. Genes Dev. *30*, 408–420.

Langmead, B., and Salzberg, S.L. (2012). Fast gapped-read alignment with Bowtie 2. Nat. Methods *9*, 357–359.

Lerdrup, M., Johansen, J.V., Agrawal-Singh, S., and Hansen, K. (2016). An interactive environment for agile analysis and visualization of ChIP-sequencing data. Nat. Struct. Mol. Biol. *23*, 349–357.

Li, H., Handsaker, B., Wysoker, A., Fennell, T., Ruan, J., Homer, N., Marth, G., Abecasis, G., and Durbin, R.; 1000 Genome Project Data Processing Subgroup (2009). The Sequence Alignment/Map format and SAMtools. Bioinformatics *25*, 2078–2079.

Li, L., van Breugel, P.C., Loayza-Puch, F., Ugalde, A.P., Korkmaz, G., Messika-Gold, N., Han, R., Lopes, R., Barbera, E.P., Teunissen, H., et al. (2018). LncRNA-OIS1 regulates DPP4 activation to modulate senescence induced by RAS. Nucleic Acids Res. *46*, 4213–4227.

Liang, K., Woodfin, A.R., Slaughter, B.D., Unruh, J.R., Box, A.C., Rickels, R.A., Gao, X., Haug, J.S., Jaspersen, S.L., and Shilatifard, A. (2015). Mitotic transcriptional activation: clearance of actively engaged Pol II via transcriptional elongation control in mitosis. Mol. Cell *60*, 435–445.

Liao, Y., Smyth, G.K., and Shi, W. (2014). featureCounts: an efficient general purpose program for assigning sequence reads to genomic features. Bioinformatics *30*, 923–930, Published online Nov 13, 2013.

Liu, X., Gogate, A.A., Tastemel, M., Malladi, V.S., Yao, H., Nguyen, K., Huang, L.J., and Bai, X. (2017). Dynamic change of transcription pausing through modulating NELF protein stability regulates granulocytic differentiation. Blood Adv. *1*, 1358–1367.

Macheret, M., and Halazonetis, T.D. (2018). Intragenic origins due to short G1 phases underlie oncogene-induced DNA replication stress. Nature *555*, 112–116.

Marchal, C., Sasaki, T., Vera, D., Wilson, K., Sima, J., Rivera-Mulia, J.C., Trevilla-García, C., Nogues, C., Nafie, E., and Gilbert, D.M. (2018). Genome-wide analysis of replication timing by next-generation sequencing with E/L Repli-seq. Nat. Protoc. *13*, 819–839.

Maya-Mendoza, A., Moudry, P., Merchut-Maya, J.M., Lee, M., Strauss, R., and Bartek, J. (2018). High speed of fork progression induces DNA replication stress and genomic instability. Nature *559*, 279–284.

Mayer, A., and Churchman, L.S. (2016). Genome-wide profiling of RNA polymerase transcription at nucleotide resolution in human cells with native elongating transcript sequencing. Nat. Protoc. *11*, 813–833.

Minocherhomji, S., Ying, S., Bjerregaard, V.A., Bursomanno, S., Aleliunaite, A., Wu, W., Mankouri, H.W., Shen, H., Liu, Y., and Hickson, I.D. (2015). Replication stress activates DNA repair synthesis in mitosis. Nature *528*, 286–290.

Palozola, K.C., Donahue, G., Liu, H., Grant, G.R., Becker, J.S., Cote, A., Yu, H., Raj, A., and Zaret, K.S. (2017). Mitotic transcription and waves of gene reactivation during mitotic exit. Science *358*, 119–122.

Paulsen, R.D., Soni, D.V., Wollman, R., Hahn, A.T., Yee, M.C., Guan, A., Hesley, J.A., Miller, S.C., Cromwell, E.F., Solow-Cordero, D.E., et al. (2009). A genome-wide siRNA screen reveals diverse cellular processes and pathways that mediate genome stability. Mol. Cell *35*, 228–239.

Petryk, N., Kahli, M., d'Aubenton-Carafa, Y., Jaszczyszyn, Y., Shen, Y., Silvain, M., Thermes, C., Chen, C.L., and Hyrien, O. (2016). Replication landscape of the human genome. Nat. Commun. *7*, 10208.

Pourkarimi, E., Bellush, J.M., and Whitehouse, I. (2016). Spatiotemporal coupling and decoupling of gene transcription with DNA replication origins during embryogenesis in *C. elegans*. eLife *5*, e21728.

Quinlan, A.R., and Hall, I.M. (2010). BEDTools: a flexible suite of utilities for comparing genomic features. Bioinformatics *26*, 841–842.

Rahl, P.B., Lin, C.Y., Seila, A.C., Flynn, R.A., McCuine, S., Burge, C.B., Sharp, P.A., and Young, R.A. (2010). c-Myc regulates transcriptional pause release. Cell *141*, 432–445.

Ramírez, F., Dündar, F., Diehl, S., Grüning, B.A., and Manke, T. (2014). deepTools: a flexible platform for exploring deep-sequencing data. Nucleic Acids Res. *42*, W187-91.

Rausch, T., Fritz, M.H.-Y., Korbel, J.O., Benes, V., et al. (2018). Alfred: interactive multi-sample BAM alignment statistics, feature counting and feature annotation for long- and short-read sequencing. Bioinformatics *35*, 2489–2491.

Reverón-Gómez, N., González-Aguilera, C., Stewart-Morgan, K.R., Petryk, N., Flury, V., Graziano, S., Johansen, J.V., Jakobsen, J.S., Alabert, C., and Groth, A. (2018). Accurate recycling of parental histones reproduces the histone modification landscape during DNA replication. Mol. Cell *72*, 239–249.e5.

Robinson, J.T., Thorvaldsdóttir, H., Winckler, W., Guttman, M., Lander, E.S., Getz, G., and Mesirov, J.P. (2011). Integrative genomics viewer. Nat. Biotechnol. *29*, 24–26.

Saldivar, J.C., Hamperl, S., Bocek, M.J., Chung, M., Bass, T.E., Cisneros-Soberanis, F., Samejima, K., Xie, L., Paulson, J.R., Earnshaw, W.C., et al. (2018). An intrinsic S/G_2 checkpoint enforced by ATR. Science *361*, 806–810.

Saponaro, M., Kantidakis, T., Mitter, R., Kelly, G.P., Heron, M., Williams, H., Söding, J., Stewart, A., and Svejstrup, J.Q. (2014). RECQL5 controls transcript elongation and suppresses genome instability associated with transcription stress. Cell *157*, 1037–1049.

Seo, J., Kim, S.C., Lee, H.S., Kim, J.K., Shon, H.J., Salleh, N.L., Desai, K.V., Lee, J.H., Kang, E.S., Kim, J.S., and Choi, J.K. (2012). Genome-wide profiles of H2AX and γ-H2AX differentiate endogenous and exogenous DNA damage hotspots in human cells. Nucleic Acids Res. *40*, 5965–5974.

Skourti-Stathaki, K., Kamieniarz-Gdula, K., and Proudfoot, N.J. (2014). R-loops induce repressive chromatin marks over mammalian gene terminators. Nature *516*, 436–439.

Sollier, J., Stork, C.T., García-Rubio, M.L., Paulsen, R.D., Aguilera, A., and Cimprich, K.A. (2014). Transcription-coupled nucleotide excision repair factors promote R-loop-induced genome instability. Mol. Cell *56*, 777–785.

Stewart-Morgan, K.R., Reverón-Gómez, N., and Groth, A. (2019). Transcription restart establishes chromatin accessibility after DNA replication. Mol. Cell *75*, 284–297.e6.

Sugimoto, N., Maehara, K., Yoshida, K., Ohkawa, Y., and Fujita, M. (2018). Genome-wide analysis of the spatiotemporal regulation of firing and dormant replication origins in human cells. Nucleic Acids Res. *46*, 6683–6696.

Tan-Wong, S.M., Dhir, S., and Proudfoot, N.J. (2019). R-loops promote antisense transcription across the mammalian genome. Mol. Cell *76*, 600–616.e6.

Tilgner, H., Knowles, D.G., Johnson, R., Davis, C.A., Chakrabortty, S., Djebali, S., Curado, J., Snyder, M., Gingeras, T.R., and Guigó, R. (2012). Deep sequencing of subcellular RNA fractions shows splicing to be predominantly co-transcriptional in the human genome but inefficient for lncRNAs. Genome Res. *22*, 1616–1625.

Tuduri, S., Crabbé, L., Conti, C., Tourrière, H., Holtgreve-Grez, H., Jauch, A., Pantesco, V., De Vos, J., Thomas, A., Theillet, C., et al. (2009). Topoisomerase I suppresses genomic instability by preventing interference between replication and transcription. Nat. Cell Biol. *11*, 1315–1324.

van der Meijden, C.M., Lapointe, D.S., Luong, M.X., Peric-Hupkes, D., Cho, B., Stein, J.L., van Wijnen, A.J., and Stein, G.S. (2002). Gene profiling of cell cycle progression through S-phase reveals sequential expression of genes required for DNA replication and nucleosome assembly. Cancer Res. *62*, 3233–3243.

Wansink, D.G., Manders, E.E., van der Kraan, I., Aten, J.A., van Driel, R., and de Jong, L. (1994). RNA polymerase II transcription is concentrated outside replication domains throughout S-phase. J. Cell Sci. *107*, 1449–1456.

Wei, X., Samarabandu, J., Devdhar, R.S., Siegel, A.J., Acharya, R., and Berezney, R. (1998). Segregation of transcription and replication sites into higher order domains. Science *281*, 1502–1506.

Widrow, R.J., Hansen, R.S., Kawame, H., Gartler, S.M., and Laird, C.D. (1998). Very late DNA replication in the human cell cycle. Proc. Natl. Acad. Sci. USA *95*, 11246–11250.

Wolfinger, M.T., Fallmann, J., Eggenhofer, F., and Amman, F. (2015). ViennaNGS: a toolbox for building efficient next-generation sequencing analysis pipelines. F1000Res *4*, 50.

Yan, W.X., Mirzazadeh, R., Garnerone, S., Scott, D., Schneider, M.W., Kallas, T., Custodio, J., Wernersson, E., Li, Y., Gao, L., et al. (2017). BLISS is a versatile and quantitative method for genome-wide profiling of DNA double-strand breaks. Nat. Commun. *8*, 15058.

Yang, Y., Gao, Y., Mutter-Rottmayer, L., Zlatanou, A., Durando, M., Ding, W., Wyatt, D., Ramsden, D., Tanoue, Y., Tateishi, S., and Vaziri, C. (2017). DNA repair factor RAD18 and DNA polymerase Polκ confer tolerance of oncogenic DNA replication stress. J. Cell Biol. *216*, 3097–3115.

Zhang, Y., Liu, T., Meyer, C.A., Eeckhoute, J., Johnson, D.S., Bernstein, B.E., Nusbaum, C., Myers, R.M., Brown, M., Li, W., and Liu, X.S. (2008). Model-based analysis of ChIP-Seq (MACS). Genome Biol. *9*, R137.

Zhang, H., Gan, H., Wang, Z., Lee, J.H., Zhou, H., Ordog, T., Wold, M.S., Ljungman, M., and Zhang, Z. (2017). RPA interacts with HIRA and regulates H3.3 deposition at gene regulatory elements in mammalian cells. Mol. Cell *65*, 272–284.

STAR★METHODS

KEY RESOURCES TABLE

REAGENT or RESOURCE	SOURCE	IDENTIFIER
**Antibodies**		
Rabbit polyclonal anti-Phospho-Histone H2A.X (Ser139)	Abcam	Cat#ab2893; RRID: AB_303388
Rabbit monoclonal anti-Phospho-Histone H3 (Ser10)	Cell Signaling Technology	Cat#3377S; RRID: AB_1549592
Rabbit polyclonal anti-Histone H3	Abcam	Cat#ab1791; RRID: AB_302613
Rabbit polyclonal anti-Histone H2A.X	Merck-Millipore	Cat#07-627
Rabbit polyclonal anti-Histone H3	Cell Signaling Technology	Cat#9715S; RRID: AB_331563
Mouse monoclonal anti-BrdU	Sigma-Aldrich	Cat#B8434; RRID: AB_476811
Mouse monoclonal anti-Tubulin	The Francis Crick Institute Core Facility	Tat-1
Mouse monoclonal anti-BLM	The Francis Crick Institute Core Facility	BFL103
Mouse monoclonal anti Ser5-RPB1	The Francis Crick Institute Core Facility	4H8
Mouse monoclonal anti-U1 snRNP 70	Santa Cruz Biotechnoloy	Cat#sc-390988
Mouse monoclonal anti-NELF-A	Santa Cruz Biotechnoloy	Cat#sc-365004; RRID: AB_10708864
Mouse monoclonal anti-SPT5	Santa Cruz Biotechnoloy	Cat#sc-133217; RRID: AB_2196394
Rabbit polyclonal anti-RAD51	Millipore	Cat#PC130
Mouse anti-Biotin	Jackson ImmunoResearch	Cat#200-002-211; RRID:AB_2339006
Rabbit anti-Biotin	Bethyl Laboratories	Cat#A150-109A; RRID:AB_67327
HRP-linked Horse anti-mouse IgG	Cell Signaling Technology	Cat#7076S; RRID: AB_330924
HRP-linked Goat anti-rabbit IgG	Cell Signaling Technology	Cat#7074S; RRID: AB_2099233
FITC-conjugated Goat anti-Mouse IgG	Sigma-Aldrich	Cat#F2012; RRID: AB_259456
Alexa Fluor488-conjugated Goat Anti-Rabbit IgG (H+L)	Thermo Fisher Scientific	Cat#A-11070; RRID: AB_2534114
**Chemicals, peptides, and recombinant proteins**		
RNase A	AppliChem	Cat#A2760,0100
SUPERase.In	Thermo Fisher Scientific	Cat#AM2696
BrdU	Sigma-Aldrich	Cat#B5002
EdU	Sigma-Aldrich	Cat#900584
Nocodazole	Sigma-Aldrich	Cat#M1404
Ro 3306	Adooq Bioscience	Cat#A14437
Pepsin	Sigma-Aldrich	Cat#P6887
Propidium iodide	Sigma-Aldrich	Cat#P4170
Violet Stain	Thermo Fisher Scientific	Cat#V35003
DRB	Sigma-Aldrich	Cat#D1916
Triptolide	Sigma-Aldrich	Cat#T3652
Quick Start Bradford protein 1x dye reagent	Bio-Rad	Cat#5000205
Agencourt AMPure XP	Beckman Coulter	Cat#A63881
Dynabeads Protein A	Thermo Fisher Scientific	Cat#10002D
Fluoroshield with DAPI	GeneTex	Cat#GTX30920
INTERFERin siRNA Transfection Reagent	Polyplus	Cat#409-10
ATM inhibitor	Stratech	Cat#KU-55933
ATR inhibitor	Stratech	Cat#AZD6738

(*Continued on next page*)

Continued		
REAGENT or RESOURCE	SOURCE	IDENTIFIER
Camptothecin	Sigma-Aldrich	Cat#208925
Etoposide	Sigma-Aldrich	Cat#E1383
CD437	Sigma-Aldrich	Cat#C5865
Rad51 inhibitor (B02)	Sigma-Aldrich	Cat#SML0364
Rad52 inhibitor (AICAR)	Sigma-Aldrich	Cat#A9978
PARP inhibitor (Olaparib)	Adooq Bioscience	Cat#10111
Aphidicolin	Sigma-Aldrich	Cat#A4487
Critical commercial assays		
PureLink Genomic DNA Mini Kit	Beckman Coulter	Cat#A63881
DNA Clean & Concentrator-5	Thermo Fisher Scientific	Cat#10002D
RNeasy Mini Kit	GeneTex	Cat#GTX30920
RNA Clean & Concentrator-5	Polyplus	Cat#409-10
Ribo-Zero Gold rRNA Removal Kit (H/M/R)		Cat#MRZG12324
Random Primed DNA Labeling Kit	Thermo Fisher Scientific	Cat#K182001
Qubit dsDNA HS Assay Kit	Zymo Research	Cat#D4013
SensiFAST SYBR Lo-ROX Kit	QIAGEN	Cat#74106
Click-iT RNA Alexa Fluor 594 Imaging Kit	Zymo Research	Cat#R1015
Nextera XT DNA Library Preparation Kit	Bioline	Cat#BIO-94020
QIAseq Ultralow Input Library Kit	Thermo Fisher Scientific	Cat#C10330
KAPA RNA HyperPrep Kit with RiboErase (HMR)	Sigma-Aldrich	Cat#DUO92002
KAPA Library Quantification Kit	Sigma-Aldrich	Cat#DUO92004
ECL Western Blotting Substrate	Sigma-Aldrich	Cat#DUO92008
SuperScript III Reverse Transcriptase kit	Illumina	Cat#FC-131-1024
Deposited data		
All the datasets generated in this paper	This paper	GSE136294
BJ GRO-Seq	Li et al., 2018	SRX3586216
CTR cells and RNAi of NELFB and NELFE GRO-Seq	Liu et al., 2017	GSE98555
RPE EdU-Seq	Macheret and Halazonetis, 2018	PRJNA397123
U2OS EdU-Seq	Macheret and Halazonetis, 2018	PRJNA397123
CAP-Seq HeLa cells	Tan-Wong et al., 2019	GSE87607
Ok-Seq RPE h-TERT	Chen et al., 2019	GSE114017
Ok-Seq HeLa	Petryk et al., 2016	SRP065949
ChIP-Seq ORC1 HeLa	Dellino et al., 2013	GSE37583
ChIP-Seq MCM7 HeLa	Sugimoto et al., 2018	GSE107248
ChIP-Seq RPA2 HeLa	Zhang et al., 2017	GSE76661
ChOR-Seq mouse ES cells	Stewart-Morgan et al., 2019	GSE128643
HeLa polyA RNA-Seq	Encode Project Consortium	ENCFF343WEZ
ChIP-Seq 53BP1, RAD51, XRCC4 U2OS	Clouaire et al., 2018	E-MTAB-5817
Experimental models: cell lines		
Human osteosarcoma (U2OS)	ATCC	HTB-96
Human immortalized fibroblasts (BJ-hTERT)	ATCC	CRL-4001
Oligonucleotides		
siGENOME Non-Targeting siRNA Pool #2	Dharmacon	Cat#D-001206-14-05
siGENOME Human SUPT5H siRNA Pool	Dharmacon	Cat#M-016234-01-0005
siGENOME Human NELFA siRNA Pool	Dharmacon	Cat#M-012156-00-0005

(*Continued on next page*)

Continued

REAGENT or RESOURCE	SOURCE	IDENTIFIER
Nextera XT Index Kit	Illumina	Cat#FC-131-1001
GeneRead Adaptor I Set A 12-plex (144)	QIAGEN	Cat#180985
SeqCap Adaptor Kit A	Roche	Cat#07141530001
See Table S5 for Real Time-PCR primer list		n/a
Software and algorithms		
EaSeq	Lerdrup et al., 2016	https://easeq.net
R	Friendly, 2002	https://www.r-project.org
Bioconductor	Gentleman et al., 2004	https://bioconductor.org
Bowtie 2	Langmead and Salzberg , 2012	http://bowtie-bio.sourceforge.net/bowtie2/index.shtml
SAMtools	Li et al., 2009	http://www.htslib.org
STAR	Dobin et al., 2013	https://github.com/alexdobin/STAR
BBDuk	Bushnell, BBTools Team	https://github.com/BioInfoTools/BBMap/blob/master/sh/bbduk.sh
featureCounts	Liao et al., 2014	https://www.rdocumentation.org/packages/Rsubread/versions/1.22.2/topics/featureCounts
Galaxy	Afgan et al., 2018	https://usegalaxy.org
MACS2	Zhang et al., 2008	https://github.com/macs3-project/MACS
Bedtools	Quinlan and Hall, 2010	https://bedtools.readthedocs.io/en/latest/
deepTools	Ramírez et al., 2014	https://deeptools.readthedocs.io/en/develop/
pybedtools	Dale et al., 2011	https://daler.github.io/pybedtools/
Prism	GraphPad	Version 7
IGV	Robinson et al., 2011	https://software.broadinstitute.org/software/igv/
FlowJo	Becton Dickinson	Version 10.6.1

RESOURCE AVAILABILITY

Lead contact

Further information and requests for resources and reagents should be directed to and will be fulfilled by the lead contact, Marco Saponaro (m.saponaro@bham.ac.uk).

Materials availability

This study did not generate new unique reagents.

Data and code availability

The accession number for all the genomic data files reported in this paper is GEO: GSE136294.

EXPERIMENTAL MODEL AND SUBJECT DETAILS

Human immortalized fibroblasts (BJ-hTERT cells) were cultured in DMEM (Sigma-Aldrich) supplemented with 10% FBS, 2 mM L-glutamine and penicillin/streptomycin; human osteosarcoma (U2OS cells) were cultured in McCoy's 5A (Modified) Medium (GIBCO) supplemented with 10% FBS, 2 mM L-glutamine and penicillin/streptomycin, both grown in 5% CO_2 at 37°C.

METHOD DETAILS

Growing conditions and DNA Labeling

Cells fully confluent were seeded at 20% confluence in 150 mm dishes, and grown overnight. Cells were synchronized in the G0/G1 phase in serum starvation medium (DMEM supplemented with 0.2% FBS, 2mM L-glutamine and penicillin/streptomycin) in 5% CO_2 at 37°C for 26 h, and released into S-phase with regular DMEM for the indicated time points. 15.5 h after release, 100 ng/ml nocodazole (Sigma-Aldrich) was added to the later time point cell cultures (19 h, 22 h, 24 h, 28 h) to prevent cells from re-entering the cell

cycle. The cells from two 150 mm dishes were incubated for 1 h with 50 μM BrdU before the indicated time point to label the newly replicating DNA. At each time point, labeled cells from two 150 mm dishes were quick washed once with ice-cold PBS and harvested by trypsinization and centrifugation. After washing the cells again with ice-cold PBS and split into three aliquots: 20% of cells were fixed with 70% ethanol for cell cycle analysis; 40% of cells were used for genomic DNA extraction with PureLink Genomic DNA Mini Kit (Thermo Fisher Scientific); 40% of cells were used for cell fractionation and chromatin associated nascent RNA preparation as described below.

For experiments with asynchronous cells, BJ-hTERT and U2OS grown in 150 mm dishes were incubated for 30 min with 50 μM BrdU to label the newly replicating DNA. Cells were quickly washed once with ice-cold PBS and harvested by trypsinization and centrifugation. After washing the cells were fixed with 70% ethanol for cell sorting (see below for details).

BrdU-seq

BrdU labeled genomic DNA (20μg) in 200 μl TE buffer was sonicated using VCX130 (Sonics & Materials, Inc) with the following settings: 6 cycles, 30% Amp, 15 s ON, 25 s OFF. Sonicated DNA was centrifuged at 3000 rpm at 4°C for 5 min and denatured at 100°C for 10 min. Following denaturation, the DNA was put on ice immediately. 15 μl of DNA were mixed with 35 μl of elution buffer (50 mM Tris-HCl pH 8.0, 10 mM EDTA, 1% SDS) as Input. 170 μl of DNA were mixed with 180 μl of 2Xblocking solution (2% BSA, 2XPBS, 0.2% Tween20), 10 μl Dynabeads Protein A (Thermo Fisher Scientific) and 2 μg anti-BrdU antibody (Sigma-Aldrich) in DNA low binding tubes (Sarstedt), incubated at 4°C overnight with rotation. Before mixing with denatured DNA, 10 μl Dynabeads Protein A was prepared by incubating with 2 μg anti-BrdU antibody at 4°C overnight with rotation. After immunoprecipitation, BrdU-labeled DNA bound to beads was washed twice with 1 mL of lysis buffer 1 (50 mM HEPES-KOH pH 7.5, 140 mM NaCl, 1 mM EDTA, 1% Triton X-100, 0.1% Na Deoxycholate), twice with 1 mL of lysis buffer 2 (50 mM HEPES-KOH pH 7.5, 500 mM NaCl, 1 mM EDTA, 1% Triton X-100, 0.1% Na Deoxycholate), twice with 1 mL of wash buffer (10 mM Tris-HCl pH 8.0, 250 mM LiCl,1 mM EDTA, 0.5% Na Deoxycholate, 0.5% Igepal CA-630). The beads were resuspended in 1 mL of TE and centrifuged for 3 min, 3000 rpm at 4°C. The supernatant was removed and beads were resuspended in 50 μl elution buffer and incubated at 65°C for 10 min. The supernatant was transferred into new DNA low binding tubes after centrifugation for 5000 rpm, 1 min at room temperature and leaving the tubes on Magnet (Thermo Fisher Scientific) for 1 min. 50 μl TE and final concentration of 0.5 mg/ml Proteinase K (Promega) were added into the eluted fraction (IP DNA) and incubated at 37°C for 1 h. Meanwhile, 50 μl TE was added to 50 μl Input DNA. Both the IP DNA and Input DNA were purified by DNA Clean & Concentrator-5 (Zymo Research) and eluted twice with 7.5 μl nuclease-free water. Following purification, both the single-stranded BrdU-labeled DNA and the single-stranded Input DNA were converted to double-stranded by Random Primed DNA Labeling Kit (Roche). The double-strand DNA was purified by DNA Clean & Concentrator-5 and eluted twice with 7 μl EB (10 mM Tris-HCl pH 8.5). The purified DNA was quantified with Qubit dsDNA HS Assay Kit (Thermo Fisher Scientific) using a Qubit 3.0 Fluorometer (Thermo Fisher Scientific).

Illumina sequencing libraries were constructed from the random-primed BrdU-DNA material according to Illumina's Nextera XT DNA Library Preparation Kit manufacturer's protocol. After cleaning up, indexed libraries were quantified, normalized and pooled, before sequencing on Illumina NextSeq system in a single lane on a paired-end run.

For the single gene analysis, the IP and Input samples were directly submitted to quantitative RT-PCR using SensiFast SYBR Lo-ROX kit (Bioline) and CFX96 Real-Time System (Biorad) Real-Time PCR. Results shown are average means of three independent experiments, +/− standard error of the mean (SEM).

Chromatin-bound RNA sequencing (Chr-RNA-seq)

Chromatin bound RNA (Chr-RNA) was prepared as previously described (Mayer and Churchman, 2016), with slight modification. The cells which were harvested and resuspended in ice-cold PBS from synchronization were washed once with 1 mL ice-cold PBS. After collecting the cells, we followed Mayer and Churchman (2016) protocol washing twice the nuclei. Cell fractionation quality was assessed by western blotting as in Mayer and Churchman (1996). The chromatin fraction was quick frozen with liquid nitrogen and stored at −80°C for RNA extraction with RNeasy Mini Kit (QIAGEN). 200-300 ng of input RNA in each repeat were depleted of ribosomal RNA with Ribo-Zero Gold rRNA Removal Kit (Illumina) according to manufacturer's guidelines. The recovered RNA was concentrated in 12 μl nuclease-free water using RNA Clean & Concentrator-5 (Zymo Research). The RNA samples were used to construct RNA libraries using KAPA RNA HyperPrep Kit (Kapa Biosystems, Roche) according to manufacturer's guidelines, but without the rRNA depletion step. Instead, 10 μl concentrated RNA was directly mixed in PCR tube with equal volume of Fragment, Prime and Elute Buffer (2X) from KAPA RNA HyperPrep Kit for fragmentation in a thermocycler for 8 min at 94°C. RNA libraries were prepared according to KAPA Kit instruction and sequenced on Illumina NextSeq system in a single lane on a single-end read run.

Nascent transcription levels analysis

For the nascent RNA levels analysis cells were grown as above and at the indicated time points total RNA was extracted with RNeasy Mini Kit (QIAGEN) according to manufacturer instructions. 1 μg of RNA was reverse transcribed with random hexamers with the SuperScript III Reverse Transcriptase kit (Invitrogen). Primers were designed across exon-intron junctions or in intronic regions to monitor specifically nascent pre-mRNA transcription levels, either at the TSS region or in the gene body. Pre-mRNA levels were assessed by quantitative RT-PCR using SensiFast SYBR Lo-ROX kit (Bioline) and CFX96 Real-Time System (Biorad). Results shown are average means of three independent experiments, +/− standard error of the mean (SEM).

Cell cycle analysis

For cell cycle progression analysis cells that were pulsed with BrdU and fixed with 70% ethanol were washed twice with PBS at room temperature. Cells were incubated with 1 mL 2 M HCl (containing 0.1 mg/ml pepsin) for 20 min at room temperature. Cells were washed three times with PBS-T (PBS, 0.2% tween20, 1% goat serum). After the third PBS-T wash, cells were resuspended in 100 μL PBS-T and incubated with 2 μl mouse monoclonal anti-BrdU antibody (Sigma-Aldrich) for 1 h at room temperature. Cells were washed twice with PBS-T, resuspended in 100 μL PBS-T and incubated with 2.5 μL of FITC-conjugated rabbit anti-mouse immunoglobulins (Sigma-Aldrich) in the dark for 1 h at room temperature. Cells were then washed once with PBS-T and resuspended in 0.5 mL PI/RNase A solution (RNaseA 0.1 mg/ml, Propidium iodide 25 μg/ml). Cells were incubated for 5-15 min at 37°C or kept at 4°C overnight before analysis on an Accuri C6 flow cytometer. FACS profiles were analyzed by BD Accuri C6 software.

Transcription activity by EU incorporation analysis by FACS

Global newly synthesized RNA was labeled by 5-ethynyl uridine (EU) 1mM for 1h in BJ-hTERT cells before each indicated time point, and in parallel in a population of asynchronous cells. Cells were fixed with 4% paraformaldehyde (PFA) and permeabilized with 0.5% Triton X-100. The incorporated EU was detected by CytoFLEX Flow Cytometer (Beckman Coulter) after click reaction using Click-iT RNA Imaging Kits (Invitrogen), and cells were stained with Violet DNA in parallel to measure DNA content. Cells were gated in different stages of the cell cycle by DNA content, and EU intensity was analyzed with FlowJo software measuring EU intensity in the different gates in the different time points and comparing EU levels to those of an asynchronous untreated population of cells.

siRNA transfection

siRNA treatment was performed using INTERFERin siRNA Transfection Reagent (Polyplus) following manufacturer's protocol. 37.5 nM of siRNAs from Dharmacon were used to target NELFA, SUPT5H, as well as a control siRNA against Luciferase (see Key resources table), respectively, for indicated transfection time. The efficiency of depletion was tested by western blotting.

Western blotting

For cell fractionation, proteins were prepared from each fraction. For siRNA knockdown, cell lysis was prepared by resuspending cells directly in SDS loading buffer followed by sonication using Bioruptor. Cell extracts were separated by electrophoresis, transferred onto nitrocellulose membranes and blocked in 5% skimmed milk dissolved in 0.1%Tween20/TBS. Membranes were incubated with primary antibodies overnight at 4°C followed by washes in 0.1%Tween20/TBS. Membranes were incubated with appropriate HRP-linked secondary antibodies at room temperature for 1 h and washed three times prior to signal detection. Membranes were developed by chemiluminescence using ECL reagent. Antibodies used were Ser5-P (4H8, 1:10,000), tubulin (Tat-1, 10,000), U1 snRNP70 (sc-390988, 1:1000), Histone H3 (9715S, 1:2000), NELF-A (sc-365004, 1:1000), SUPT5H (sc-133217, 1:1000), HRP-linked Horse anti-mouse IgG (7076S, 1:2000), HRP-linked Goat anti-rabbit IgG (7074S, 1:2000).

Immunostaining

For DRB treated experiments cells were synchronized by serum starvation for 26 h. For the Early S treatment, 15 h after release cells were treated with DMSO or 100 μM DRB (Sigma-Aldrich) for 1 h, respectively, followed by three washes with warm PBS and cultured again in regular DMEM medium. 24 h after release, 9 μM Ro3306 (Adooq Bioscience) were added to the cells for 16 h to arrest cells in G2 similar to *Minocherhomji et al., 2015.* Cells were released into mitosis by vigorous washing (3-4 times for up to 5 min) in pre-warmed PBS. Cells were pulsed with 10 μM EdU (Sigma-Aldrich) in fresh pre-warmed medium for 30 min incubated in 5% CO_2 at 37°C. After one quick wash with ice-cold PBS, cells were fixed and permeabilized for 20 min in PTEMF buffer (20 mM PIPES pH 6.8, 10 mM EGTA, 0.2% Triton X-100, 1 mM $MgCl_2$, 4% formaldehyde) at room temperature. Fixed samples were washed 3 times in PBS and stored at 4°C until use. The 'Click-chemistry' reaction was performed using Click-iT RNA Alexa Fluor 594 Imaging Kit following manufacturer's instructions (Thermo Fisher Scientific). Cells were quick washed with 1 mL rinse buffer and blocked for 1 h at room temperature using 10% FBS/PBS (Sigma Aldrich). Cells were washed 3 times in PBS and incubated with 1:2000 diluted rabbit monoclonal anti-Phospho-Histone H3 (Ser10) (Cell Signaling Technology) in 1% FBS/PBS for 1 h at room temperature. Cells were then washed 3 times in PBS and blocked with 1:1000 diluted Alexa Fluor488-conjugated goat anti-rabbit antibody (Thermo Fisher Scientific) in 1%FBS/PBS for 1 h at room temperature. Cells were then washed 3 times in PBS and mounted with a drop of mounting medium with DAPI (GeneTex). Cells were imaged as described above.

For the analysis of G-MiDS levels after DRB treatment, cells were grown as above and treated for 1h before release in DMSO or DRB (100 μM), followed by an additional treatment of 30' after the Ro3306 release in the presence of EdU. For the analysis of aberrant mitosis, cells were grown as above and treated for 1h before release in DMSO or DRB (100 μM), followed by an additional treatment of 80 min after the Ro3306 release. After one quick wash with ice-cold PBS, cells were fixed and permeabilized for 20 min in PTEMF buffer (20 mM PIPES pH 6.8, 10 mM EGTA, 0.2% Triton X-100, 1 mM $MgCl_2$, 4% formaldehyde) at room temperature. Fixed samples were washed 3 times in PBS and stored at 4°C until use. The cells were quick washed with 1 mL rinse buffer and blocked for 1 h at room temperature using 10% FBS/PBS (Sigma Aldrich). Cells were washed 3 times in PBS and incubated with 1:200 diluted mouse monoclonal anti-Bloom (BLM, Cell Services, The Francis Crick Institute) in 1% FBS/PBS for 1 h at room temperature. Cells were then washed 3 times in PBS and blocked with 1:1000 diluted Alexa Fluor488-conjugated goat anti-mouse antibody (Thermo Fisher Scientific) in 1% FBS/PBS for 1 h at room temperature. Cells were then washed 3 times in PBS and mounted with a drop of mounting medium with DAPI (GeneTex).

For siRNA transfection experiments, at the beginning of serum starvation, transfection was performed as described in the corresponding method section. The transfected cells were synchronized for 26 h and released in regular DMEM medium. 24 h after release, 9 μM Ro3306 were added to the cells for 16 h to arrest cells and processed as described above.

For the Rad52 inhibitor test experiment, asynchronous cells were first treated with 0.4uM aphidicolin (Sigma-Aldrich) and Ro-3306 for 16 h, then released into medium containing EdU and together with either DMSO (control) or 20 μM Rad52i (AICAR, Sigma, CAS 2627-69-2) for 30 min, followed by fixation and EdU Click-It as above. For the Rad51 inhibitor test experiment, asynchronous cells were treated with 1 μM PARP inhibitor (Olaparib, Adooq Bioscience, CAS:763113-22-0) and together with either DMSO (control) or 25 μM Rad51 inhibitor (BO2 Sigma, CAS: 1290541-46-6) for 24 h. The cells were incubated for 5 min in ice-cold extraction buffer (10 mM PIPES, 300 mM Sucrose, 20 mM NaCl, 3 mM $MgCl_2$ and 0.5% Triton X-100) and fixed in 4% PFA (Paraformaldehyde) for 10 min. The cells were washed three times for 5 min in 1X PBS and stored in PBS at 4°C until the day of the staining. Rad51 antibody (Merck) was used in a 1:1000 dilution, followed by Alexa Fluor488-conjugated goat anti-rabbit antibody (Thermo Fisher Scientific) in 1%FBS/PBS for 1 h at room temperature. Cells were then washed 3 times in PBS and mounted with a drop of mounting medium with DAPI as above. Other inhibitors were used as above ATMi (10 μM, KU-55933, Stratech), ATRi (4 μM, AZD6738, Stratech), Camptothecin (10 μM, Sigma-Aldrich), Etoposide (10 μM, Sigma-Aldrich), CD437 (5 μM, Sigma-Aldrich).

ChIP-seq

Asynchronous cells were transfected with 37.5 nM of indicated siRNA for 72 h using the method as described above. Cells with 70%–80% of confluence were harvested by trypsinization. Regular DMEM medium was then added to the cells to inactivate the trypsin. Cells were immediately fixed in 1% formaldehyde for 10 min at room temperature. Then, glycine was added to a final concentration of 0.125 M and the reaction was incubated for 5 min at room temperature. Fixed cells were washed twice with ice-cold PBS, and lysed for 5 min in ice-cold ChIP cell lysis buffer (5 mM HEPES pH 8.0, 85 mM KCl, 0.5% NP-40 alternative, protease inhibitors). Nuclei were pelleted by centrifugation at 3900 g for 5 min at 4°C, and lysed for 5 min in ChIP nuclear lysis buffer (50 mM Tris-HCl pH 8.0, 10 mM EDTA, 1% SDS, protease inhibitors) and incubated 5 minutes on ice. Lysates were sonicated using VCX130 (Sonics & Materials, Inc) with the following settings: 8 cycles, 30% Amp, 15 s ON, 25 s OFF. Sonicated chromatin was centrifuged at 17000 g at 4°C for 10 min and the supernatant was stored at −80°C until immunoprecipitation. Before immunoprecipitation, 15 μl of Dynabeads Protein A was washed twice with 900 μl 5 mg/ml BSA in PBS and reacted for 1 h or more with 3 μg of the corresponding antibody (Phospho-Histone H2A.X: ab2893, Abcam; H2A.X: 07-627, Merck Millipore; H3: ab1791, Abcam) in 500 μl of 5 mg/ml BSA in PBS at room temperature. The beads were washed again with 900 μl 5 mg/ml BSA in PBS before use. The concentration of sonicated chromatin was quantified by using Quick Start Bradford protein 1x dye reagent (Bio-Rad). 50 μl chromatin were used as Input. 0.6 mg of chromatin was 1:5 diluted with ChIP dilution buffer (0.01% SDS, 1.1% Triton X-100, 1.2 mM EDTA, 16.7 mM Tris-HCl pH 8.0, 167 mM NaCl, protease inhibitors), and incubated with prepared beads overnight at 4°C with rotation. Chromatin bound to beads was washed twice in ChIP low salt buffer (0.1% SDS, 1% Triton X-100, 2 mM EDTA, 20 mM Tris-HCl pH 8.0, 150 mM NaCl), twice in ChIP high salt buffer (0.1% SDS, 1% Triton X-100, 2 mM EDTA, 20 mM Tris-HCl pH 8.0, 500 mM NaCl), twice in ChIP LiCl buffer (10 mM Tris-HCl pH 8.0, 250 mM LiCl, 1% NP40 alternative, 1% sodium deoxycholate, 1 mM EDTA), and once in TE buffer (10 mM Tris-HCl, 1 mM EDTA). Most of the TE buffer was removed after the beads were centrifuged at 3000 rpm for 3 min and put on Magnet. The remaining TE was removed after the beads were centrifuged again at 13200 rpm for 1 min and put on Magnet. The beads were resuspended in 50 μl elution buffer (50 mM Tris-HCl pH 8.0, 10 mM EDTA, 1% SDS) and incubated at 65°C for 10 min followed by centrifugation at 12000 rpm for 1 min. The supernatant was transferred to a new DNA low binding tube (Sarstedt) and incubated at 65°C overnight for de-crosslinking. Reverse cross-linked supernatant was mixed with 50 μl of TE and incubated with RNase A at 37°C for 1 h. The supernatant was further incubated with 0.7 mg/ml proteinase K at 55°C for 2 h. DNA was purified by DNA Clean & Concentrator-5 and eluted with 8 μl EB (10 mM Tris-HCl pH 8.5). The purified DNA was quantified with Qubit dsDNA HS Assay Kit (Thermo Fisher Scientific) using a Qubit 3.0 Fluorometer (Thermo Fisher Scientific). 1 ng of DNA was used to construct DNA library according to Illumina's Nextera XT DNA Library Preparation Kit protocol. After being cleaned up, indexed libraries were quantified, normalized and pooled, sequenced on Illumina NextSeq system in a single lane on a paired-end run.

Cell sorting by flow cytometry and G2/M BrdU-seq

Cells with 20% confluence and grown in 150 mm dishes were synchronized by serum starvation. At the start of serum starvation, two-150 mm dishes of cells were transfected with each indicated siRNA as described in the corresponding method. In parallel, two-150 mm dishes of cells with no siRNA transfection were synchronized. In the following steps, both transfected and not-transfected cells were processed in the same way. The cells were synchronized for 26 h and released in regular DMEM medium. 24 h after release, 9 μM Ro3306 were added to the cells for 16 h to arrest cells in G2. Then cells were pulsed with both 25 μM EdU (Sigma-Aldrich) and 50 μM BrdU (Sigma-Aldrich) in fresh pre-warmed medium for 30 min in 5% CO_2 at 37°C. For the Repli-Seq experiment and the sorting of G2/M cells from asynchronous populations of BJ-hTERT and U2OS cells, cells were pulsed for 30 min with 50 μM BrdU. After one-time quick wash with ice-cold PBS, cells were collected by trypsinization followed by mixing with ice-cold regular DMEM to inactivate trypsin. Cells were washed once with ice-cold PBS and fixed and permeabilized for 20 min in PTEMF buffer at room temperature. Fixed cells were washed twice in PBS and stored in PBS at 4°C until use. Asynchronous cells that were pulsed with 25 μM EdU for 30 min in 5% CO_2 at 37°C were also trypsinized, fixed with PTEMF, washed with PBS and stored in PBS until use.

CellPress
OPEN ACCESS

Collected cells from above were immunostained for cell sorting. For color compensation, one aliquot of fixed asynchronous cells were treated with RNase A and used as no dye control; one aliquot of fixed asynchronous cells were treated with RNase A and stained only with Vybrant DyeCycle Violet Stain (Thermo Fisher Scientific) to measure DNA content; one aliquot of fixed asynchronous cells were treated with RNase A and clicked with Alexa Fluor 594 azide to measure DNA synthesis; one aliquot of fixed synchronous but no-siRNA transfected cells were immunostained with Phospho-Histone H3 (Ser10). For cell sorting, one aliquot of each fixed transected cells were immunostained for EdU (clicked with Alexa Fluor 594 azide), Phospho-Histone H3 (Ser10) (with Alexa Fluor488-conjugated goat anti-rabbit antibody), treated with RNase A and co-stained with Violet Stain for cell cycle analysis. The rest of each fixed transected cells and non-transfected cells were immunostained for Phospho-Histone H3 (Ser10) (with Alexa Fluor488-conjugated goat anti-rabbit antibody), treated with RNase A and co-stained with Vybrant DyeCycle Violet Stain for cell sorting based on the positive Phospho-Histone H3 signal and in G2 by DNA content. Cells were sorted using a BD FACSAria cell sorter machine (Beckton Dickinson, USA). Sorted cells were pelleted using centrifugation, resuspended in 200 μl TE and transferred into DNA low binding tube. Cells were sonicated using VCX130 (Sonics & Materials, Inc) with the following settings: 5 cycles, 30% Amp, 15 s ON, 10 s OFF. Sonicated chromatin was briefly centrifuged and incubated at 65°C overnight for de-crosslinking. Reverse crosslinked supernatant was further incubated with 0.7 mg/ml proteinase K at 55°C for 3 h. DNA was purified by DNA Clean & Concentrator-5 and eluted twice with 10 μl EB (10 mM Tris-HCl pH 8.5). The purified DNA was quantified with Qubit dsDNA HS Assay Kit (Thermo Fisher Scientific) using a Qubit 3.0 Fluorometer (Thermo Fisher Scientific). DNA was used to perform BrdU pull-down as described above. After both the single-stranded BrdU-DNA and single-stranded Input DNA were converted to double-strand by Random Primed DNA Labeling Kit (Roche), the double-strand DNA was purified by DNA Clean & Concentrator-5 and eluted once with 7 μl EB (10 mM Tris-HCl pH 8.5). The purified DNA was quantified with Qubit dsDNA HS Assay Kit (Thermo Fisher Scientific) using a Qubit 3.0 Fluorometer (Thermo Fisher Scientific). Illumina sequencing libraries were constructed with 250 pg (or as much as available) of the random-primed BrdU-DNA material according to standard procedures. QIAseq Ultralow Input Library Kit protocol (QIAGEN) was used. After being cleaned up, indexed libraries were quantified, normalized and pooled, sequenced on Illumina NextSeq system in a single lane on a single-end run.

Datasets alignment

Paired-end BrdU-seq reads were aligned to the hg38 genome assembly using Bowtie 2 v.2.3.3.1 (Langmead and Salzberg , 2012). BAM files were sorted and indexed using SAMtools v.1.4 (Li et al., 2009). The reads that mapped to region DAC blacklisted for mappability by the ENCODE project were removed. Single-end RNA-seq data were aligned to the hg38 genome assembly using STAR v.020201 (Dobin et al., 2013) with options "–alignIntronMax 500000," "–outFilterScoreMinOverLread 0.3" and "–outFilterMatchNminOverLread 0.3." Reads were trimmed to remove RNA contamination via BBDuk (Bushnell, JGI BBTools Team). BAM files were sorted and indexed using SAMtools v.1.4 (Li et al., 2009). Counts for each transcribed gene were computed by featureCounts (Liao et al., 2014) using the annotation of the GENCODE genes (GRCh38.p10). Read per million (RPM) were calculated over each gene in each replicate, and genes that had an RPM > 1 in each time point and each replicate were considered as those transcribed in our system. RPM over genes were averaged for the two replicates and used in the further analysis conducted using Bioconductor (Gentleman et al., 2004). Single-end BrdU-Seq data from G2/M DNA synthesis samples were aligned to the hg38 genome using Bowtie 2 v.2.3.4.2 on the online platform Galaxy (https://usegalaxy.org; Afgan et al., 2018). MCM7 (Sugimoto et al., 2018), RPA2 (Zhang et al., 2017) and ORC1 (Dellino et al., 2013) ChIP-Seq for HeLa cells read files, Ok-Seq from RPE (Chen et al., 2019), Ok-Seq from HeLa cells (Petryk et al., 2016), EdU-Seq from RPE and U2OS cells (Macheret and Halazonetis, 2018), CAP-Seq (Tan-Wong et al., 2019) were mapped to hg38 using Bowtie2 v.2.3.4.2 on the online platform Galaxy; ChOR-Seq data from Stewart-Morgan et al. (2019) were mapped to mm10 using the online platform Galaxy (https://usegalaxy.org; Afgan et al., 2018).

Correlation

The Pearson correlation coefficients between replicates and the time points, for each RNA-seq sample, were calculated using the library corrplot in R (Friendly, 2002).

Peak calling

Peaks were called in BrdU samples against the Input DNA using MACS2 v.2.1.0 (Zhang et al., 2008) with human genome size and the following parameters: -m 8 30, -p 0.00001). Around 18,000 peaks were called in Early S; 9,000 in Early/Mid S; 3,000 in Mid S; 1,500 in Late S and 2,700 in Late S/G2. The -intersectBed command of Bedtools software (Quinlan and Hall, 2010) was used to get the overlaps for the transcribed genes and peaks in each replication time point.

Similarly, G-MiDS peaks were called against the Input DNA using MACS2 v.2.1.1 with the same parameters listed above on the online platform Galaxy (https://usegalaxy.org; Afgan et al., 2018). The betools intersect intervals function on the online platform Galaxy was used to identify the G-MiDS specific peaks against the BrdU peaks called in all the S-phase time points combined together. This resulted in approximately 16600 G-MiDS specific peaks.

Replication and transcription reciprocal directionality

To analyze genome-wide replication timing in BrdU-Seq samples, we used a rapid and robust protocol previously described (https://github.com/tobiasrausch/repliseq; Rausch et al., 2018), with a 10 kb bin window. Further analysis was conducted using

an in-house script in R to get the directionality of replication (head to head, codirectional and transition) and to define Watson and Crick strands.

Normalized profiles

Read coverage was calculated over the region ± 2.5 kb around the TSS of all the UCSC annotated genes for GRCh38 genome. The reads that mapped to region DAC blacklisted for mappability by the ENCODE project were removed. The scores per genome regions were used to construct a plot of the average TSS profile using deepTools (Ramírez et al., 2014). In parallel read coverage profiles were generated also using the computational environment EaSeq version 1.101, normalizing the BrdU-Seq file to the Input DNA file with the function "average" (Lerdrup et al., 2016). This approach was also used to plot the average coverage for the gene length (4 categories where consider: genes length < 5 kb; > 5 and < 30 kb; > 30 and < 100 kb; and > 100 kb); for the transcription level (100%–75%; 75%–50%; 50%–25%; 25%–0%), and for the BrdU-seq reads over the different time points, as well as for the majority of the metagene profiles presented in the paper.

Heatmaps were generated with the function "HeatMap" of EaSeq around TSS ± 10 kb using the '+' and '-' split Chr-RNA-Seq datafiles generated mapping reads to the Watson and the Crick strand using the Perl module bam_split.pl on https://metacpan.org/pod/Bio::ViennaNGS (Wolfinger et al., 2015). TDF profiles were generated using igvtools on IGV (Robinson et al., 2011) with a window size of 10bp, and window functions of mean.

ChIP-seq and RNA-seq quantifications

ChIP-Seq levels for γH2AX and for histone H2AX were obtained using the function "quantify" in EaSeq, from the start to the end of the gene of all annotated genes, or only ± 1 kb around TSS, as indicated in the figure legends. The relative level of γH2AX/H2AX was calculated over the gene or the genomic region, averaged for the two replicates and plotted.

Similarly, coordinates of introns were derived from annotated genes list in EaSeq, and Chr-RNA-Seq levels calculated using the function "quantify." The nascent Chr-RNA-Seq levels for each gene were calculated averaging the levels over each intron of that gene, averaged for the two replicates and plotted.

To calculate the P^3R^2 values for BJ cells, we derived the coverage in the region −50/+300 and +300/+1000 for all the transcribed genes, using GRO-Seq data from Li et al. (2018) and the function "quantify" in EaSeq. The ratio between the TSS and gene body regions was calculated, discarding any gene with a coverage of 0 in one of the two regions.

HeLa poly-A RNA-Seq file was downloaded from the Encode consortium (accession number ENCFF343WEZ) and quantified using the feature "featureCounts" on Galaxy. Only genes with FPKM > 1 were considered as transcribed, and filtered by gene length left 1621 genes > 100kb.

To calculate sense and antisense levels around the TSS of genes, the Late S/G2 .bam file was split for reads mapping to the Watson and the Crick strand using the Perl module bam_split.pl on https://metacpan.org/pod/distribution/Bio-ViennaNGS (Wolfinger et al., 2015), and coverage was quantified using the function "quantify" in EaSeq. Similarly, we used the Perl module bam_split.pl to generate the strand specific .bam files for the analysis of the RPE Ok-Seq (Chen et al., 2019), HeLa Ok-Seq datasets (Petryk et al., 2016) and CAP-Seq datasets (Tan-Wong et al., 2019).

QUANTIFICATION AND STATISTICAL ANALYSES

The number of experimental repeats ≥3 is indicated in the figure legends. For the NGS files it was two biological repeats of the full time points sets of BrdU-Seq and Chr-RNA-Seq, two biological repeats for each gH2AX and H2AX ChIP-Seq in CTR siRNA and NELFA and SUPT5H siRNA, two biological repeats of the Repli-Seq experiment with BJ-hTERT, two biological repeats of the G2/M DNA synthesis on BJ-hTERT sorted cells after Ro3306 synchronization in CTR siRNA and NELFA siRNA, one repeat each of the G2/M DNA synthesis on asynchronous BJ-hTERT and U2OS. The repeats were all assessed for correlation before being analyzed together and averaged were specified. Student t test and Mann-Whitney t test were calculated using the software Prism (GraphPad).

Cell Reports, Volume *34*

Supplemental Information

Persistence of RNA transcription during DNA replication delays duplication of transcription start sites until G2/M

Jianming Wang, Patricia Rojas, Jingwen Mao, Martina Mustè Sadurnì, Olivia Garnier, Songshu Xiao, Martin R. Higgs, Paloma Garcia, and Marco Saponaro

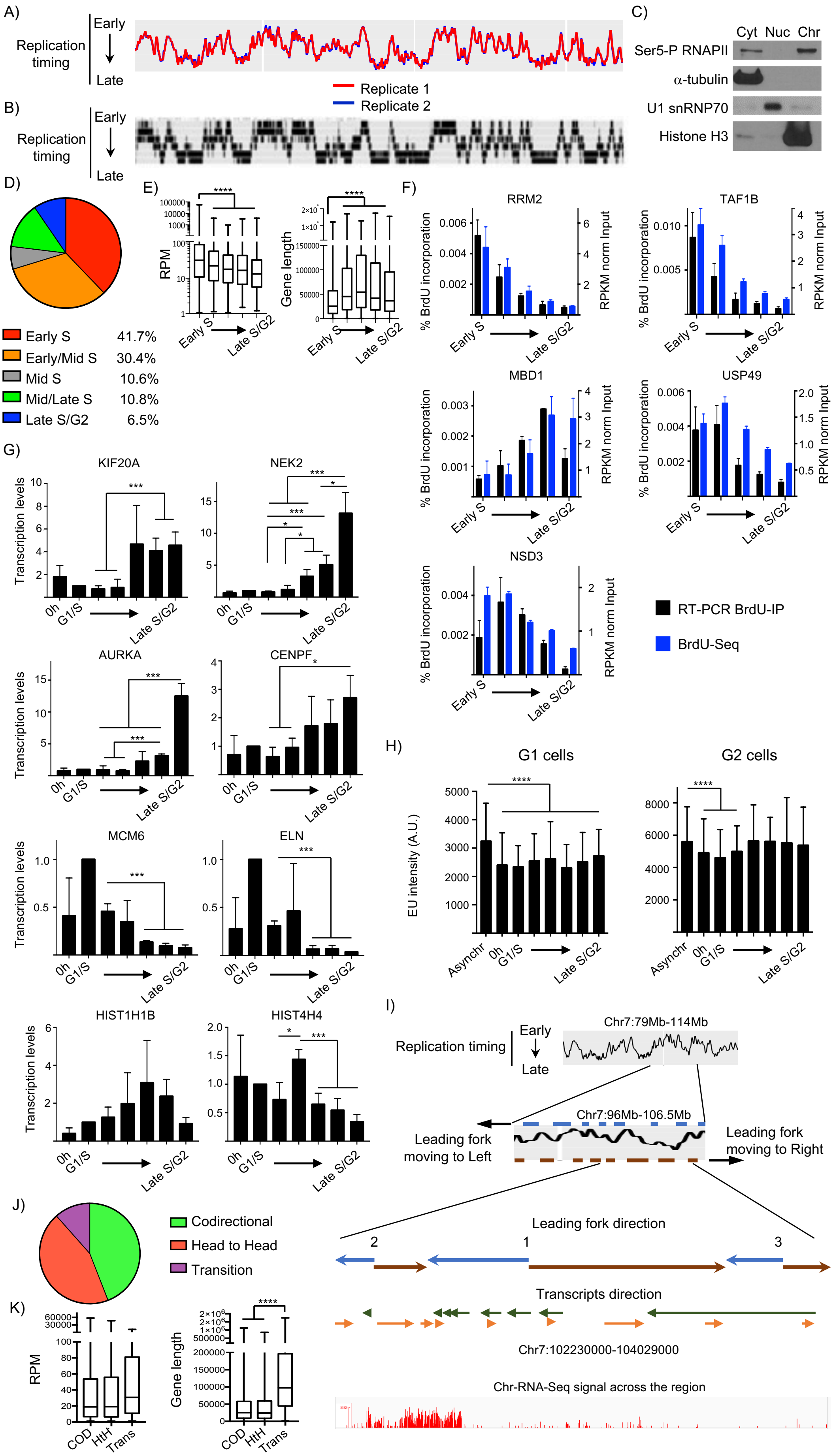

A)
Replication timing
Early
Late
Replicate 1
Replicate 2
B)
Replication timing
Early
Late
C)
Cyt
Nuc
Chr
Ser5-P RNAPII
α-tubulin
U1 snRNP70
Histone H3
D)
Early S 41.7%
Early/Mid S 30.4%
Mid S 10.6%
Mid/Late S 10.8%
Late S/G2 6.5%
E)
RPM
Gene length
Early S
Late S/G2
F)
RRM2
TAF1B
MBD1
USP49
NSD3
% BrdU incorporation
RPKM norm Input
Early S
Late S/G2
RT-PCR BrdU-IP
BrdU-Seq
G)
KIF20A
NEK2
AURKA
CENPF
MCM6
ELN
HIST1H1B
HIST4H4
Transcription levels
0h
G1/S
Late S/G2
H)
G1 cells
G2 cells
EU intensity (A.U.)
Asynchr
0h
G1/S
Late S/G2
I)
Replication timing
Early
Late
Chr7:79Mb-114Mb
Chr7:96Mb-106.5Mb
Leading fork moving to Left
Leading fork moving to Right
Leading fork direction
2
1
3
Transcripts direction
Chr7:102230000-104029000
Chr-RNA-Seq signal across the region
J)
Codirectional
Head to Head
Transition
K)
RPM
Gene length
COD
HtH
Trans

Figure S1. Genomic approaches to characterize transcription and replication together, Related to Figure 1

(A) Replication timing profiles from early to late S phase for the long arm of chromosome 7 shown in Fig 1B, and their reproducibility in two biological replicates. (B) Image adapted from *Hansen et al., 2010*, showing that our approach produces data similar to previously published Repli-Seq ones, but with higher resolution. (C) Representative western blotting analysis for the validation of the cellular fraction strategy in BJ-hTERT cells as in *Mayer et al., 2015*. Subcellular localization markers were α-tubulin for cytoplasm (Cyt), U1 snRNP70 for nucleoplasm (Nuc) and histone H3 for chromatin (Chr), plus transcriptionally engaged Ser5-P RNAPII. (D) Timing of replication for actively transcribed genes (RPM>1). (E) Transcription levels in RPM and gene lengths of transcripts based on replication timing. (F) Single gene analysis of DNA replication levels from Fig 1C compared with the replication levels for the gene from the BrdU-Seq, calculated as reads per kilobase per million (RPKM) normalized to the Input, showing reproducible and consistent replication timing peak between the single gene and the BrdU-Seq analysis; n=3 for the BrdU IP, n=2 for the BrdU-Seq; average mean +/- SEM. (G) Nascent transcription levels presented as fold change (FC) relative to G1/S levels from Fig 1D; n=3; average mean +/- SEM; Student t-test. (H) Transcription activity measured by EU incorporation level followed by Clik-IT; asynchronous and serum starved released cells at the different timepoints are pulsed for 1h with EU before being fixed and analyzed by FACS. EU intensity is quantified for cells gated in G1 and G2 for DNA content and compared to EU incorporation levels in asynchronous growing cells. Average mean +/- Standard Deviation; Student t-test. (I) Procedure to derive reciprocal directionality between the leading replication fork and RNAPII transcription, with a specific example on chromosome (Chr) 7. DNA replication moves from replication origins to replication termination sites. Therefore, in a replication timing profile, the tip in the "inverted V" region (*Hansen et al., 2010*) will represent sites of replication initiation. Sites of replication termination will be instead where two consecutive "inverted V" converge in the replication timing profile. BrdU-Seq is not able to identify specifically DNA replication origins but the combination of all the timepoints together can identify regions where replication initiates. Highlighted in the figure as '1' '2' and '3' are three sites where replication initiates, numbered according to when these origins are activated along the S phase, from early to late. By knowing where the replication initiation sites are and as the leading replication fork progresses from 5' to 3' along the DNA, we can determine whether the leading replication fork is progressing along the Watson or the Crick strand across a specific region. By overlapping the directionality of the replication fork with that of the genes (from the TSS to the TTS), we can define the reciprocal directionality between the leading replication fork and the RNAPII. If both are moving in the same direction we define them as codirectional; if they move in opposite directions they are head to head; if they are in a combination of the two they are defined as transition, because of any of these i) the gene is replicated with replication forks entering from both ends; ii) a replication origin is activated inside the gene iii) the gene contains a replication termination site (see example of long gene in the bottom right, that contains both a replication origin and a replication termination site). Snapshot of the Chr-RNA-Seq data across the region is represented, to show that not all the transcripts present in the region are transcribed. (J) Frequency of codirectional, head to head and transition genes derived as from Fig S1I. (K) Transcription levels and gene lengths of genes in Fig S1J based on their directionality; COD = codirectional; HtH = head to head; Trans = transition; box-whiskers plots with line at the median, Mann-

Whitney t-test; test * => p-value < 0.05; ** => p-value < 0.01; *** => p-value < 0.001; **** => p-value < 0.0001.

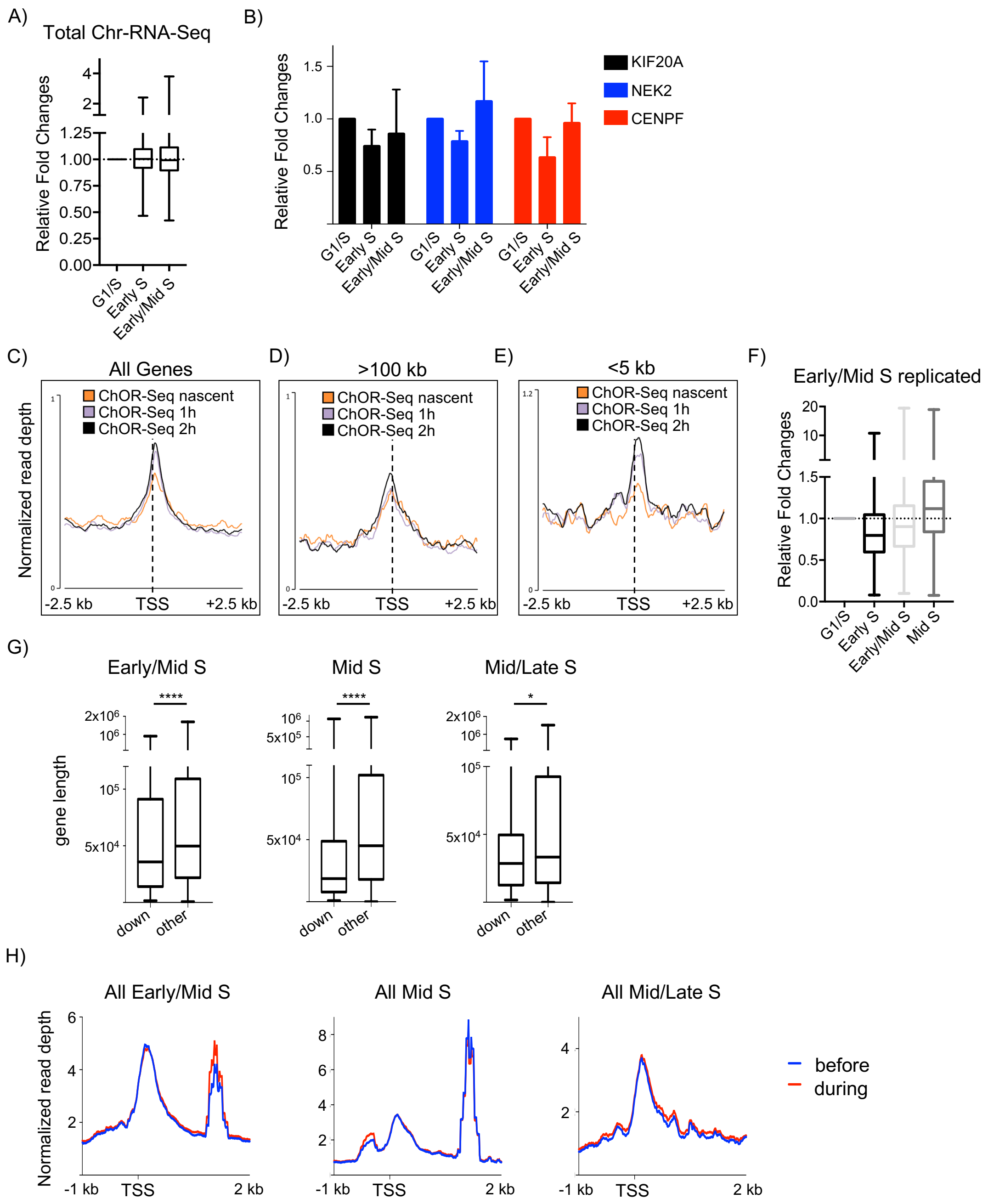

Figure S2. Transcription shut down of genes when they get replicated, Related to Figure 2

(A) As for Fig 2A, relative fold changes of Chr-RNA-Seq levels of Early S replicated genes compared to G1/S and Early/Mid S, over the whole gene including exons. (B) As for Fig 2B for genes replicated in Early S shown in Fig 1D and being more transcribed throughout S/G2. (C-E) ChOR-Seq profiles for Ser5-RNAPII from *Stewart-Morgan et al., 2019* in nascent replicated chromatin, 1 h and 2 h after replication, centered around the TSS +/- 2.5 kb for (C) All Genes in the mouse genome, (D) genes > 100 kb, and (E) genes < 5 kb. (F) As for Fig 2A but for the Early/Mid S replicated genes, showing broader reduced transcription shut down compared to G1/S. (G) Gene length for genes that show a reduction of nascent transcription levels when they get replicated compared to all the other transcripts replicated in the same timepoint; Mann-Whitney t-test; **** => p-value < 0.0001; * => p-value < 0.05. (H) As for Fig 2F for all the genes replicated in each timepoint, comparing average profile in the timepoint they are replicated (during) with the one earlier (before).

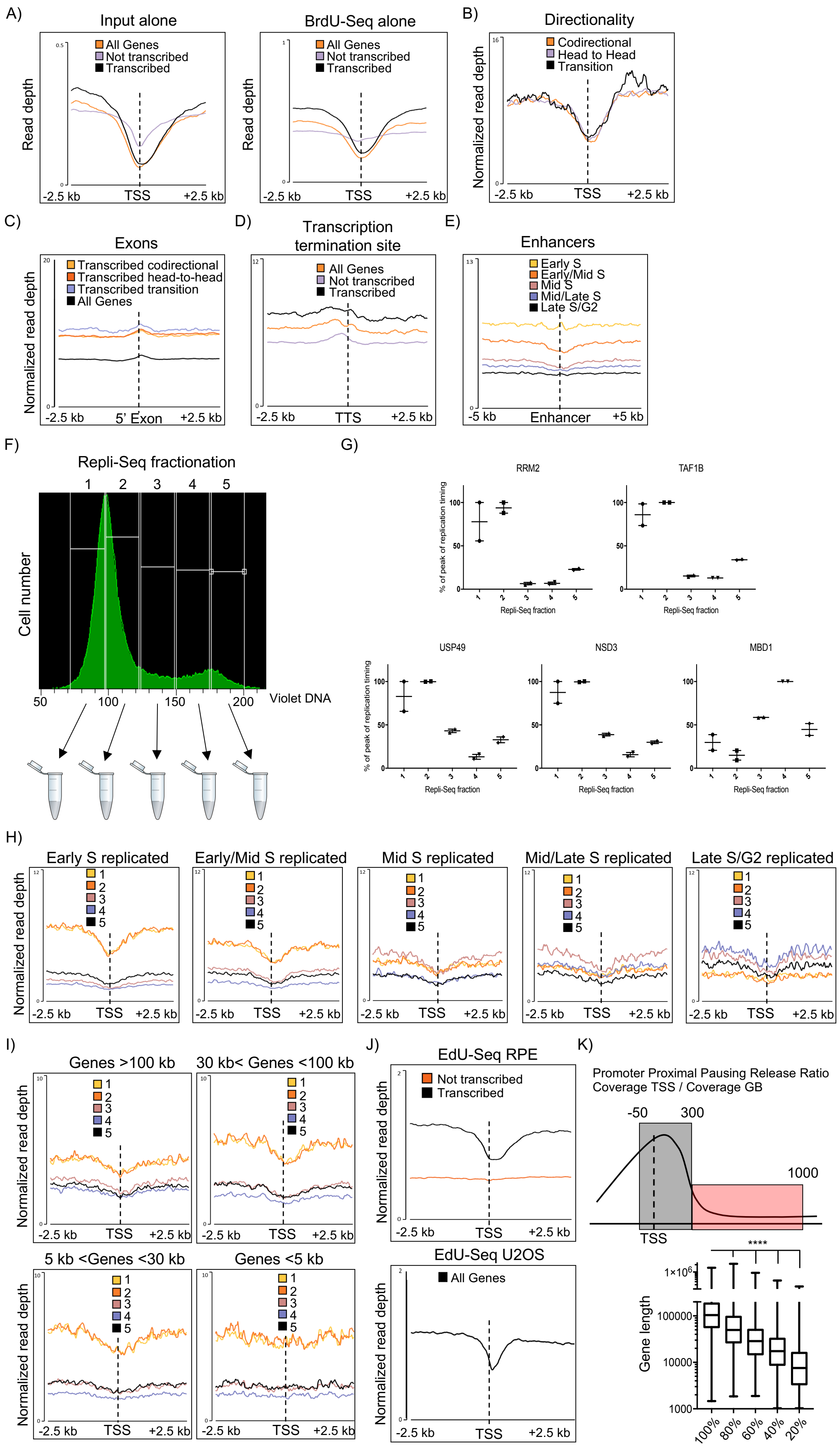
A)
Input alone
Read depth
All Genes
Not transcribed
Transcribed
-2.5 kb
TSS
+2.5 kb
BrdU-Seq alone
Read depth
All Genes
Not transcribed
Transcribed
-2.5 kb
TSS
+2.5 kb
B)
Directionality
Normalized read depth
Codirectional
Head to Head
Transition
-2.5 kb
TSS
+2.5 kb
C)
Exons
Normalized read depth
Transcribed codirectional
Transcribed head-to-head
Transcribed transition
All Genes
-2.5 kb
5' Exon
+2.5 kb
D)
Transcription termination site
All Genes
Not transcribed
Transcribed
-2.5 kb
TTS
+2.5 kb
E)
Enhancers
Early S
Early/Mid S
Mid S
Mid/Late S
Late S/G2
-5 kb
Enhancer
+5 kb
F)
Repli-Seq fractionation
1
2
3
4
5
Cell number
50
100
150
200
Violet DNA
G)
RRM2
TAF1B
% of peak of replication timing
Repli-Seq fraction
USP49
NSD3
MBD1
% of peak of replication timing
Repli-Seq fraction
H)
Early S replicated
Early/Mid S replicated
Mid S replicated
Mid/Late S replicated
Late S/G2 replicated
Normalized read depth
-2.5 kb
TSS
+2.5 kb
I)
Genes >100 kb
30 kb< Genes <100 kb
5 kb <Genes <30 kb
Genes <5 kb
Normalized read depth
-2.5 kb
TSS
+2.5 kb
J)
EdU-Seq RPE
Not transcribed
Transcribed
Normalized read depth
-2.5 kb
TSS
+2.5 kb
EdU-Seq U2OS
All Genes
Normalized read depth
-2.5 kb
TSS
+2.5 kb
K)
Promoter Proximal Pausing Release Ratio
Coverage TSS / Coverage GB
-50
300
1000
TSS
****
Gene length
1×10⁶
100000
10000
1000
100%
80%
60%
40%
20%

Figure S3. TSS of transcribed genes are the only transcriptional pausing hotspots that affect globally DNA replication, Related to Figure 3

(A) As for Fig 3A but of Input and BrdU-Seq signals alone without normalization. (B) As for Fig 3A but with TSS of transcribed genes based on directionality according to Fig S1J. (C) Average metagene profile of BrdU-Seq levels of Early S normalized to Input DNA around the 5'-end of Exons +/- 2.5 kb from all genes and from transcribed genes separated based on their directionality according to Fig S1J. (D) Average metagene profile of BrdU-Seq levels of Early S normalized to Input DNA around the TTS +/- 2.5 kb of all genes, specifically transcribed genes or not transcribed genes. (E) Average metagene profile of BrdU-Seq levels normalized to Input DNA around enhancers mapped in RPE fibroblasts (*Cherry et al. 2018*) for all the timepoints. (F) An asynchronous population of BJ cells is pulsed for 30 min with BrdU before cells are sorted in 5 fractions based on their DNA content by Violet DNA. BrdU-Seq is then performed from each fraction and analyzed. (G) BrdU-Seq levels over the indicated genes in the 5 Repli-Seq fractions quantified as percentage of the fraction with the highest level; n=2; average mean +/- SEM. (H) As Fig 3A with BrdU levels across TSS +/- 2.5kb over genes replicated in the different timepoints from the 5 Repli-Seq fractions. (I) As Fig 3B of the BrdU-Seq level of the 5 Repli-Seq fractions over transcribed genes sorted by gene length. (J) EdU-Seq dataset reanalysis profile across transcribed and not transcribed genes in RPE fibroblasts and for All Genes in U2OS cells (*Macheret et al., 2018*). (K) Schematic on how P^3R^2 is computed, calculating the ratios of the GRO-Seq coverage from *Li et al., 2018* in the region -50/+300 around the TSS and +300/+1000 after the TSS, for all the transcribed genes longer than 1 kb. Gene length for genes according to their P^3R^2, with genes ranked from the highest to the lowest P^3R^2 and divided in 5 groups of equal size; box whiskers plots with line at the median, Mann-Whitney t-test; **** => p-value < 0.0001. Profiles generated using the computational environment EaSeq (*Lerdrup et al., 2016*).

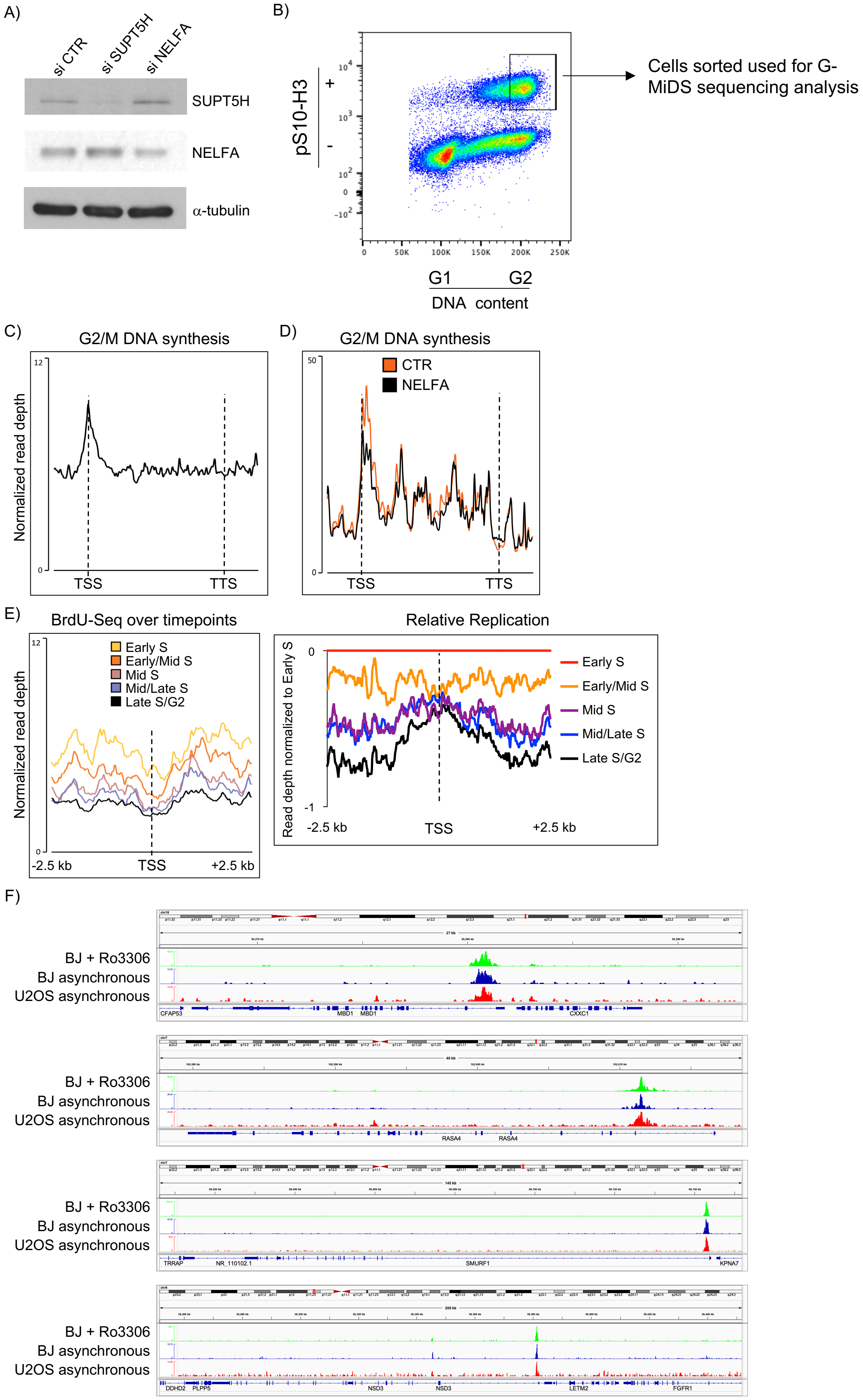
A)
si CTR
si SUPT5H
si NELFA
SUPT5H
NELFA
α-tubulin
B)
pS10-H3
+
-
G1
G2
DNA content
Cells sorted used for G-MiDS sequencing analysis
C)
G2/M DNA synthesis
Normalized read depth
TSS
TTS
D)
G2/M DNA synthesis
CTR
NELFA
TSS
TTS
E)
BrdU-Seq over timepoints
Early S
Early/Mid S
Mid S
Mid/Late S
Late S/G2
Normalized read depth
-2.5 kb
TSS
+2.5 kb
Relative Replication
Read depth normalized to Early S
Early S
Early/Mid S
Mid S
Mid/Late S
Late S/G2
-2.5 kb
TSS
+2.5 kb
F)
BJ + Ro3306
BJ asynchronous
U2OS asynchronous
CFAP53
MBD1
CXXC1
BJ + Ro3306
BJ asynchronous
U2OS asynchronous
RASA4
BJ + Ro3306
BJ asynchronous
U2OS asynchronous
TRRAP
NR_110102.1
SMURF1
KPNA7
BJ + Ro3306
BJ asynchronous
U2OS asynchronous
DDHD2
PLPP5
NSD3
LETM2
FGFR1

Figure S4. TSS of transcribed genes are sites of DNA synthesis in G2/M, Related to Figure 4

(A) Representative western blotting analysis of the siRNA of SUPT5H and NELFA in BJ cells with antibodies against SUPT5H and NELFA, α-tubulin is used as a loading control. (B) Representative image of the mitotic cells sorting strategy by FACS. Cells that are both in G2 by DNA content and pS10-H3 positive (highlighted in the box) are sorted followed by BrdU sequencing. (C) Average binned profile from TSS to TTS for G-MiDS specific BrdU-Seq across all transcribed genes. (D) Average binned profile from TSS to TTS for G-MiDS specific BrdU-Seq across G-MIDS hotspot genes upon for CTR siRNA and after siRNA of NELFA. (E) (left) Average metagene profile of BrdU-Seq levels normalized to Input DNA in all timepoints around the TSS +/- 2.5 kb for the 449 G-MiDS hotspot genes. (right) Relative enrichment for G-MIDS genes of BrdU-Seq levels of all the timepoints compared to the levels in Early S. (F) Snapshots with IGV of the G-MiDS sequencing files over the indicated G-MiDS hotspot TSS for BJ cells synchronized with Ro3306 (green), BJ cells in G2/M sorted from asynchronous cells (blue) and U2OS cells in G2/M sorted from asynchronous cells (red). Profiles generated using the computational environment EaSeq (*Lerdrup et al., 2016*).

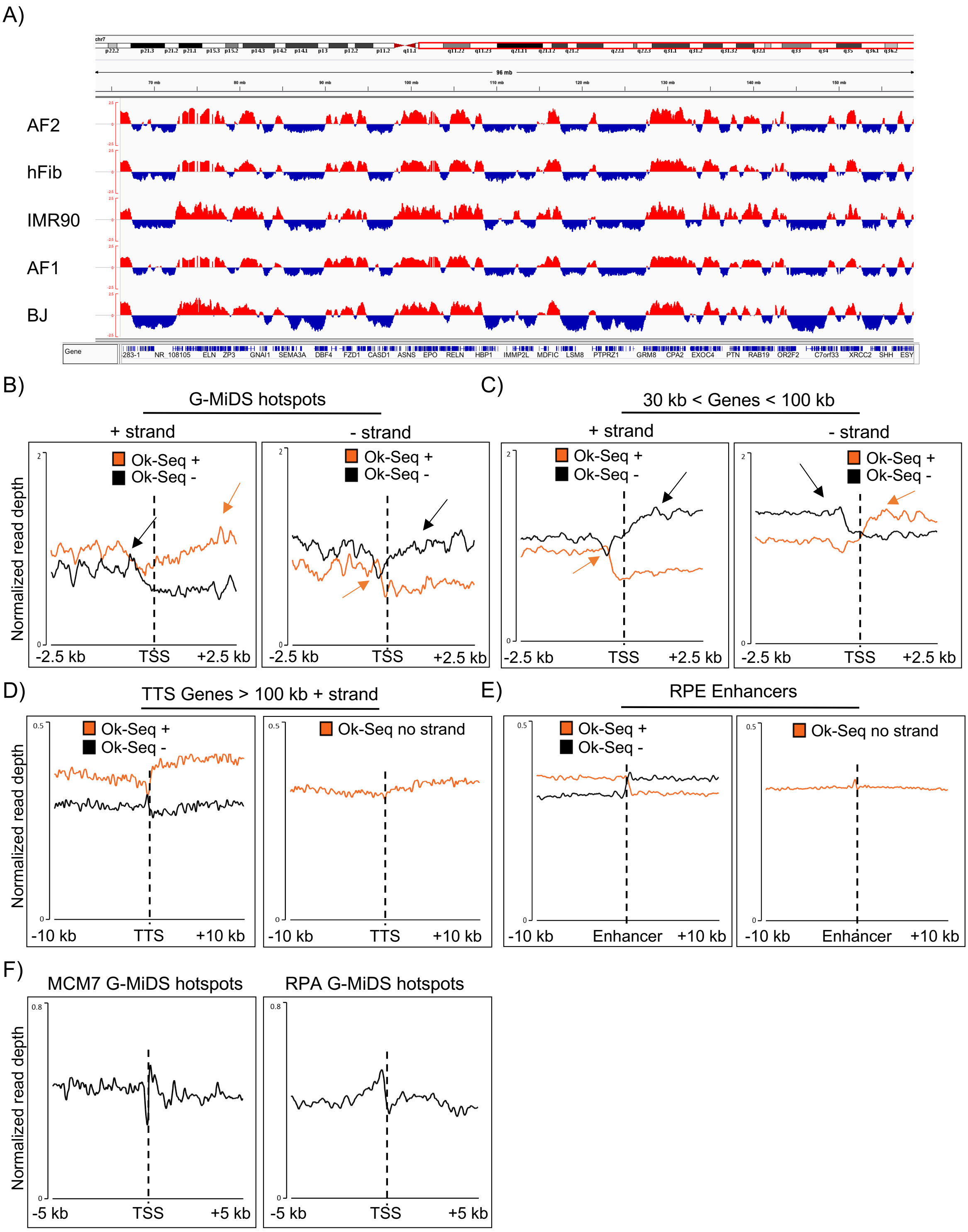

Figure S5 Asymmetric replication fork progression from TSS associated replication origins, Related to Figure 5

(A) IGV snapshot of Repli-Seq profiles from the indicated fibroblasts cell lines, showing how the replication program from early (red) to late (blue) S-phase is conserved among fibroblasts. Data derived from http://www.replicationdomain.com (*Weddington et al., 2008*). (B) Average metagene profile for strand specific Ok-Seq from *Chen et al., 2019* +/- 2.5 kb around the TSS of G-MiDS hotspot genes, either on the '+' or the '-' strand. (C) As for (B) but specifically +/- 2.5 kb around the TSS of transcribed genes > 30 kb and < 100 kb in

BJ-hTERT. (D) Average metagene profile for strand specific Ok-Seq from *Chen et al., 2019* +/- 10 kb around the TTS of transcribed genes > 100 kb in BJ-hTERT, either on the '+' or the '-' strand (left panel), and with no strand specificity (right panel). (E) As for (D) +/- 10kb around enhancers identified in RPE cells as from *Cherry et al., 2018*. (F) As for Fig 5E specifically across the G-MiDS hotspots. Profiles generated using the computational environment EaSeq (*Lerdrup et al., 2016*).

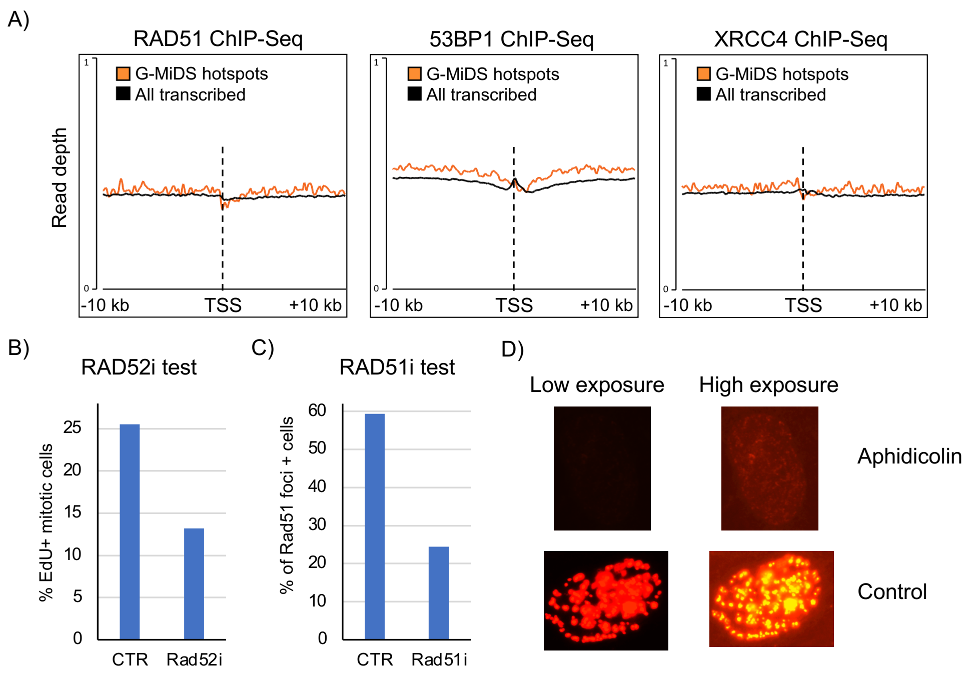

Figure S6 DNA damage levels associated with G-MiDS, Related to Figure 6

(A) Read depth for the ChIP-Seq of RAD51, 53BP1 and XRCC4 in U2OS cells at the TSS +/- 10kb of all transcribed genes and only G-MiDS hotspots from from *Clouaire et al. 2018.* (B) Quantification of EdU foci positive mitotic cells following treatment for 16 h with 0.4 µM aphidicolin and Ro3306, released from the G2 arrest in medium containing EdU for 30 minutes in the presence of DMSO (CTR) or of 20 µM of Rad52 inhibitor (Rad52i). (C) Quantification of Rad51 foci positive cells in cells treated for 24 h with 1 µM of the PARP inhibitor Olaparib, treated in parallel either with DMSO (CTR) or with 25 µM Rad51 inhibitor (Rad51i). (D) Comparison between EdU intensity of G2/M cells in cells untreated, or cells treated with 10 µM aphidicolin once released from the Ro3306 arrest; normal 'Low exposure' or equally amplified 'High exposure'.

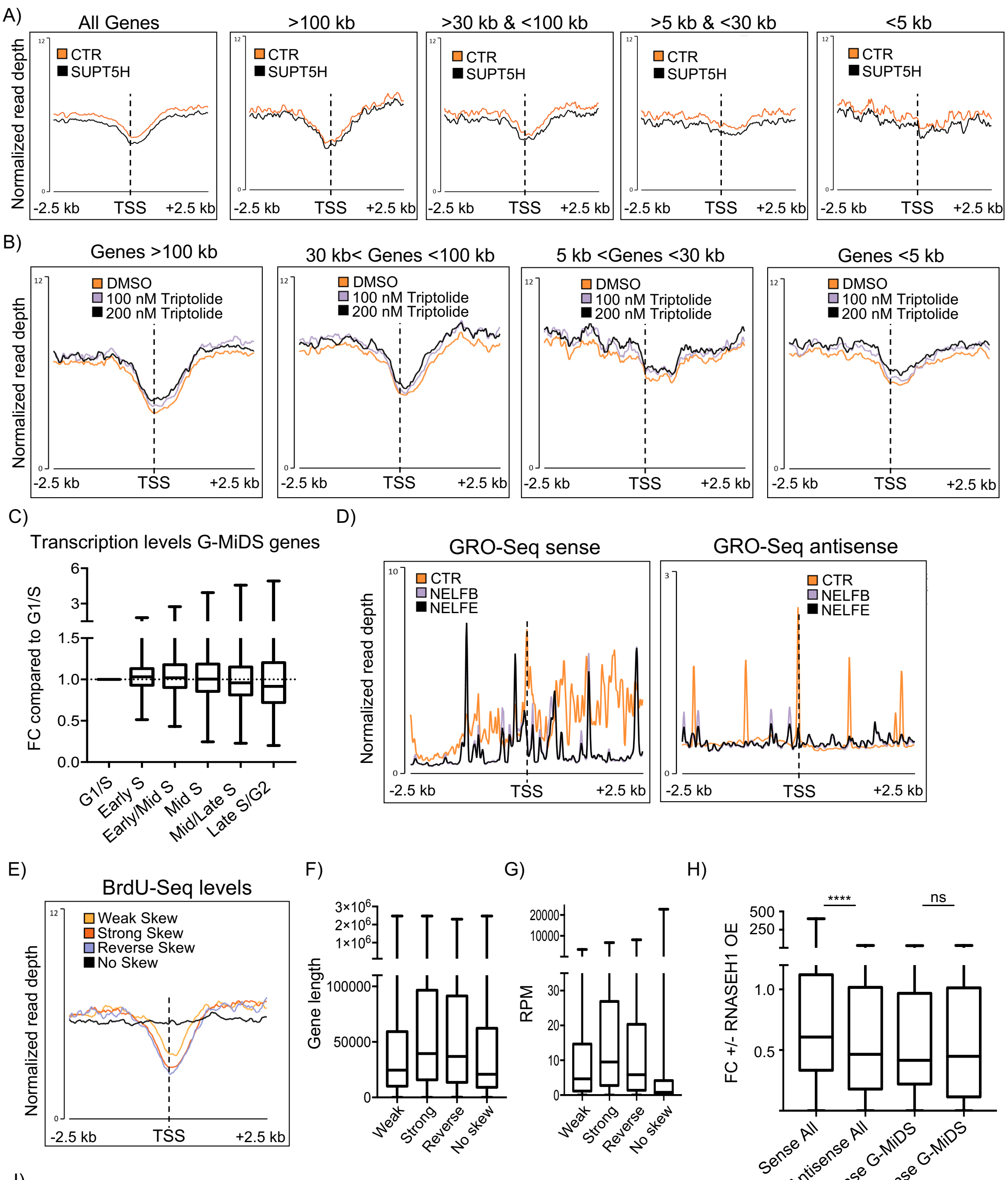

I)

Cancer Gene name			
ARHGAP5	CTNND1	FGFR1OP	RECQL4
ARHGEF10	CUX1	HOXD11	RUNX1T1
CANT1	DICER1	KAT6B	SEPTIN9
CHEK2	DNM2	MAP2K2	TAF15
CIC	EBF1	MECOM	TRIM33
COL3A1	ELN	NCOR2	
CREB3L2	FBLN2	PDE4DIP	

Figure S7. BrdU gaps are dependent more generally on transcription and not only RNAPII; general features of G-MiDS hotspot genes and overlap with Cosmic Cancer Genes, Related to Figure 7

(A) Average metagene profile of BrdU-Seq levels of Early S normalized to Input DNA around the TSS +/- 2.5 kb for all transcribed genes and transcripts separated by gene length in CTR siRNA and after the siRNA of SUPT5H. (B) Average metagene profile of BrdU-Seq levels of Early S normalized to Input DNA around the TSS +/- 2.5 kb in DMSO and cells treated for 1 h with the indicated concentrations of triptolide while being pulsed with BrdU; plots are for transcribed genes separated by gene length. (C) Fold changes in the transcription levels of G-MiDS hotspot genes compared to the G1/S Chr-RNA-Seq timepoint throughout all other timepoints. (D) Average metagene profile of GRO-Seq levels around the TSS +/- 2.5kb of all genes in the human genomes for sense and antisense transcription, in control (CTR) cells and cells after the RNAi of NELFB and NELFE; data derived from *Liu et al., 2017*. (E) Average metagene profile of BrdU-Seq levels of Early S normalized to Input DNA of genes according to promoter class based on GC richness and GC skew as defined in *Ginno et al., 2013*. (F-G) (F) Gene length and (G) and transcription levels for genes based on their promoter class as defined in *Ginno et al., 2013*. (H) Quantification of Cap-Seq levels from *Tan-Wong et al., 2019* at the TSS +/- 1 kb in a strand specific manner across ALL the transcribed genes in BJ-hTERT cells and only the G-MiDS hotspots, as fold change (FC) after the overexpression (OE) of RNASEH1. Only genes with a quantification for both sense and antisense transcription have been analyzed; Mann-Whitney t-test; **** => p-value < 0.0001, ns = not significative. (I) List of the G-MiDS hotspot genes identified in the COSMIC database as Cancer Genes, in particular genes with copy number alterations and/or sites of translocations.

Table S1: Gene ontology analysis of transcripts transcribed in the first time point at the G1/S transition, using the online DAVID Bioinformatic Resources (https://david.ncifcrf.gov), with DNA replication highlighted in bold. Related to Figure 1

GO Term	***Counts***	***p-Value***	***Benjamini***
transcription, DNA-templated	1413	1.50E-42	1.40E-38
cell division	304	2.00E-31	9.60E-28
cell-cell adhesion	244	9.80E-31	3.10E-27
rRNA processing	198	6.90E-29	1.60E-25
mitotic nuclear division	221	2.20E-26	4.10E-23
DNA repair	211	3.00E-26	4.70E-23
mRNA splicing, via spliceosome	200	2.30E-25	3.20E-22
proteasome-mediated ubiquitin-dependent protein catabolic process	183	2.50E-23	3.00E-20
protein transport	322	4.20E-23	4.40E-20
regulation of transcription, DNA-templated	1051	1.10E-22	1.00E-19
cellular response to DNA damage stimulus	185	5.70E-22	4.90E-19
translation	218	9.50E-22	7.50E-19
**DNA replication**	**144**	**1.50E-21**	**1.10E-18**

Table S2: Gene ontology analysis of transcripts changing +/-2 Fold Change expression compared to the first time point at the G1/S transition, using the online DAVID Bioinformatic Resources (https://david.ncifcrf.gov). Related to Figure 1.

Term	Count	%	P-Value	Benjamini	
nucleosome assembly	7	15.2	1.10E-07	2.60E-05	**16h UP**
telomere organization	4	8.7	2.00E-05	2.40E-03	52 transcripts
DNA replication-dependent nucleosome assembly	4	8.7	3.30E-05	2.70E-03	
chromatin silencing at rDNA	4	8.7	5.10E-05	3.10E-03	
protein heterotetramerization	4	8.7	7.50E-05	3.70E-03	
negative regulation of gene expression, epigenetic	4	8.7	1.30E-04	5.20E-03	
positive regulation of gene expression, epigenetic	4	8.7	2.40E-04	8.40E-03	
gene silencing by RNA	4	8.7	1.30E-03	4.00E-02	
cellular protein metabolic process	4	8.7	1.60E-03	4.20E-02	
mitochondrial electron transport, NADH to ubiquinone	3	6.5	4.20E-03	9.70E-02	
nucleosome assembly	25	18.9	8.70E-30	5.70E-27	**19h UP**
telomere organization	14	10.6	2.80E-22	9.30E-20	147 transcripts
DNA replication-dependent nucleosome assembly	14	10.6	4.80E-21	1.00E-18	
chromatin silencing at rDNA	14	10.6	4.70E-20	7.80E-18	
protein heterotetramerization	14	10.6	3.30E-19	4.40E-17	
negative regulation of gene expression, epigenetic	14	10.6	4.40E-18	4.80E-16	
positive regulation of gene expression, epigenetic	14	10.6	9.60E-17	1.00E-14	
gene silencing by RNA	14	10.6	2.70E-13	2.30E-11	
cellular protein metabolic process	14	10.6	6.10E-13	4.50E-11	
regulation of gene silencing	7	5.3	2.80E-11	1.80E-09	
chromatin silencing	9	6.8	4.00E-10	2.40E-08	
negative regulation of megakaryocyte differentiation	7	5.3	1.10E-09	5.90E-08	
telomere capping	7	5.3	5.70E-09	2.90E-07	
DNA replication-independent nucleosome assembly	7	5.3	1.30E-08	6.00E-07	
DNA-templated transcription, initiation	7	5.3	1.00E-07	4.50E-06	
double-strand break repair via nonhomologous end joining	8	6.1	1.50E-07	6.40E-06	
CENP-A containing nucleosome assembly	7	5.3	3.10E-07	1.20E-05	
beta-catenin-TCF complex assembly	7	5.3	3.10E-07	1.20E-05	
innate immune response in mucosa	5	3.8	1.80E-05	6.80E-04	
blood coagulation	9	6.8	2.50E-05	8.60E-04	
mitotic nuclear division	10	7.6	3.20E-05	1.00E-03	
antibacterial humoral response	5	3.8	1.80E-04	5.70E-03	
defense response to Gram-positive bacterium	6	4.5	2.20E-04	6.50E-03	
cell division	10	7.6	4.30E-04	1.20E-02	
cell division	42	17	3.60E-29	3.10E-26	**22h UP**
nucleosome assembly	28	11.3	2.50E-27	1.10E-24	299 transcripts
mitotic nuclear division	33	13.4	3.80E-24	1.10E-21	
telomere organization	14	5.7	1.10E-18	2.20E-16	
DNA replication-dependent nucleosome assembly	14	5.7	1.70E-17	2.90E-15	
chromatin silencing at rDNA	14	5.7	1.70E-16	3.10E-14	
negative regulation of gene expression, epigenetic	15	6.1	4.30E-16	5.40E-14	
protein heterotetramerization	14	5.7	1.10E-15	1.20E-13	
chromosome segregation	16	6.5	2.00E-15	1.90E-13	
sister chromatid cohesion	18	7.3	4.20E-15	3.60E-13	
positive regulation of gene expression, epigenetic	14	5.7	3.10E-13	2.40E-11	
gene silencing by RNA	14	5.7	6.70E-10	4.70E-08	
regulation of gene silencing	7	2.8	1.10E-09	7.30E-08	
cellular protein metabolic process	14	5.7	1.40E-09	8.70E-08	
CENP-A containing nucleosome assembly	10	4	1.50E-09	8.60E-08	
chromatin silencing	10	4	2.40E-09	1.20E-07	
mitotic sister chromatid segregation	8	3.2	1.20E-08	5.90E-07	
negative regulation of megakaryocyte differentiation	7	2.8	4.20E-08	2.00E-06	
metaphase plate congression	6	2.4	1.60E-07	7.30E-06	
telomere capping	7	2.8	2.20E-07	9.30E-06	
anaphase-promoting complex-dependent catabolic process	10	4	3.90E-07	1.60E-05	
DNA replication-independent nucleosome assembly	7	2.8	4.90E-07	1.90E-05	
regulation of attachment of spindle microtubules to kinetochore	5	2	6.50E-07	2.40E-05	
spindle organization	6	2.4	8.70E-07	3.10E-05	
attachment of spindle microtubules to kinetochore	5	2	2.30E-06	7.80E-05	
positive regulation of ubiquitin protein ligase activity	5	2	2.30E-06	7.80E-05	
DNA-templated transcription, initiation	7	2.8	3.70E-06	1.20E-04	
mitotic metaphase plate congression	7	2.8	4.40E-06	1.40E-04	
microtubule-based movement	9	3.6	5.20E-06	1.60E-04	
G2/M transition of mitotic cell cycle	11	4.5	5.40E-06	1.60E-04	
double-strand break repair via nonhomologous end joining	8	3.2	9.30E-06	2.60E-04	
innate immune response in mucosa	6	2.4	9.70E-06	2.70E-04	
beta-catenin-TCF complex assembly	7	2.8	1.10E-05	2.90E-04	
mitotic cytokinesis	6	2.4	2.10E-05	5.40E-04	
mitotic spindle organization	6	2.4	2.50E-05	6.20E-04	
regulation of chromosome segregation	4	1.6	8.80E-05	2.10E-03	
regulation of mitotic spindle organization	4	1.6	8.80E-05	2.10E-03	
mitotic spindle midzone assembly	4	1.6	8.80E-05	2.10E-03	
regulation of cell cycle	9	3.6	1.20E-04	2.70E-03	
cell proliferation	15	6.1	1.20E-04	2.80E-03	

regulation of ubiquitin-protein ligase activity involved in mitotic cell cycle	5	2	1.40E-04	3.10E-03	
antibacterial humoral response	6	2.4	1.70E-04	3.60E-03	
regulation of mitotic nuclear division	5	2	1.70E-04	3.60E-03	
cytokinesis	6	2.4	2.50E-04	5.20E-03	
protein ubiquitination involved in ubiquitin-dependent protein catabolic process	9	3.6	4.80E-04	9.70E-03	
defense response to Gram-positive bacterium	7	2.8	5.20E-04	1.00E-02	
positive regulation of cytokinesis	5	2	9.30E-04	1.80E-02	
regulation of meiotic nuclear division	3	1.2	1.40E-03	2.50E-02	
free ubiquitin chain polymerization	3	1.2	1.40E-03	2.50E-02	
negative regulation of ubiquitin-protein ligase activity involved in mitotic cell cycle	6	2.4	1.50E-03	2.80E-02	
mitotic spindle assembly checkpoint	4	1.6	1.60E-03	2.90E-02	
homologous chromosome segregation	3	1.2	2.00E-03	3.50E-02	
positive regulation of ubiquitin-protein ligase activity involved in regulation of mitotic cell cycle transition	6	2.4	2.10E-03	3.50E-02	
retrograde vesicle-mediated transport, Golgi to ER	6	2.4	2.90E-03	4.80E-02	
attachment of mitotic spindle microtubules to kinetochore	3	1.2	3.70E-03	6.00E-02	
cell cycle	9	3.6	4.40E-03	6.90E-02	
antigen processing and presentation of exogenous peptide antigen via MHC class II	6	2.4	4.80E-03	7.30E-02	
regulation of mitotic metaphase/anaphase transition	3	1.2	4.80E-03	7.20E-02	
protein localization to kinetochore	3	1.2	5.90E-03	8.70E-02	
microtubule depolymerization	3	1.2	5.90E-03	8.70E-02	
DNA damage response, signal transduction by p53 class mediator resulting in cell cycle arrest	5	2	6.20E-03	9.00E-02	
blood coagulation	8	3.2	6.40E-03	9.10E-02	
cell division	49	15.3	3.90E-32	4.50E-29	**24h UP**
nucleosome assembly	32	10	7.20E-30	4.10E-27	382 transcripts
mitotic nuclear division	40	12.5	1.90E-28	7.30E-26	
telomere organization	15	4.7	4.00E-19	1.20E-16	
DNA replication-dependent nucleosome assembly	15	4.7	8.80E-18	2.00E-15	
sister chromatid cohesion	21	6.5	6.40E-17	2.10E-14	
chromatin silencing at rDNA	15	4.7	1.10E-16	1.80E-14	
negative regulation of gene expression, epigenetic	16	5	4.70E-16	6.30E-14	
protein heterotetramerization	15	4.7	8.50E-16	1.10E-13	
chromosome segregation	17	5.3	3.60E-15	4.10E-13	
positive regulation of gene expression, epigenetic	15	4.7	3.70E-13	3.80E-11	
gene silencing by RNA	16	5	1.30E-10	1.20E-08	
mitotic cytokinesis	10	3.1	2.90E-10	2.50E-08	
CENP-A containing nucleosome assembly	11	3.4	6.80E-10	5.50E-08	
G2/M transition of mitotic cell cycle	16	5	2.60E-09	2.00E-07	
cellular protein metabolic process	15	4.7	3.10E-09	2.20E-07	
negative regulation of megakaryocyte differentiation	8	2.5	4.70E-09	3.20E-07	
regulation of gene silencing	7	2.2	5.00E-09	3.20E-07	
chromatin silencing	10	3.1	2.10E-08	1.20E-06	
anaphase-promoting complex-dependent catabolic process	12	3.7	2.90E-08	1.70E-06	
telomere capping	8	2.5	3.40E-08	1.90E-06	
microtubule-based movement	12	3.7	3.80E-08	2.00E-06	
innate immune response in mucosa	8	2.5	6.50E-08	3.20E-06	
mitotic sister chromatid segregation	8	2.5	6.50E-08	3.20E-06	
mitotic metaphase plate congression	9	2.8	6.70E-08	3.20E-06	
spindle organization	7	2.2	8.20E-08	3.70E-06	
DNA replication-independent nucleosome assembly	8	2.5	8.80E-08	3.90E-06	
beta-catenin-TCF complex assembly	9	2.8	2.30E-07	9.80E-06	
mitotic spindle organization	8	2.5	2.60E-07	1.10E-05	
metaphase plate congression	6	1.9	5.60E-07	2.20E-05	
DNA-templated transcription, initiation	8	2.5	9.80E-07	3.70E-05	
regulation of attachment of spindle microtubules to kinetochore	5	1.6	1.80E-06	6.50E-05	
antibacterial humoral response	8	2.5	4.10E-06	1.50E-04	
double-strand break repair via nonhomologous end joining	9	2.8	4.80E-06	1.70E-04	
defense response to Gram-positive bacterium	10	3.1	5.60E-06	1.90E-04	
positive regulation of ubiquitin protein ligase activity	5	1.6	6.20E-06	2.00E-04	
cytokinesis	8	2.5	7.40E-06	2.40E-04	
protein localization to kinetochore	5	1.6	1.00E-05	3.10E-04	
regulation of ubiquitin-protein ligase activity involved in mitotic cell cycle	6	1.9	2.10E-05	6.30E-04	
regulation of mitotic nuclear division	6	1.9	2.60E-05	7.60E-04	
cell division	61	8.1	2.70E-24	6.30E-21	**28h UP**
mitotic nuclear division	50	6.6	8.50E-23	1.00E-19	874 transcripts
nucleosome assembly	33	4.4	1.60E-19	1.30E-16	
telomere organization	15	2	7.50E-14	4.40E-11	
sister chromatid cohesion	25	3.3	2.00E-13	9.50E-11	
DNA replication-dependent nucleosome assembly	15	2	1.50E-12	5.90E-10	
protein heterotetramerization	16	2.1	8.10E-12	2.70E-09	
negative regulation of gene expression, epigenetic	17	2.3	1.10E-11	3.30E-09	
chromatin silencing at rDNA	15	2	1.60E-11	4.30E-09	
cellular protein metabolic process	24	3.2	3.30E-11	7.80E-09	
chromosome segregation	18	2.4	2.00E-10	4.40E-08	
G2/M transition of mitotic cell cycle	24	3.2	7.50E-10	1.50E-07	
positive regulation of gene expression, epigenetic	15	2	3.30E-08	6.10E-06	

CENP-A containing nucleosome assembly	12	1.6	2.60E-07	4.30E-05	
mitotic cytokinesis	10	1.3	5.30E-07	8.30E-05	
spindle organization	8	1.1	6.80E-07	1.00E-04	
anaphase-promoting complex-dependent catabolic process	15	2	8.10E-07	1.10E-04	
regulation of gene silencing	7	0.9	8.70E-07	1.10E-04	
retrograde vesicle-mediated transport, Golgi to ER	15	2	1.30E-06	1.60E-04	
metaphase plate congression	7	0.9	1.70E-06	2.00E-04	
mitotic sister chromatid segregation	9	1.2	1.80E-06	2.00E-04	
negative regulation of megakaryocyte differentiation	8	1.1	1.80E-06	1.90E-04	
antibacterial humoral response	11	1.5	2.90E-06	3.00E-04	
chromatin silencing	11	1.5	3.60E-06	3.60E-04	
mitotic metaphase plate congression	10	1.3	5.10E-06	4.80E-04	
gene silencing by RNA	16	2.1	1.10E-05	1.00E-03	
regulation of ubiquitin-protein ligase activity involved in mitotic cell cycle	8	1.1	1.20E-05	1.00E-03	
telomere capping	8	1.1	1.20E-05	1.00E-03	
innate immune response in mucosa	8	1.1	2.10E-05	1.80E-03	
DNA replication-independent nucleosome assembly	8	1.1	2.80E-05	2.30E-03	
negative regulation of ubiquitin-protein ligase activity in mitotic cell cycle	12	1.6	4.60E-05	3.60E-03	
defense response to Gram-positive bacterium	13	1.7	5.40E-05	4.10E-03	
regulation of attachment of spindle microtubules to kinetochore	5	0.7	5.50E-05	4.00E-03	
mitotic spindle organization	8	1.1	7.80E-05	5.60E-03	
positive regulation of ubiquitin-protein ligase activity involved in regulation of mitotic cell cycle transition	12	1.6	8.60E-05	6.00E-03	
beta-catenin-TCF complex assembly	9	1.2	1.30E-04	9.00E-03	
microtubule-based movement	12	1.6	1.60E-04	1.00E-02	
regulation of mitotic nuclear division	7	0.9	1.70E-04	1.10E-02	
positive regulation of ubiquitin protein ligase activity	5	0.7	1.90E-04	1.10E-02	
DNA-templated transcription, initiation	8	1.1	2.60E-04	1.60E-02	
protein localization to kinetochore	5	0.7	3.00E-04	1.80E-02	
protein K11-linked ubiquitination	7	0.9	3.40E-04	1.90E-02	
mitochondrial ATP synthesis coupled proton transport	6	0.8	7.70E-04	4.20E-02	
positive regulation of T cell mediated cytotoxicity	5	0.7	9.40E-04	5.00E-02	
negative regulation of cell proliferation	28	3.7	1.30E-03	6.70E-02	
cytokinesis	8	1.1	1.60E-03	8.10E-02	
mitotic chromosome condensation	5	0.7	1.70E-03	8.30E-02	
DNA damage response, signal transduction by p53 class mediator resulting in cell cycle arrest	9	1.2	1.70E-03	8.20E-02	
cell redox homeostasis	10	1.3	1.80E-03	8.60E-02	
cell proliferation	26	3.4	1.90E-03	8.60E-02	
double-strand break repair via nonhomologous end joining	9	1.2	1.90E-03	8.60E-02	
type I interferon signaling pathway	9	1.2	2.10E-03	9.30E-02	
positive regulation of I-kappaB kinase/NF-kappaB signaling	15	2	2.20E-03	9.50E-02	
regulation of chromosome segregation	4	0.5	2.30E-03	9.80E-02	
mitotic spindle midzone assembly	4	0.5	2.30E-03	9.80E-02	
					**16h DOWN**
No GO Term enrichment					13 transcripts
negative regulation of signal transduction	3	6.8	2.50E-03	5.50E-01	**19h DOWN**
Peyer's patch development	2	4.5	1.10E-02	8.20E-01	49 transcripts
negative regulation of oligodendrocyte differentiation	2	4.5	2.40E-02	9.20E-01	
olfactory bulb development	2	4.5	4.10E-02	9.60E-01	
DNA replication	3	6.8	4.60E-02	9.50E-01	
regulation of cell proliferation	3	6.8	6.30E-02	9.70E-01	
DNA replication initiation	2	4.5	6.80E-02	9.60E-01	
DNA replication initiation	6	5	9.30E-07	6.40E-04	**22h DOWN**
G1/S transition of mitotic cell cycle	8	6.6	1.80E-06	6.10E-04	138 transcripts
DNA replication	8	6.6	2.80E-05	6.40E-03	
negative regulation of Notch signaling pathway	4	3.3	5.80E-04	9.40E-02	
negative regulation of oligodendrocyte differentiation	3	2.5	1.70E-03	2.10E-01	
DNA replication initiation	13	8.8	2.70E-18	2.40E-15	**24h DOWN**
DNA replication	20	13.6	2.90E-18	1.30E-15	166 transcripts
G1/S transition of mitotic cell cycle	17	11.6	2.70E-17	8.00E-15	
regulation of transcription involved in G1/S transition of mitotic cell cycle	7	4.8	1.20E-08	2.70E-06	
DNA replication checkpoint	4	2.7	2.10E-05	3.70E-03	
DNA unwinding involved in DNA replication	4	2.7	4.40E-05	6.50E-03	
DNA replication	33	6.8	3.50E-21	6.40E-18	**28h DOWN**
DNA replication initiation	16	3.3	1.40E-16	1.00E-13	537 transcripts
G1/S transition of mitotic cell cycle	23	4.8	2.50E-15	1.50E-12	
telomere maintenance via recombination	12	2.5	1.00E-10	4.80E-08	
DNA strand elongation involved in DNA replication	7	1.5	7.50E-07	2.80E-04	
DNA synthesis involved in DNA repair	9	1.9	1.30E-06	4.10E-04	
regulation of transcription involved in G1/S transition of mitotic cell cycle	7	1.5	1.30E-05	3.40E-03	
DNA repair	19	3.9	1.50E-05	3.40E-03	
DNA replication checkpoint	5	1	2.10E-05	4.30E-03	
DNA unwinding involved in DNA replication	5	1	6.00E-05	1.10E-02	
DNA duplex unwinding	8	1.7	7.50E-05	1.30E-02	
intracellular signal transduction	24	5	1.10E-04	1.70E-02	
nucleotide-excision repair, DNA gap filling	6	1.2	2.20E-04	3.10E-02	
negative regulation of Notch signaling pathway	6	1.2	5.60E-04	7.20E-02	
folic acid metabolic process	5	1	7.50E-04	8.90E-02	

Table S3: Correlation analysis between the BrdU-Seq and Chr-RNA-Seq time points and repeats. Related to Figure 1.

Correlation between timepoints based on RPM for each gene > 1RPM

	14h	16h	19h	22h	24h
**14h**	1	0.991	0.99	0.978	0.974
**16h**		1	0.988	0.96	0.959
**19h**			1	0.988	0.988
**22h**				1	0.998
**24h**					1

14h = G1/S
16h = Early S
19h = Early/Mid S
22h = Mid S
24h = Mid/Late S
28h = Late S/G2

Correlations between biological repeats based on RPM for each gene >1 RPM

	14h	16h	19h	22h	24h
**14h**	0.987				
**16h**		0.992			
**19h**			0.992		
**22h**				0.996	
**24h**					0.994

Table S4: Gene ontology analysis of G-MiDS hotspot transcripts, using the online DAVID Bioinformatic Resources (https://david.ncifcrf.gov). Related to Figure 7.

/

GO Term	***Counts***	***p-Value***	***Benjamini***
transcription, DNA-templated/	75/	1.30E-06/	1.90E-03/
regulation of transcription, DNA-templated/	62/	1.50E-06/	1.10E-03/
synaptic transmission, glutamatergic/	5/	9.90E-04/	4.00E-01/
positive regulation of protein phosphorylation/	9/	6.70E-03/	9.30E-01/
endocytic recycling/	4/	1.50E-02/	9.90E-01/
fatty acid beta-oxidation/	5/	1.50E-02/	9.80E-01/
positive regulation of transcription, DNA-templated/	20/	1.80E-02/	9.80E-01/
protein targeting to plasma membrane/	4/	1.80E-02/	9.70E-01/
peptidyl-tyrosine phosphorylation/	9/	1.90E-02/	9.60E-01/
regulation of defence response to virus by virus/	4/	2.20E-02/	9.70E-01/
positive regulation of GTPase activity/	21/	2.30E-02/	9.60E-01/
ER to Golgi vesicle-mediated transport/	9/	2.40E-02/	9.60E-01/
regulation of small GTPase mediated signal transduction/	8/	2.80E-02/	9.60E-01/
cell migration/	9/	3.50E-02/	9.80E-01/
positive regulation of protein kinase B signalling/	6/	3.60E-02/	9.80E-01/

/
